# Probing differences among Aβ oligomers with two triangular trimers derived from Aβ

**DOI:** 10.1073/pnas.2219216120

**Published:** 2023-05-22

**Authors:** Adam G. Kreutzer, Gretchen Guaglianone, Stan Yoo, Chelsea Marie T. Parrocha, Sarah M. Ruttenberg, Ryan J. Malonis, Karen Tong, Yu-Fu Lin, Jennifer T. Nguyen, William J. Howitz, Michelle N. Diab, Imane L. Hamza, Jonathan R. Lai, Vicki H. Wysocki, James S. Nowick

**Affiliations:** ^a^Department of Chemistry, University of California Irvine, Irvine, CA 92697; ^b^Department of Pharmaceutical Sciences, University of California Irvine, Irvine, CA 92697; ^c^Department of Biochemistry, Albert Einstein College of Medicine, Bronx, NY 10461; ^d^Resource for Native Mass Spectrometry Guided Structural Biology, The Ohio State University, Columbus, OH 43210

**Keywords:** Aβ oligomers, Alzheimer’s disease, X-ray crystallography, native mass spectrometry, cellular toxicity

## Abstract

The assembly of the β-amyloid peptide (Aβ) to form oligomers and fibrils is closely associated with the pathogenesis and progression of Alzheimer’s disease. Aβ is a shape-shifting peptide capable of adopting many conformations and folds within the multitude of oligomers and fibrils the peptide forms. These properties have precluded detailed structural elucidation and biological characterization of homogeneous, well-defined Aβ oligomers. In this paper, we compare the structural, biophysical, and biological characteristics of two different covalently stabilized isomorphic trimers derived from the central and *C*-terminal regions Aβ. X-ray crystallography reveals the structures of the trimers and shows that each trimer forms a ball-shaped dodecamer. Solution-phase and cell-based studies demonstrate that the two trimers exhibit markedly different assembly and biological properties. One trimer forms small soluble oligomers that enter cells through endocytosis and activate capase-3/7-mediated apoptosis, while the other trimer forms large insoluble aggregates that accumulate on the outer plasma membrane and elicit cellular toxicity through an apoptosis-independent mechanism. The two trimers also exhibit different effects on the aggregation, toxicity, and cellular interaction of full-length Aβ, with one trimer showing a greater propensity to interact with Aβ than the other. The studies described in this paper indicate that the two trimers share structural, biophysical, and biological characteristics with oligomers of full-length Aβ. The varying structural, assembly, and biological characteristics of the two trimers provide a working model for how different Aβ trimers can assemble and lead to different biological effects, which may help shed light on the differences among Aβ oligomers.

## Significance

Oligomers of the β-amyloid peptide (Aβ) contribute to neurodegeneration in Alzheimer’s disease and are promising targets for therapies against Alzheimer’s disease. Understanding the structures and biology of Aβ oligomers is important for understanding the molecular basis of Alzheimer’s disease and for developing better therapeutics and diagnostics for the disease. Aβ oligomers are difficult to study because they are heterogeneous and unstable. To gain insights into Aβ oligomers and their role in neurodegeneration, we create, characterize, and compare two different well-defined chemical models of Aβ oligomers composed of different peptide fragments from Aβ that are constrained into β-hairpin conformations.

Oligomers of the β-amyloid peptide (Aβ) are central to the pathogenesis and progression of Alzheimer’s disease (1–18). Aβ dimers and trimers, as well as higher-order oligomers, such as hexamers and dodecamers and larger oligomers, are thought to have special significance in Alzheimer’s disease (19, 20). Aβ plaques from Alzheimer’s disease individuals contain cross-linked Aβ dimers that are composed of different Aβ alloforms (21). Aβ dimers appear to be the building blocks of large, mildly cytotoxic oligomers (22). Concentrations of Aβ trimers are elevated in cognitively normal adults who are at risk for Alzheimer’s disease (23). Aβ trimers appear to be the building blocks of the putative dodecamer of Aβ, termed Aβ*56, although recent reports have called the identification, characterization, and study of Aβ*56 into question (24–28). Aβ dimers, Aβ trimers, and Aβ*56 promote phosphorylation and aggregation of the microtubule-associated protein tau, which is also involved in Alzheimer’s disease progression (29–31).

While cryo-EM and ssNMR spectroscopy have revealed the structures of many Aβ fibrils (32–48), high-resolution structures of Aβ oligomers are largely unknown, and the relationship between Aβ oligomer structure and Aβ oligomer biology is unclear. The challenges of isolating stable biogenic Aβ oligomers of sufficient quantity and homogeneity or preparing homogeneous Aβ oligomers in vitro have precluded detailed characterization of the relationship between the structures and biological properties of Aβ oligomers.

To study Aβ oligomers, researchers have developed a variety of methods for preparing Aβ oligomers in vitro (49–62). Studies of these in vitro preparations have shown that Aβ can form many oligomers that differ in size, stoichiometry, morphology, toxicity, and ability to form fibrils. The formation and properties of these different Aβ oligomers are highly dependent on the conditions under which the oligomers are prepared. To reduce the heterogeneity among Aβ oligomers, researchers have prepared and studied Aβ oligomers that consist of Aβ monomers linked by chemical cross-links (63–69). These studies have helped determine the importance of different residues in Aβ oligomerization and have demonstrated that different Aβ alloforms form different oligomers. Although cross-linking Aβ decreases the heterogeneity of Aβ oligomers, cross-linking full-length Aβ has not yet produced structurally homogeneous oligomers amenable to high-resolution structural elucidation. X-ray crystallographic studies of fragments of Aβ and other amyloidogenic peptides and proteins, as well as NMR studies of full-length Aβ, have provided clues about amyloid oligomer structures, showing that many amyloid oligomers contain antiparallel β-sheets and packed hydrophobic cores (70–76).

β-hairpins have emerged as important structural motifs in Aβ oligomers and have also been identified as a component of Aβ fibrils (77–86). β-hairpins are the simplest antiparallel β-sheet, comprising two hydrogen-bonded β-strands connected by a loop. Several Aβ β-hairpins have been described in which the central and *C*-terminal regions of the Aβ peptide comprise the β-strands of the β-hairpin. Härd et al. elucidated the NMR structure of an Aβ β-hairpin bound to an affibody (80). Härd et al. subsequently stabilized Aβ_40_ and Aβ_42_ in a β-hairpin conformation by installing a cross-strand intramolecular disulfide bond and demonstrated that these stabilized Aβ β-hairpins assemble to form soluble oligomers that recapitulate many characteristics of Aβ oligomers (81, 82). Tycko et al. elucidated the cryo-EM structure of an Alzheimer’s disease brain-derived Aβ_40_ fibril in which Aβ β-hairpins sandwich an extended core composed of parallel β-sheets (86). Yu et al. used NMR spectroscopy to characterize the structure of an Aβ_42_ dimer that contains two Aβ β-hairpins (83). Carulla et al. described the NMR structures of an Aβ tetramer and octamer, which contain Aβ β-hairpins, in a membrane-mimicking lipid bilayer (84, 85).

The alignments of the β-strands from central and *C*-terminal regions of Aβ differ among the β-hairpins described above. The β-strand alignment within an Aβ β-hairpin is significant because different β-strand alignments have different residue pairings, which leads to variations in the surfaces of the β-hairpin on which the amino acid side chains are displayed. *SI Appendix*, Fig. S1 illustrates the five Aβ β-hairpins described in the preceding paragraph and shows how the relationship between two residues—E_22_ and I_31_—varies among these different alignments. We have previously observed that Aβ β-hairpins with different β-strand alignments assemble to form different oligomers with unique three-dimensional structures (87, 88, 89), and we envision that these different oligomers contribute to the structural and biological differences among Aβ oligomers in Alzheimer’s disease (20).

To study oligomers composed of Aβ β-hairpins with different β-strand alignments, we have developed the following approach: 1) design and synthesize macrocyclic β-hairpin peptides containing two Aβ β-strands that are constrained into a specific alignment, 2) elucidate the structures of the oligomers that the Aβ β-hairpin peptides form using X-ray crystallography, 3) design and synthesize covalently stabilized Aβ oligomer models, and 4) study the structural, biophysical, and biological properties of the Aβ oligomer models (90, 91). Through this approach, we aim to understand whether different Aβ oligomer models exhibit different structural, biophysical, and biological properties and determine whether these models share characteristics with oligomers of full-length Aβ.

**Fig. 1.**
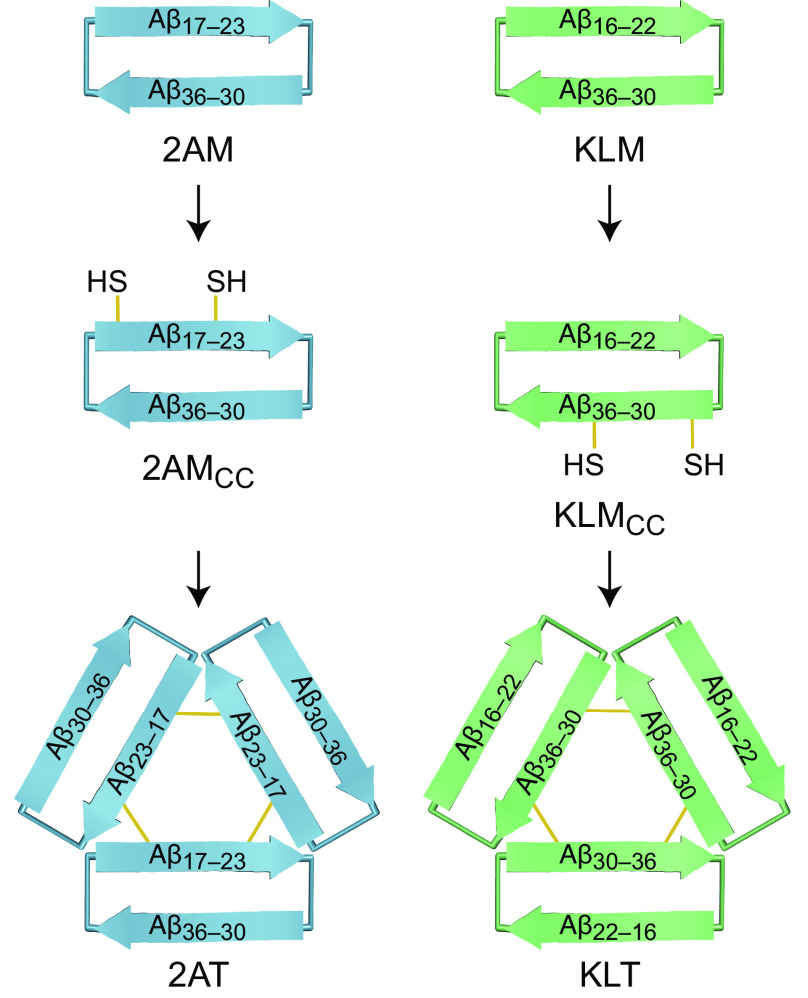
Design of triangular trimers 2AT and KLT from the β-hairpin peptide monomers 2AM and KLM. *SI Appendix*, Fig. S2 shows the chemical structure of each compound.

This paper reports the structural, biophysical, and biological characterization of the Aβ oligomer models 2AT and KLT, which are covalently stabilized isomorphic triangular trimers. 2AT is composed of β-hairpin peptides that mimic an Aβ_17–36_ β-hairpin, while KLT is composed of β-hairpin peptides that mimic an Aβ_16–36_ β-hairpin (Fig. 1). Although 2AT and KLT appear morphologically similar, they differ in the arrangement of their component β-hairpin peptides: in 2AT, Aβ_30–36_ constitutes the three outer β-strands of the trimer; in KLT, Aβ_30–36_ constitutes the three inner β-strands of the trimer. Furthermore, the surfaces of 2AT and KLT differ markedly, because of the different arrangement and β-strand alignments of the component β-hairpin peptides. The studies described below show that 2AT and KLT also exhibit markedly different biophysical and biological properties and may thus reflect the types of differences that occur among oligomers of full-length Aβ.

## Results and Discussion

### Aβ β-Hairpin Peptides 2AM and KLM.

We previously reported the design and study of the Aβ β-hairpin peptides 2AM and KLM, which mimic Aβ β-hairpins with different β-strand alignments (87, 92). 2AM mimics an Aβ β-hairpin in which an Aβ_17–23_ β-strand is across from an Aβ_30–36_ β-strand (*SI Appendix*, Fig. S3*A*) similar to the Aβ_17–36_ β-hairpin elucidated by Härd et al. (*SI Appendix*, Fig. S1); KLM mimics an Aβ β-hairpin in which an Aβ_16–22_ β-strand is across from an Aβ_30–36_ β-strand (*SI Appendix*, Fig. S3*C*) similar to the Aβ_16–36_ β-hairpin reported by Tycko et al. (*SI Appendix*, Fig. S1). While 2AM and KLM are designed to mimic the Aβ_17–36_ and Aβ_16–36_ β-hairpins elucidated by Härd et al. and Tycko et al., 2AM and KLM could also serve as models for β-hairpins formed by p3, an amyloid precursor protein cleavage product composed of Aβ residues 17 to 40 known to form oligomers (93).

KLM is an analogue of 2AM in which the top β-strand has shifted by one residue. A comparison of *SI Appendix*, Figs. S3 *B* and *D* illustrates the consequences of shifting the β-strand alignment by one residue. In 2AM, L_17_ pairs with V_36_ and occupies the same surface as V_36_. In KLM, K_16_ is added to the top strand and now pairs with V_36_, and L_17_ now occupies the opposite surface of V_36_ and pairs with M_35_. The β-strands of 2AM and KLM are linked together using the β-turn mimic δ-amine-linked ornithine (94). 2AM and KLM also contain *N*-methyl groups to limit uncontrolled aggregation: in 2AM, the *N*-methyl group is on F_20_; in KLM, the *N*-methyl group is on G_33_. 2AM contains an ornithine residue at position 35 instead of the native methionine. Ornithine is a charged isostere of methionine that helps solubilize the peptide. We have shown that a variant of 2AM with the native methionine at position 35 assembles in the crystal state in the same fashion as 2AM (87).

X-ray crystallography revealed that 2AM and KLM fold to form twisted β-hairpins that assemble to form different triangular trimers (*SI Appendix*, Figs. S3 *E* and  *F*). (For the X-ray crystallographic study of KLM, we used a variant of KLM with a para-iodo group on F_19_ (KLM_I_) to facilitate phase determination.) In the triangular trimer formed by 2AM, Aβ_30–36_ constitutes the three outer β-strands of the trimer, whereas in the triangular trimer formed by KLM_I_, Aβ_30–36_ constitutes the three inner β-strands of the trimer. The triangular trimers formed by 2AM and KLM_I_ each further assemble to form different dodecamers (*SI Appendix*, Fig. S4). The crystal structures of 2AM and KLM_I_ demonstrated that Aβ β-strand alignment within β-hairpin peptides can impact the structures of the oligomers that the peptides form. While the triangular trimers formed by 2AM and KLM_I_ appear similar in morphology, the differing β-strand alignments of their component peptides and the different ways the two peptides assemble to form trimers considerably impact the surfaces of the trimers, which in turn impact the higher-order assembly of the trimers to form dodecamers.

### Design and Synthesis of the Covalently Stabilized Triangular Trimers 2AT and KLT.

While 2AM and KLM assemble to form triangular trimers at the millimolar concentrations of X-ray crystallography, they do not appear to form triangular trimers at low micromolar concentrations, which are more biologically relevant (95). For this reason, covalent stabilization of the triangular trimers is needed to study their structural, biophysical, and biological properties outside of the crystal lattice. Covalent stabilization of the triangular trimers also ensures oligomer homogeneity, eliminating the monomer-oligomer equilibria that would occur for monomers that assemble to form trimers or other oligomers. We previously stabilized the triangular trimer formed by 2AM by installing disulfide bonds at the three corners of the trimer to create 2AT (96). This was achieved by mutating L_17_ and A_21_ of 2AM to cysteine to yield the peptide 2AM_CC_, which is then oxidized to form 2AT (Fig. 1 and *SI Appendix*, Fig. S2).

We have now designed and synthesized the covalently stabilized triangular trimer KLT, which mimics a triangular trimer formed by KLM (Fig. 1 and *SI Appendix*, Fig. S2). At the three corners of a triangular trimer formed by KLM, A_30_ of one monomer subunit is paired with L_34_ of an adjacent monomer subunit. To stabilize KLM into a triangular trimer and thus create KLT, we mutated A_30_ and L_34_ to cysteine to create KLM_CC_ and then oxidized KLM_CC_ in aqueous DMSO to introduce disulfide cross-links. LC/MS analysis of the crude oxidation reaction mixture reveals that KLM_CC_ forms four major species: KLT, two distinct bis-disulfide cross-linked dimers, and a monomer with an intramolecular disulfide bond (*SI Appendix*, Fig. S5). KLT is isolated from the crude reaction mixture using reverse-phase HPLC to yield 3 to 6 mg of KLT with >98% purity from the oxidation of ~30 mg of KLM_CC_.

### Crystal Structures of 2AT and KLT.

**Fig. 2.**
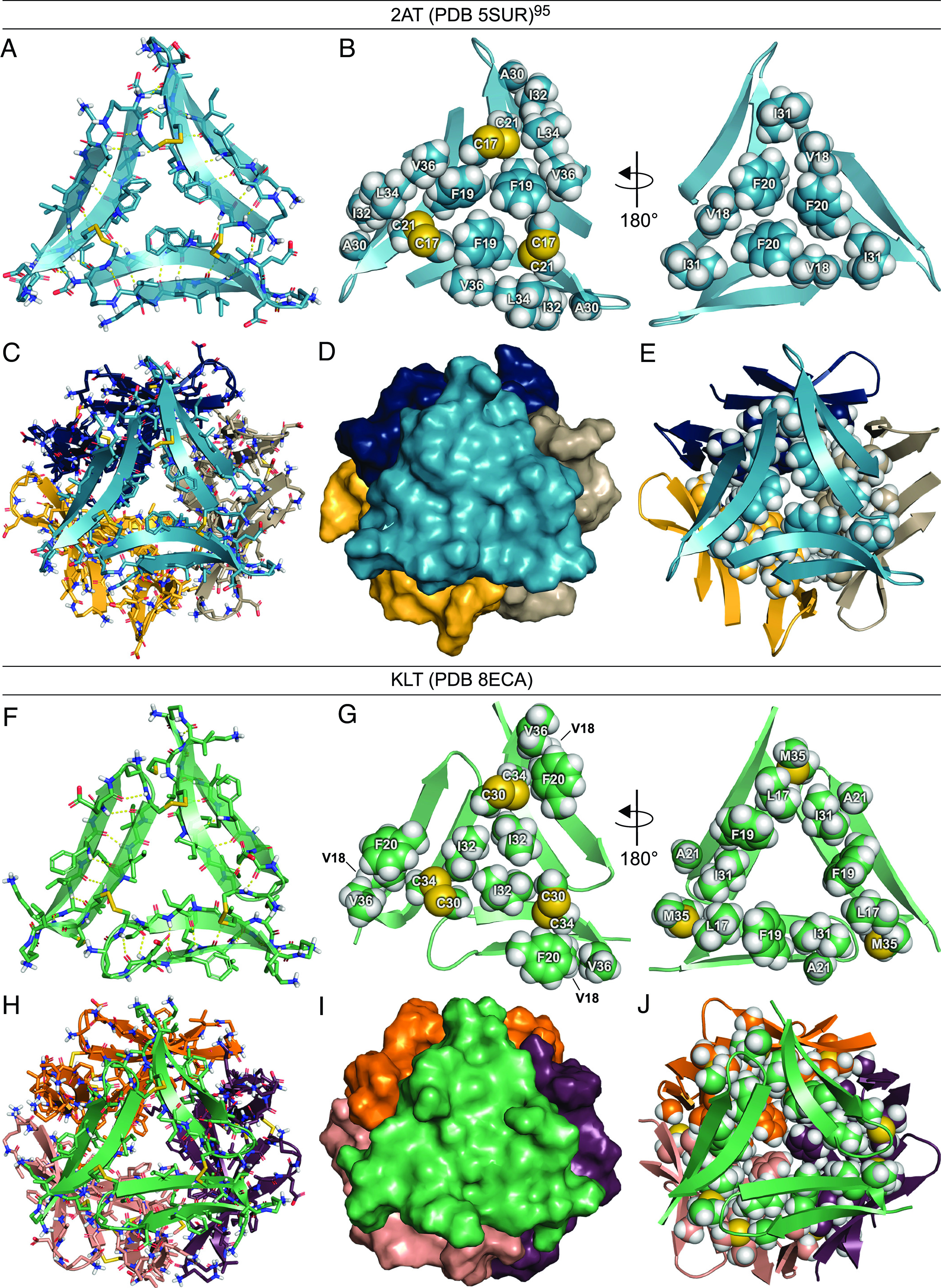
Crystal structures of 2AT and KLT (PDB 5SUR and 8ECA). (*A*) Cartoon and stick model of 2AT. (*B*) Cartoon and sphere models of 2AT illustrating the two hydrophobic surfaces. (*C*) Cartoon and stick model of the dodecamer formed by 2AT. (*D*) Surface model of the dodecamer formed by 2AT illustrating how the four trimers fit together. (*E*) Cartoon and sphere model of the dodecamer formed by 2AT illustrating the hydrophobic core formed by V_18_, F_20_, and I_31_. (*F*) Cartoon and stick model of KLT. (*G*) Cartoon and sphere models of KLT illustrating the two hydrophobic surfaces. (*H*) Cartoon and stick model of the dodecamer formed by KLT. (*I*) Surface model of the dodecamer formed by KLT illustrating how the four trimers fit together. (*J*) Cartoon and sphere model of the dodecamer formed by KLT illustrating the hydrophobic core formed by L_17_, F_19_, A_21_, I_31_, and M_35_.

To investigate the structures of 2AT and KLT, we turned to X-ray crystallography. We previously reported the crystal structure of 2AT (Fig. 2*A*, PDB 5SUR) (95) and now report the crystal structure of KLT (Fig. 2*F*, PDB 8ECA). The X-ray crystallographic phases for KLT were determined by first synthesizing a variant of KLT with a para-iodo group on F_19_ (KLT_I_) and using SAD phasing to determine the structure of KLT_I_ and then using the structure of KLT_I_ as a search model in molecular replacement. This approach succeeded in determining the phases for KLT because the crystal structure of KLT_I_ has the same space group and unit cell dimensions as the crystal structure of KLT. *SI Appendix*, Fig. S6 shows the asymmetric units of the crystal structures of KLT and KLT_I_, which both contain two copies of their respective trimers.

The crystal structures of 2AT and KLT show that the surfaces of both trimers are largely hydrophobic and differ markedly in the arrangement of residues displayed on each surface. A comparison of Fig. 2 *B* and  *G* best illustrates the differences between the surfaces of 2AT and KLT. Chief among these differences are the positions of F_19_ and F_20_ and I_31_ and I_32_. F_19_ and F_20_ occupy the central region of each surface of 2AT, and I_31_ and I_32_ occupy peripheral regions (Fig. 2*B*). I_31_ and I_32_ occupy central regions of KLT, and F_19_ and F_20_ occupy peripheral regions (Fig. 2*G*). Other amino acids shared by both trimers, such as V_18_ and V_36_, also occupy different positions on the surfaces of 2AT and KLT. The differences in the surfaces of 2AT and KLT result directly from the arrangement of the component β-hairpins of each trimer, as well as the alignment of the β-strands in the component β-hairpin peptides.

Hydrophobic packing between the surfaces of the trimers leads to higher-order assembly of 2AT and KLT in the crystal lattice. We previously reported that four copies of 2AT assemble to form a ball-shaped dodecamer (Fig. 2 *C*–*E*) (95). The crystal structure of KLT reveals that four copies of KLT also assemble to form a ball-shaped dodecamer (Fig. 2 *H*–*J*). Although the dodecamers formed by 2AT and KLT are nearly identical in morphology, they differ in topology, containing different residues on the insides and outsides of each dodecamer. The ball-shaped dodecamers formed by 2AT and KLT are both stabilized by a hydrophobic core at the center of each dodecamer. In the dodecamer formed by 2AT, V_18_, F_20_, and I_31_ fill the hydrophobic core, with a total of 48 amino acid side chains packing within the core (Fig. 2*E*). In the dodecamer formed by KLT, L_17_, F_19_, A_21_, I_31_, and M_35_ fill the hydrophobic core, with a total of 60 amino acid side chains packing within the core (Fig. 2*J*). The outer surfaces of each dodecamer are also largely hydrophobic and display different residues, with the 2AT dodecamer displaying F_19_, A_30_, I_32_, L_34_, V_36_, and the C_17_-C_21_ disulfide bonds and the KLT dodecamer displaying V_18_, F_20_, I_32_, V_36_, and the C_30_-C_34_ disulfide bonds.

Thus, the crystal structures of 2AT and KLT illustrate how the change in design from 2AT to KLT results in differences in structure between the trimers and supramolecular assembly of corresponding dodecamers. These structural differences arise from the differing β-strand alignments of the component peptides and the different orientations of the component peptides that are cross-linked together to form 2AT and KLT. The following sections detail the impacts that these structural differences have on the solution-phase assembly of 2AT and KLT; how 2AT and KLT interact with and affect cells; and the effect of 2AT and KLT on aggregation and biological properties of full-length Aβ_42_.

### Biophysical Studies of 2AT and KLT.

To investigate the solution-phase assembly and structural properties of 2AT and KLT, we used a suite of techniques including sodium dodecyl sulfate-polyacrylamide gel electrophoresis (SDS-PAGE), circular dichroism (CD) spectroscopy, native mass spectrometry, and mass photometry. At concentrations of 20 to 50 μM and pH 7 to 8, KLT is largely insoluble and forms large aggregates that pellet upon centrifugation, while 2AT is soluble and remains mostly in solution upon centrifugation. Despite the poor solubility of KLT, we performed the biophysical studies on each trimer without centrifugation before analysis.

**Fig. 3.**
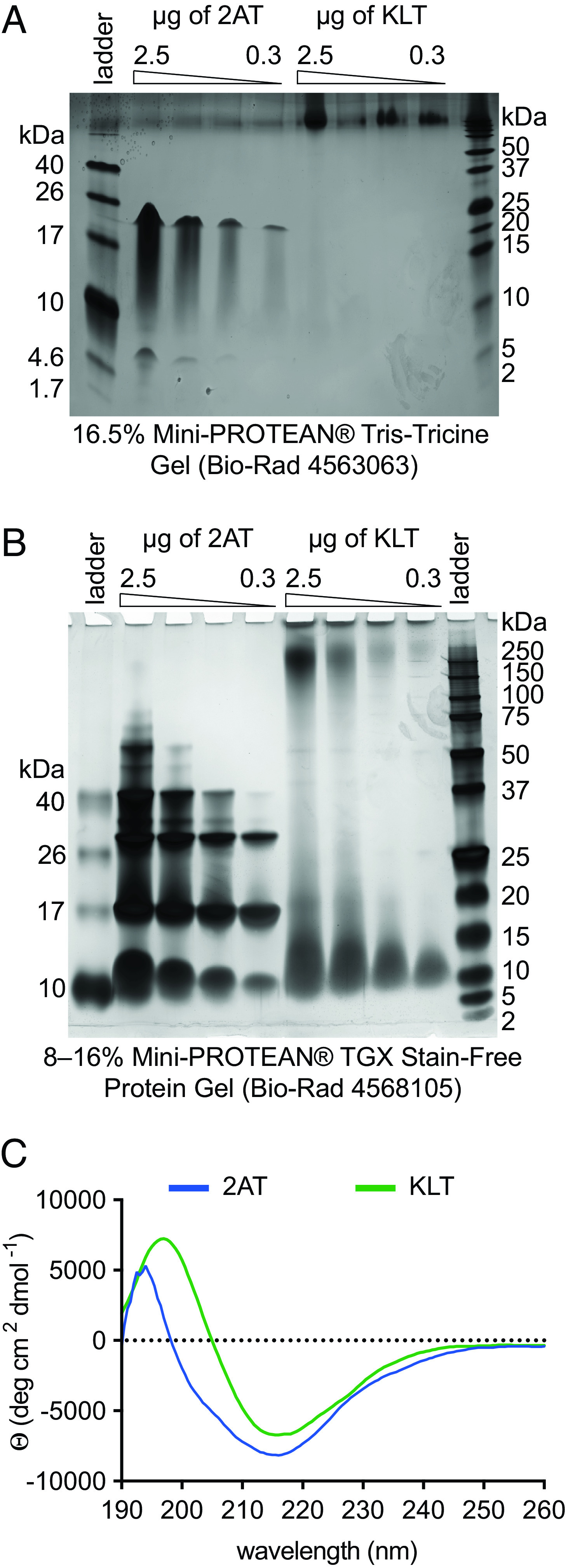
Biophysical studies of 2AT and KLT. (*A* and *B*) Silver-stained SDS-PAGE of 2AT and KLT. SDS-PAGE samples were prepared in 112.5 mM Tris buffer (pH 8.0) with 2% (w/v) SDS and 6% (v/v) glycerol and then run on a 16.5% polyacrylamide gel (*A*) or an 8–16% polyacrylamide gel (*B*). Both gels were run with Tricine buffer (100 mM Tris base, 100 mM Tricine, and 0.1% (w/v) SDS) at 30 V at 4 °C. (*C*) CD spectra of 2AT and KLT. CD spectra were acquired on 50 μM solutions of 2AT and KLT in 10 mM phosphate buffer (pH 7.4).

SDS-PAGE shows that in the lipid environment of SDS, 2AT and KLT exhibit markedly different assembly behavior. In a 16.5% polyacrylamide gel, 2AT migrates as two distinct bands: a band at a molecular weight consistent with a 21.2-kDa dodecamer and a band at a molecular weight consistent with a 5.3-kDa trimer (Fig. 3*A*). The putative dodecamer band formed by 2AT streaks downward, suggesting that the dodecamer is in equilibrium with lower-order oligomers, such as hexamers and nonamers. The streaks become fainter at lower concentrations, suggesting that formation of lower-order oligomers by 2AT is concentration dependent; however, this could also reflect the sensitivity of the silver stain. In contrast, KLT does not appear to significantly enter the 16.5% polyacrylamide gel, with most of the trimer entering the stacking gel and then accumulating at the top of the 16.5% running gel as large aggregates (Fig. 3*A*).

To further investigate 2AT and KLT in SDS-PAGE, we also ran the trimers on an 8 to 16% polyacrylamide gel. We found that 2AT exhibits different migration properties in the 8 to 16% polyacrylamide gel compared to the 16.5% polyacrylamide gel. In the 8 to 16% polyacrylamide gel, 2AT migrates as a series of bands corresponding to small oligomers ranging in size from 1 to 4 or more copies of 2AT, corresponding to trimer, hexamer, nonamer, and dodecamer and larger (Fig. 3*B*). The higher-order oligomer bands at and above the 40-kDa marker band become fainter at lower concentrations, suggesting that the formation of these higher-order oligomers is concentration dependent; however, this could also reflect the sensitivity of the silver stain. The migration differences observed for 2AT between the two different gels may arise from differences in the chemical composition among the two commercially available gels. KLT more substantially enters the 8 to 16% polyacrylamide gel than it does the 16.5% polyacrylamide gel, migrating as bands that correspond to a large aggregate composed of more than fifty copies of KLT and a molecular weight greater than 250 kDa, as well as a lower-molecular-weight band which we interpret to be hexamer or trimer (Fig. 3*B*).

We used CD spectroscopy to better understand the structures and folding of 2AT and KLT in solution. For CD, we prepared 50 μM solutions of 2AT and KLT in phosphate buffer (pH 7.4). The CD spectra of both 2AT and KLT exhibit typical β-hairpin character (97, 98, 99), with minima centered around 216 nm and maxima below 200 nm (Fig. 3*C*). These data support a structural model where the component peptides of 2AT and KLT fold to form β-hairpins in solution. These folded β-hairpins likely share significant structural similarity with the folded β-hairpins observed in the crystal structures of 2AT and KLT (Fig. 2 *A* and *F*).

We turned to native mass spectrometry to further investigate the assembly of 2AT and KLT. Native mass spectrometry has emerged as a powerful tool for studying protein and peptide complexes in the gas phase while preserving noncovalent interactions (100, 101). For these studies, we used an ultra-high mass range (UHMR) Orbitrap mass spectrometer and an ion mobility–mass spectrometry (IM-MS) instrument. In both instruments, 2AT and KLT solutions were introduced using nondisrupting nano-electrospray ionization (102, 103, 104). In UHMR, the high resolution and sensitivity of the orbitrap allow identification of multiple oligomers with multiple charge states. In cIMS, the gas-phase oligomers are separated by size, shape, and charge by applying a traveling wave electric field that carries the oligomers through a cyclic ion mobility cell (105). In both UHMR and cIMS, 2AT and KLT are prepared in a nondenaturing electrolyte solution compatible with mass spectrometry (200 mM ammonium acetate solution at pH 7.4).

**Fig. 4.**
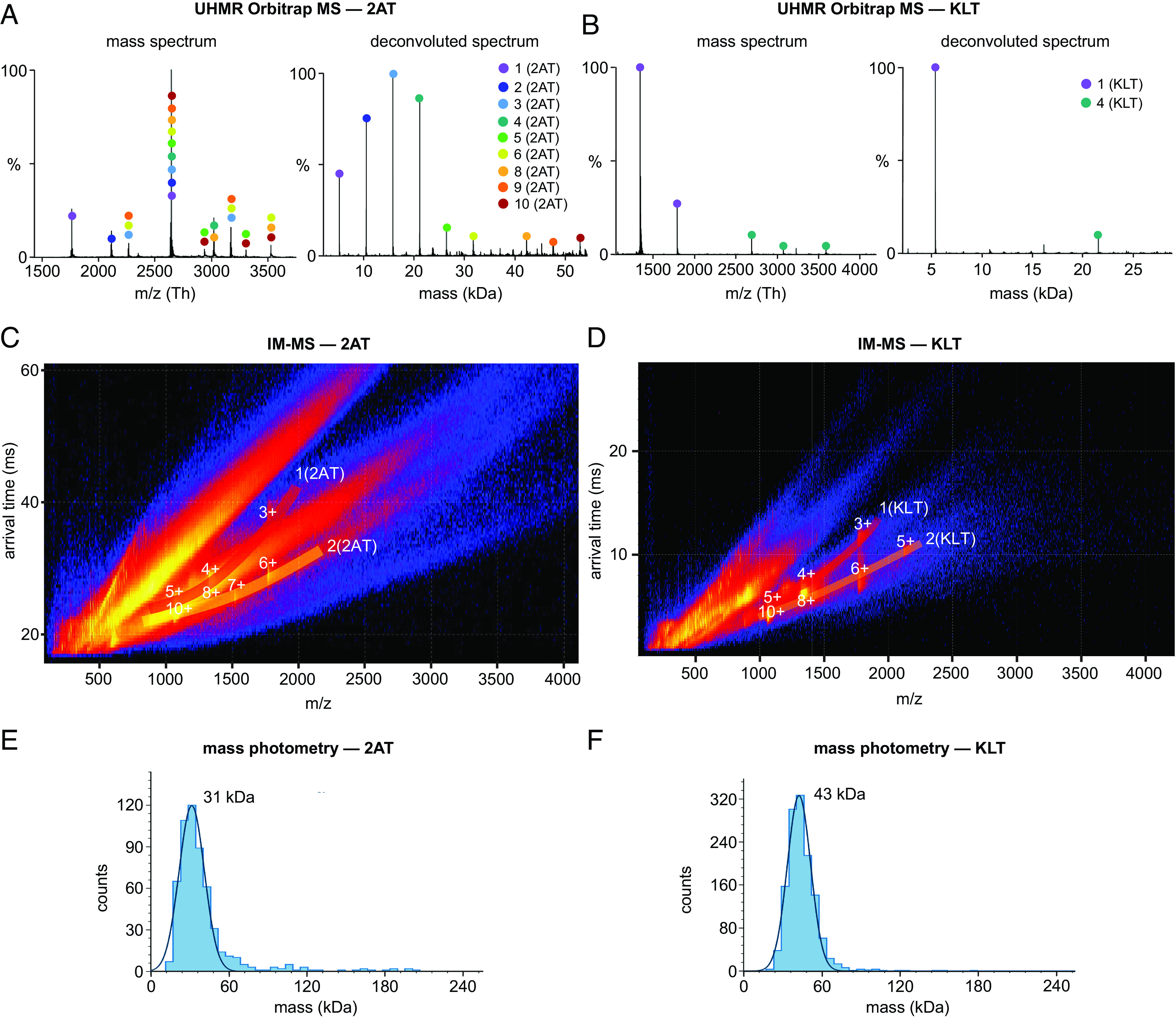
Mass spectrometric studies of 2AT and KLT. (*A* and *B*) Native mass spectra and deconvolved spectra of 2AT (*A*) and KLT (*B*). Samples were prepared at 40 μM in 200 mM ammonium acetate solution (pH 7.4) and analyzed immediately. (*C* and *D*) Native IM-MS mobiligrams of 2AT (*C*) and KLT (*D*). Samples were prepared at 40 μM in 200 mM ammonium acetate solution (pH 7.4) and incubated for 6 h at room temperature and then analyzed. Numeric labels indicate the charge state of the corresponding oligomeric species. (*E* and *F*) Mass photometry analysis of 2AT (*E*) and KLT (*F*). Samples were prepared at 0.65 μM in 200 mM ammonium acetate solution (pH 7.4) and analyzed immediately.

The UHMR results show that both 2AT and KLT exhibit different assembly characteristics under the conditions of this experiment. Assemblies of 2AT containing two, three, or four copies of the trimer (hexamers, nonamers, and dodecamers) are the predominant species observed in UHMR-MS, with minor species containing up to ten copies of the trimer also observed (Fig. 4*A*). In contrast, the predominant KLT species observed in UHMR-MS is unassembled trimer, with a minor assembly containing four copies of the trimer also observed (Fig. 4*B*). cIMS of 2AT and KLT shows that the two trimers exhibit similar assembly characteristics under the conditions of this experiment. The IM-MS mobiligrams for both 2AT and KLT show the unassembled trimers as well as assemblies containing two copies of the trimers (Fig. 4 *C* and *D*).

To gain additional insights into the solution-phase assembly of 2AT and KLT, we also used mass photometry. In mass photometry, the contrast of light (reflection and scattering) from individual oligomers in solution is used to measure oligomer sizes and obtain a size distribution (106). Molecular weights of the oligomers are estimated using protein standards analyzed on the mass photometry instrument. Mass photometry allows measurement of masses from 30 kDa to 5 MDa and is thus not sensitive to smaller species such as the 5.3 kDa trimers, 10.6 kDa hexamers, 15.9 kDa nonamers, and 21.2 kDa dodecamers. Mass photometry analysis of 2AT and KLT shows that both trimers assemble to form higher-order oligomers containing 6 to 8 copies of the trimer, with the predominant 2AT assembly observed having a molecular weight centered around 31 kDa and the predominant KLT assembly observed having a molecular weight centered around 43 kDa (Fig. 4 *E* and *F*).

The experiments described in the preceding paragraphs demonstrate that 2AT and KLT exhibit markedly different assembly characteristics in solution. 2AT forms a mixture of small soluble oligomers containing 2 to 8 copies of the trimer, while KLT generally forms large aggregates containing more than 50 copies of the trimer with molecular weights greater than 250 kDa. Furthermore, the solution-phase assemblies observed for both 2AT and KLT vary among the different biophysical techniques. While we do not fully understand what leads to the different assembly characteristics for 2AT and KLT, the assemblies observed for each trimer appear to depend on the conditions under which the experiment is performed as well as the biophysical technique used to observe the assemblies. In X-ray crystallography, cIMS, and mass photometry, 2AT and KLT exhibit similar assembly properties. However, 2AT and KLT exhibit contrasting assembly behaviors in SDS-PAGE and UHMR, suggesting that structural differences between the two trimers impact solution-phase assembly under the conditions of these techniques. Table 1 summarizes the structural and biophysical data for 2AT and KLT and highlights the multifarious assembly characteristics of the two trimers.

**Table 1.** Oligomer sizes observed for 2AT and KLT in the structural and biophysical studies

Trimer	X-ray crystallography	SDS-PAGE	UHMR	cIMS	Mass photometry
2AT	4	1, 2, 3, 4, and larger	1, 2, 3, 4, and larger	1 and 2	6–8
KLT	4	1–2 and >50	1 and 4	1 and 2	6–8

### Cell-Based Biological Studies of 2AT and KLT.

Exposing cells in culture to oligomers of full-length Aβ elicits a multitude of downstream cellular events that culminate in cell toxicity and cell death (*SI Appendix*, Fig. S8) (17, 20, 107). We sought to determine whether 2AT and KLT also exhibit toxicity toward cells and whether the differences in structure and solution-phase assembly between 2AT and KLT lead to differences in cellular toxicity. To investigate the toxicity of 2AT and KLT, we examined three toxicity markers—changes in ATP levels, cell viability, and activation of the apoptosis marker caspase-3/7—in SH-SY5Y cells exposed to 2AT or KLT. SH-SY5Y cells are a human neuroblastoma cell line commonly used in cell-based studies of Aβ oligomers (108, 109). For all three toxicity assays, we exposed the cells to varying concentrations of 2AT or KLT (50 to 0.2 μM) (110, 111, 112) or deionized water (vehicle) for 72 h and then performed the assays according to the manufacturer’s instructions.

**Fig. 5.**
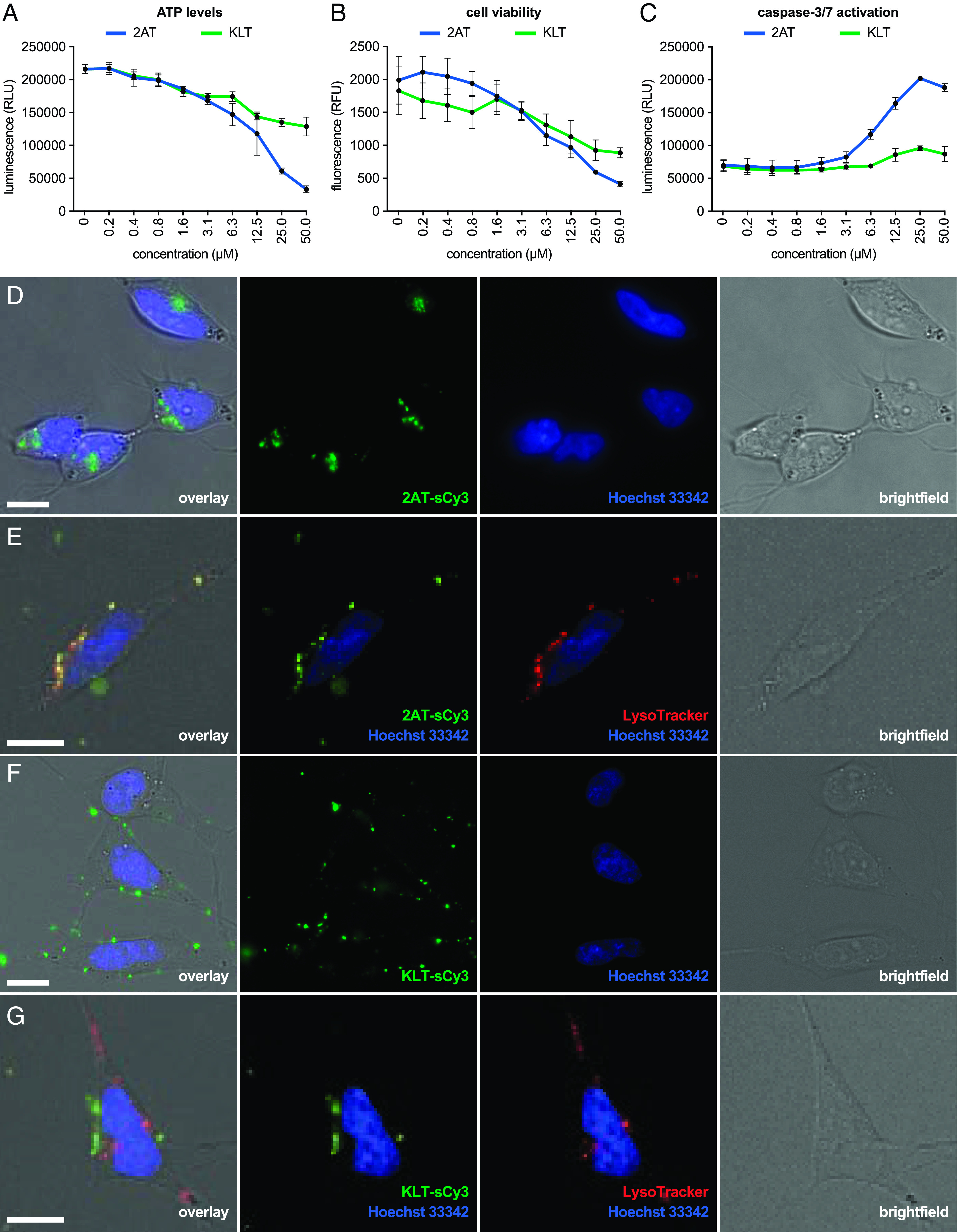
Cell-based biological studies of 2AT and KLT. (*A*) CellTiter-Glo ATP assay. (*B*) ApoLive-Glo cell viability assay. (*C*) ApoLive-Glo caspase-3/7 activation assay. The cell-based assays in *A*–*C* were performed by exposing SH-SY5Y cells to a twofold dilution series of 2AT or KLT for 72 h. The cell-based experiments were performed on 2AT and KLT side-by-side on the same plate of cells to ensure identical experimental conditions. All data from the assays are shown as the mean of three technical replicates, with error bars representing the SD. (*D*–*G*) Representative live-cell fluorescence and brightfield micrographs of SH-SY5Y cells after treatment with 1 μM 2AT-sCy3 (*D*), 1 μM 2AT-sCy3 and LysoTracker (*E*), 1 μM KLT-sCy3 (*F*), or 1 μM KLT-sCy3 and LysoTracker (*G*); (Scale bars, 10 μm).

The cell-based assays indicate that 2AT and KLT both exhibit toxicity toward SH-SY5Y cells, with 2AT showing greater toxicity than KLT. The assays also indicate that 2AT and KLT may elicit toxicity through different mechanisms. 2AT and KLT both induce a reduction in ATP levels at concentrations as low as 1.6 μM, but at concentrations above 12.5 μM, 2AT induces a twofold greater reduction in ATP levels (Fig. 5*A*). 2AT and KLT both reduce cell viability at concentrations as low as 12.5 μM, with 2AT exhibiting a greater effect on cell viability at concentrations above 12.5 μM (Fig. 5*B*). 2AT induces activation of caspase-3/7, eliciting an increase in caspase-3/7 activity at concentrations as low as 6.3 μM (Fig. 5*C*). In contrast, KLT induces little or no increase in caspase-3/7 activity at any of the concentrations tested. The cytotoxicity data suggest that 2AT elicits toxicity by activating apoptosis, which leads to a decrease in ATP levels and cell viability, whereas KLT does not appear to activate apoptosis, instead eliciting a decrease in ATP levels and cell viability through an alternative mechanism.

To further investigate the biological properties of 2AT and KLT, and to gain insights into why the two trimers appear to elicit toxicity through different mechanisms, we visualized how each trimer interacts with SH-SY5Y cells using fluorescence microscopy. We prepared analogues of 2AT and KLT singly labeled with the fluorophore sulfo-cyanine3 (2AT-sCy3 and KLT-sCy3) and then used live-cell fluorescence microscopy to visualize SH-SY5Y cells exposed to the labeled trimers. We have previously found that single labeling with sCy3 of a related triangular timer marginally impacts the assembly properties of the trimer (113). The microscopy studies reveal that 2AT-sCy3 and KLT-sCy3 interact with the cells differently. 2AT-sCy3 accumulates as punctate features inside the cells (Fig. 5*D*), whereas KLT-sCy3 does not appear to significantly enter the cells and instead accumulates as punctate features on the plasma membrane (Fig. 5*F*).

The punctate appearance and intracellular localization of 2AT-sCy3 suggest that 2AT-sCy3 enters the cells through an endocytic mechanism. To investigate whether 2AT-sCy3 enters the cells through endocytosis, we concurrently treated cells with 2AT-sCy3 and the fluorescent dye LysoTracker™ (ThermoFisher Scientific), which stains acidic late endosomes and lysosomes. Live-cell fluorescence microscopy of these cells reveals that 2AT-sCy3 colocalizes with LysoTracker, providing evidence that 2AT enters the cells through endocytosis (Fig. 5*E*). In contrast, live-cell fluorescence microscopy of cells treated KLT-sCy3 and LysoTracker reveals that KLT-sCy3 does not colocalize with LysoTracker (Fig. 5*G*).

The different ways that 2AT-sCy3 and KLT-sCy3 interact with SH-SY5Y cells are consistent with how different assemblies of full-length Aβ have been reported to interact with cells. 2AT appears to enter the cells through endocytic uptake and accumulates in intracellular endosomes. Endocytic uptake and intracellular accumulation of Aβ is an established pathway by which Aβ enters cells and forms aggregates (114, 115, 116), and some evidence suggests that intracellular accumulation of Aβ precedes plaque formation (117, 118, 119). KLT appears to accumulate on the cell membrane without significantly entering the cells. Some Aβ oligomers directly interact with the cell membrane and do not enter cells, instead accumulating on the membrane as punctate high-molecular-weight aggregates (120, 121).

Although we do not fully understand what leads to the differences in toxicity and cellular interactions between 2AT and KLT, we believe that these differences may arise from the structural and assembly differences between the two trimers. In the membrane-like environment of SDS-PAGE, 2AT forms smaller oligomers, whereas KLT forms higher-molecular-weight aggregates. These differences in assembly may help explain why 2AT-sCy3 appears to enter the cells through an endocytic pathway and KLT-sCy3 appears to aggregate on the exterior of the cells. The endocytic uptake of 2AT may also lead to activation of caspase-3/7-mediated apoptosis by 2AT.

### Effects of 2AT and KLT on Aβ_42_.

To better understand the relationship between the Aβ oligomer models 2AT and KLT and full-length Aβ, we studied the effects of 2AT and KLT on Aβ_42_ fibrillization, Aβ_42_ toxicity, and Aβ_42_ cellular interaction. In these studies, we found that 2AT and KLT affect Aβ_42_ differently, with KLT showing a greater effect in each experiment.

**Fig. 6.**
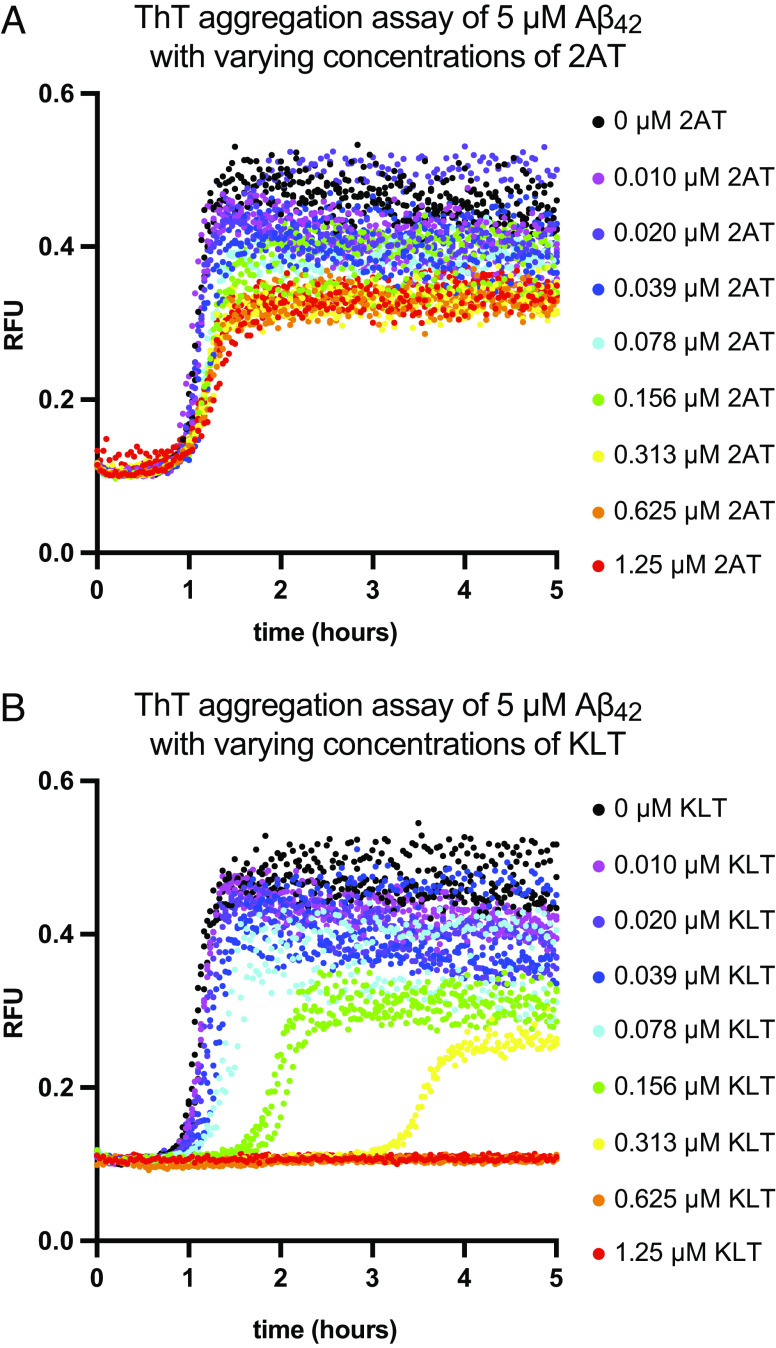
The effects of 2AT and KLT on Aβ_42_ fibrillization. ThT aggregation assays of 5 μM Aβ_42_ in the presence of varying concentrations of 2AT (*A*) or KLT (*B*) were performed at 25 °C under quiescent conditions in PBS (10 mM Na_2_HPO_4_, 1.8 mM KH_2_PO_4_, 137 mM NaCl, 2.7 mM KCl) at pH 7.4 containing 10 μM ThT. Fluorescence of ThT was monitored at 440 nm excitation and 485 nm emission.

To investigate the effect of 2AT and KLT on Aβ_42_ fibrillization, we performed thioflavin T (ThT) aggregation assays on 5 μM Aβ_42_ in the presence of varying concentrations of 2AT or KLT. In the ThT assays, KLT dramatically impacts Aβ_42_ fibrilization, completely suppressing fibrilization after 5 h at concentrations as low 0.625 μM (Fig. 6*B* and *SI Appendix*, Fig. S7*C*). At concentrations less than 0.625 μM, KLT increases the lag time of fibrilization and decreases end-point ThT fluorescence intensity in a dose-dependent manner. Even concentrations of KLT as low as 0.078 μM (0.016 molar equivalents) significantly increase the lag time. In contrast, concentrations of 2AT up to 1.25 μM have little or no effect on the lag time of fibrilization but moderately decrease end-point ThT fluorescence intensity (Fig. 6*A* and *SI Appendix*, Figs. S5*A* and S7*B*). These findings indicate that KLT interacts with full-length Aβ_42_ to a much greater extent than 2AT.

To investigate the effect of 2AT and KLT on Aβ_42_ toxicity, we performed a series of cell-based toxicity experiments in which we examined four toxicity markers—LDH release, changes in ATP levels, caspase-3/7 activation, and cell viability—in SH-SY5Y cells exposed to Aβ_42_ in the absence or presence of 2AT and KLT. We first identified an Aβ_42_ concentration that elicits significant toxicity in the absence of 2AT and KLT by exposing SH-SY5Y cells to varying concentrations of Aβ_42_ and then performing the toxicity assays. The toxicity markers establish that Aβ_42_ elicits significant toxicity at 6 μM (*SI Appendix*, Fig. S8). We next investigated the effect of 2AT and KLT on Aβ_42_ toxicity by exposing SH-SY5Y cells to 6 μM Aβ_42_ in the presence of a twofold dilution series of 2AT or KLT (12 to 0.09 μM) and then performing the toxicity assays.

**Fig. 7.**
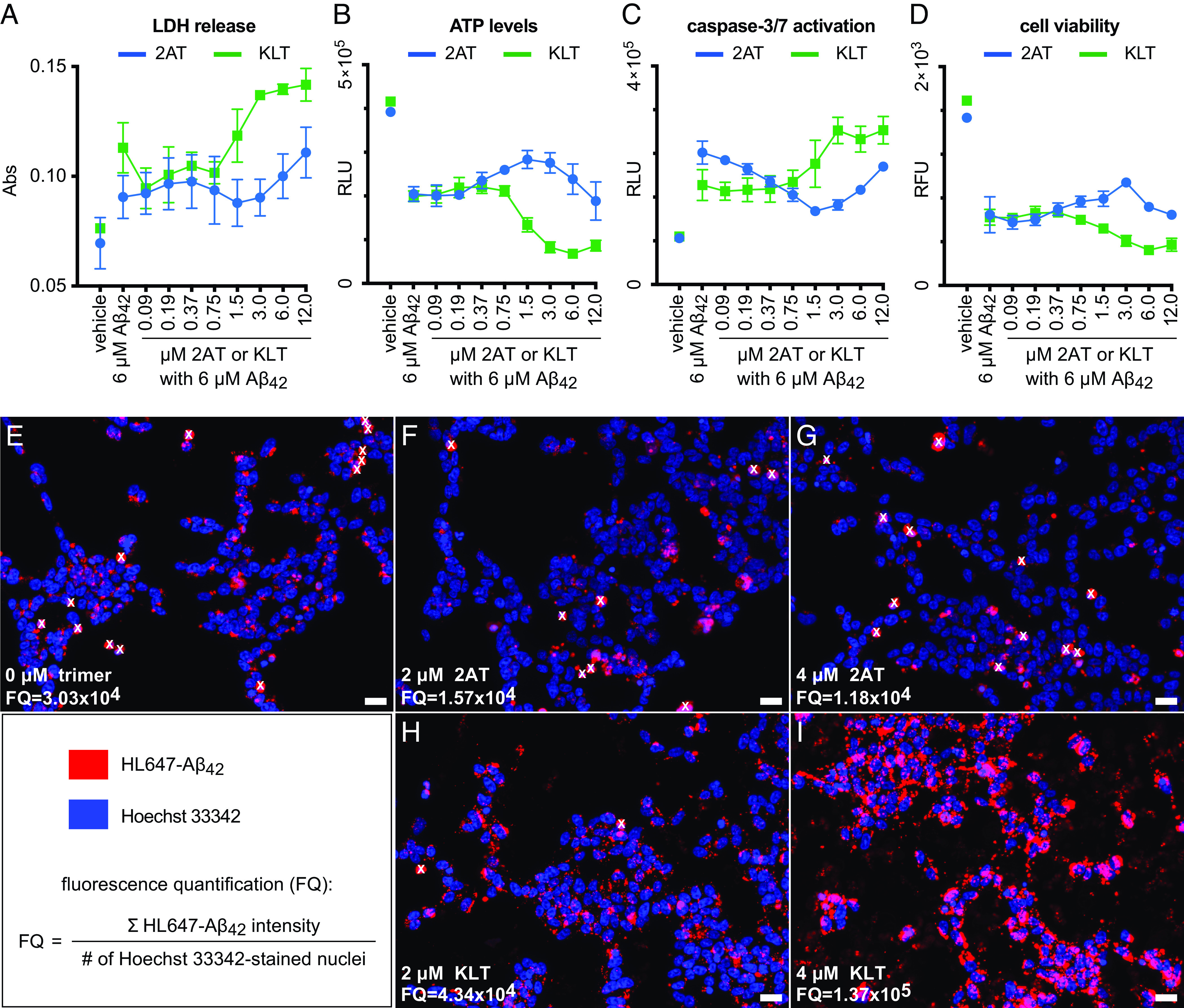
The effects of 2AT and KLT on Aβ_42_ toxicity and cellular interaction (*A*–*D*). (*A*) LDH release assay (*B*) CellTiter-Glo ATP assay. (*C*) ApoLive-Glo cell viability assay. (*D*) ApoLive-Glo caspase-3/7 activation assay. The cell-based assays in *A*–*D* were performed by exposing SH-SY5Y cells to 6 μM Aβ_42_ in the presence of a twofold dilution series of 2AT and KLT for 72 h. All data from the assays are shown as the mean of three technical replicates, with error bars representing the SD. (*E*–*I*) Live-cell fluorescence micrographs of SH-SY5Y cells after treatment with 1 μM HiLyte™ Fluor 647-Aβ_42_ (HL647-Aβ_42_) in the presence of 0 μM trimer (*E*) or 2 μM and 4 μM 2AT (*F* and *G*) or 2 μM and 4 μM KLT (*H* and *I*) for 16 h; (Scale bars, 20 μm.) Fluorescence quantification (FQ), as determined by (total fluorescence of HL647Aβ_42_)/(total number of cells) is indicated on each micrograph. Dead cells were excluded from the fluorescence quantification analysis and are marked with a white “x”.

2AT and KLT have opposite effects on Aβ_42_ toxicity. 2AT appears to moderately inhibit Aβ_42_ toxicity at concentrations between 0.75 μM and 3 μM, eliciting a slight decrease in LDH release and caspase-3/7 activation and a slight increase in ATP levels and cell viability at these concentrations relative to 6 μM Aβ_42_ alone (blue traces in Fig. 7 *A*–*D*). At 2AT concentrations greater than 3 μM, the inhibitory effect is diminished for all four toxicity markers, likely due to the toxic nature of 2AT at these higher concentrations (Fig. 5 *A*–*C*). In contrast, KLT appears to promote Aβ_42_ toxicity at concentrations greater than 0.75 μM, eliciting an increase in LDH release and caspase-3/7 activation and a decrease in ATP levels and cell viability relative to 6 μM Aβ_42_ alone (green traces in Fig. 7 *A*–*D*).

To further investigate the effects of 2AT and KLT on Aβ_42_ cell biology, and to gain insights into why the two trimers exhibit opposite effects on Aβ_42_ toxicity, we visualized the cellular interactions of fluorescently labeled Aβ_42_ in the absence and presence of 2AT and KLT. For these experiments, we exposed SH-SY5Y cells to 1 μM solutions of *N*-terminally labeled HiLyte™ Fluor 647-Aβ_42_ (HL647-Aβ_42_) containing 0 μM, 2 μM, or 4 μM 2AT or KLT and then used live-cell fluorescence microscopy to visualize and quantify HL647-Aβ_42_ fluorescence on the cells. In the absence of 2AT and KLT, HL647-Aβ_42_ accumulates as punctate features inside the cells (Fig. 7*E*), which is consistent with previous observations (116).

2AT and KLT appear to have opposite effects on the cellular interactions of Aβ_42_. In the presence of 2AT, HL647-Aβ_42_ exhibits reduced accumulation in the cells (Fig. 7 *F* and *G*), whereas in the presence of KLT, HL647-Aβ_42_ exhibits increased accumulation in and on the cells (Fig. 7 *H* and *I*). Quantification of the total fluorescence intensity from HL647-Aβ_42_ indicates that in the presence of 2 μM and 4 μM 2AT, there is approximately a twofold to threefold reduction in cellular HL647-Aβ_42_. In contrast, in the presence of 2 μM and 4 μM KLT, there is approximately a 1.5- to 14-fold increase in cellular HL647-Aβ_42_.

These fluorescence microscopy experiments shed light on why 2AT inhibits Aβ_42_ toxicity and KLT promotes Aβ_42_ toxicity. 2AT appears to inhibit the interaction of Aβ_42_ with cells and thus reduces the toxicity of Aβ_42_ toward the cells; KLT appears to promote the interaction of Aβ_42_ with cells and thus increases the toxicity of Aβ_42_ toward the cells. Although we do not yet understand why 2AT and KLT affect the cellular interaction and toxicity of Aβ_42_ differently, we speculate that 2AT may prevent Aβ_42_ toxicity by binding and sequestering Aβ_42_ monomers or oligomers, whereas KLT may promote Aβ_42_ toxicity by interacting with the cell membranes and recruiting Aβ_42_ to the cell membranes.

## Summary and Conclusion

The structural, biophysical, and biological studies of 2AT and KLT provide evidence that both trimers share biophysical and biological characteristics with oligomers of full-length Aβ, which thus suggests that 2AT and KLT are suitable models for Aβ oligomers. In X-ray crystallography, 2AT and KLT assemble to form dodecamers in the crystal lattice. In the variety of conditions used for the biophysical studies, 2AT forms small soluble oligomers ranging from hexamers, nonamers, and dodecamers and larger, and KLT forms high-molecular-weight aggregates. Aβ oligomers of these sizes have been observed in protein extracts from mouse and human brains and are thought to be important in Alzheimer’s disease (17, 19, 20).

Recently, the identification, characterization, and study of the putative Aβ dodecamer Aβ*56 has been called into question (27, 28). While we do not know whether Aβ*56 is real or an artifact, the formation of crystallographic dodecamers by the constrained Aβ β-hairpin peptides 2AM and KLM demonstrates that peptides derived from Aβ have a propensity to form dodecamers. The further observation that the covalently linked trimers 2AT and KLT are easy to prepare from monomers and both form crystallographic dodecamers (Fig. 2) and that 2AT forms dodecamers in SDS-PAGE (Fig. 3*A*) lends further credence to the idea that dodecamers are inherently stable structures.

Like oligomers of full-length Aβ, trimers 2AT and KLT are toxic toward cells in culture. The toxicity studies suggest that both trimers elicit toxicity by interacting with the cells and decreasing cell viability and ATP levels. The activation of caspase-3/7-mediated apoptosis by only 2AT suggests that 2AT interacts with and affects cells differently than KLT. The variation in apoptosis activation between 2AT and KLT demonstrates that different Aβ oligomer models have different biological properties, which may help explain why different oligomers of full-length Aβ exhibit different biological properties. In further support that 2AT and KLT elicit toxicity through different mechanisms, fluorescence microscopy demonstrates that the two trimers interact with cells differently. The differing cellular interactions of 2AT and KLT may help explain why some Aβ oligomers enter cells and other higher-molecular-weight oligomers accumulate on cell membranes.

The effects of 2AT and KLT on Aβ_42_ aggregation, toxicity, and cellular interaction establish that the trimers interact with full-length Aβ_42_. KLT appears to more readily interact with Aβ_42_, considerably inhibiting Aβ_42_ fibrillization at substoichiometric concentrations and promoting Aβ_42_ toxicity and cell interactions. In contrast, 2AT appears to interact less strongly with Aβ_42_, exhibiting a moderate effect on Aβ_42_ fibrillization and a modest inhibitory effect on Aβ_42_ toxicity and cell interactions. These different behaviors likely arise from the different structural and assembly properties of 2AT and KLT. These findings suggest a model in which oligomers of full-length Aβ composed of β-hairpins with different β-strand alignments differentially impact the aggregation, toxicity, and cellular interaction of other Aβ species.

Aβ oligomers that are prepared in vitro or isolated from tissue consist of a variety of different sizes and exhibit varying toxicities (17, 19, 20). The assembly and toxicity characteristics that researchers observe for a particular Aβ oligomer or mixture of Aβ oligomers appear to depend on the conditions under which the oligomers are prepared or isolated (20). The dizzying array of techniques reported for Aβ oligomer preparation and isolation has painted a complex picture of Aβ oligomers and has led to conflicting results and viewpoints about the significance of Aβ oligomers in Alzheimer’s disease. The varying assembly and biological characteristics exhibited by 2AT and KLT may help shed light on the disparate behaviors exhibited by different preparations or isolations of Aβ oligomers.

The study of 2AT and KLT support a model for the assembly of full-length Aβ in which Aβ folds to form β-hairpins with different β-strand alignments, which in turn assemble to form different sizes of oligomers with different biological properties. Some β-strand alignments lead to lower-molecular-weight oligomers that enter the cells and activate caspase-3/7-mediated apoptosis, which leads to a reduction in ATP levels and cell viability and eventual cell death. Other β-strand alignments lead to higher molecular weight aggregates that accumulate on the plasma membrane, which leads to an apoptosis-independent reduction in ATP levels and cell viability and eventual cell death. We recognize that our peptides and oligomers are chemical models and should thus be interpreted cautiously. While we do not yet know whether our trimer and dodecamer structures mimic the actual structures of Aβ oligomers formed in vitro or in the brain, we are actively attempting to address this question in our laboratory.

## Materials and Methods

Detailed methods are provided in the Supporting Information. KLM_CC_ was synthesized using standard Fmoc-based solid-phase peptide synthesis. KLT was synthesized by oxidizing KLM_CC_ in 20% aqueous DMSO for 48 hours. X-ray diffraction data for KLT and KLT_I_ were collected on a Rigaku Micromax-007HF X-ray diffractometer with a rotating copper anode, and the X-ray crystallographic phases were determined using molecular replacement and SAD phasing. SDS-PAGE was performed using 16.5% Mini-PROTEAN® Tris-Tricine Gels and an 8 to 16% Mini-PROTEAN® TGX Stain-Free Protein Gels. Silver staining was performed as previously described (122). CD spectroscopy was performed on a Jasco J-810 CD spectropolarimeter. UHMR-MS, IM-MS, and mass photometry were performed on a Thermo Q Exactive UHMR Orbitrap MS, Waters SELECT SERIES Cyclic Ion Mobility Spectrometry Q-cIMS-TOF system, and a TwoMP mass photometer. Cellular toxicity assays were performed on SH-SY5Y cells using the CyQUANT™ LDH Cytotoxicity Assay, the CellTiter-Glo® 2.0 Cell Viability Assay, and the ApoLive-Glo™ Multiplex Assay. 2AT and KLT were labeled with sulfo-cyanine3 by treating the trimers with sulfo-cyanine3 NHS ester. Live-cell fluorescence microscopy was performed on SH-SY5Y cells adhered to an Ibidi µ-Slide eight Well Chamber Slide. The cells were imaged using a Keyence BZ-X810 fluorescence microscope and analyzed using the Hybrid Cell Count application in the BZ-X810 Analyzer software. ThT assays were performed on 5 µM Aβ_42_ in PBS at pH 7.4 containing 10 µM ThT in the presence of a dilution series of 2AT or KLT (10 to 0.01 μM, 0 μM) in Corning® 96-well Half Area Black/Clear Flat Bottom Polystyrene NBS Microplates.

## Supplementary Material

Appendix 01 (PDF)

## Data Availability

All data are provided in the manuscript and supporting information. Coordinates for the X-ray crystallographic structures of KLT and KLT_I_ are deposited in the RCSB with PDB accession codes 8ECA (123) and 8EC9 (124). All study data are included in the article and/or *SI Appendix*.
