# Supplementary material for: Probing differences among Aβ oligomers with two triangular trimers derived from Aβ
Source: Proc Natl Acad Sci U S A. 2023 May 22;120(22):e2219216120. doi: 10.1073/pnas.2219216120 (PMC10235986; doi:10.1073/pnas.2219216120)

Supporting Information for
Probing Differences Among Aβ Oligomers with Two Triangular Trimers Derived from Aβ

Adam G. Kreutzer,[a†*] Gretchen Guaglianone,[a†] Stan Yoo,[a] Chelsea Marie T. Parrocha,[b] Sarah M. Ruttenberg,[a] Ryan J. Malonis,[c] Karen Tong,[c] Yu-Fu Lin,[d] Jennifer T. Nguyen,[b] William J. Howitz,[a] Michelle N. Diab,[a] Imane L. Hamza,[a] Jonathan R. Lai,[c] Vicki H. Wysocki,[d] and James S. Nowick[a,b*]

[a]Department of Chemistry, University of California Irvine, Irvine, CA 92697
[b]Department of Pharmaceutical Sciences, University of California Irvine, Irvine, CA 92697
[c]Department of Biochemistry, Albert Einstein College of Medicine, Bronx, NY 10461
[d]Resource for Native Mass Spectrometry Guided Structural Biology, The Ohio State University, Columbus, OH 43210

[†]These authors contributed equally to this work.

*Corresponding authors:
Adam G. Kreutzer, 4302 Natural Sciences 1, University of California Irvine, Irvine, CA 92697
James S. Nowick, 4126 Natural Sciences 1, University of California Irvine, Irvine, CA 92697

Email: akreutze@uci.edu and jsnowick@uci.edu

This PDF includes:

Supporting Figures and Table

Figure S1. Cartoons of β-hairpins illustrating different alignments of the β-strands from the central and *C*-terminal regions of Aβ. S4

Figure S2. Chemical structures of 2AM, $2AM_{CC}$, 2AT, KLM, KLM_{CC}, and KLT. S5

Figure S3. β-Hairpin peptides 2AM and KLM. S6

Figure S4. X-ray crystallographic structures of the dodecamers formed by 2AM and KLM_I. S7

Figure S5. LC/MS analysis of the crude reaction mixture formed by the oxidation of KLM_{CC}. S8

Figure S6. Asymmetric units for the X-ray crystallographic structures of KLT and KLT_I. S9

Figure S7. Extended ThT aggregation assay data. S10

Figure S8. $Aβ_{42}$ is toxic toward SH-SY5Y cells. S11

Table S1. Crystallographic properties, crystallization conditions, and data collection and model refinement statistics for KLT and KLT_I. S12

Materials and Methods

General information. S13

Synthesis and purification of KLMcc. S13

Loading of the resin. S13

Peptide coupling. S13

Cleavage of the peptide from the resin. S14

Cyclization of the linear peptide. S14

Global deprotection of the cyclic peptide. S14

Reverse-phase HPLC purification. S15

Synthesis, purification, and characterization of KLT. S15

Synthesis of KLT. S15

LC/MS analysis of the KLM_{CC} oxidation reaction mixture. S15

Purification of KLT. S16

LC/MS characterization of KLT. S17

X-ray crystallography of KLT and KLT_I. S18

Crystallization procedure for KLT and KLT_I. S18

X-ray crystallographic data collection, data processing, and structure determination for KLT and KLT_I. S19

SDS-PAGE and silver staining. S20

SDS-PAGE. S20

Sample preparation and gel running. S20

Silver staining. S20

Circular dichroism spectroscopy. S21

Ultra-high mass range (UHMR) Orbitrap MS. S21

Ion mobility MS (IM-MS). S22

Mass photometry. S22

Cell-based toxicity assays of 2AT and KLT in SH-SY5Y cells. S23

Preparation of SH-SY5Y cells for the toxicity assays. S24

Preparation of 2AT and KLT for toxicity assays. S24

Treatment of the SH-SY5Y cells with 2AT and KLT. S25

CellTiter-Glo® 2.0 Cell Viability Assay. S25

ApoLive-Glo™ Multiplex Assay. S26

Preparation of 2AT-sCy3 and KLT-sCy3. S26

Live-cell fluorescence microscopy of SH-SY5Y cells treated with 2AT-sCy3 and KLT-sCy3. S28

Preparation of $A\beta_{42}$ for the ThT aggregation assays and cell-based toxicity assays. S29

ThT aggregation assay of Aβ_{42} in the presence of 2AT or KLT. S29
Preparation of 11 μM ThT in PBS. S29
Preparation of 2AT and KLT for the ThT assay. S30
Adding Aβ_{42} to the ThT assay plate. S31
Reading the ThT assay plate. S32
Cell-based toxicity assays of Aβ_{42} in the absence or presence of 2AT and KLT in SH-SY5Y cells. S33
Preparation of SH-SY5Y cells for the toxicity assays. S34
Preparation of Aβ_{42} in the absence or presence of 2AT and KLT for toxicity assays. S34
Treatment of the SH-SY5Y cells with Aβ_{42} with or without 2AT and KLT. S36
CyQUANT™ LDH Cytotoxicity Assay. S36
CellTiter-Glo® 2.0 Cell Viability Assay. S36
ApoLive-Glo™ Multiplex Assay. S37
Establishment of a concentration of Aβ_{42} that elicits significant toxicity. S37
Live-cell fluorescence microscopy of SH-SY5Y cells treated with HiLyte™ Fluor 647-Aβ_{42} in the absence or presence of 2AT or KLT. S38
Preparation of SH-SY5Y cells for live-cell fluorescence microscopy. S38
Preparation of HiLyte™ Fluor 647-Aβ_{42} in the absence or presence of 2AT and KLT for live-cell fluorescence microscopy. S38
Treatment of the SH-SY5Y cells with HiLyte™ Fluor 647-Aβ_{42} with or without 2AT and KLT. S39

References and Notes S41

Characterization Data

Characterization of 2AT. S43
Characterization of KLT. S46
Characterization of 2AT-sCy3. S49
Characterization of KLT-sCy3. S52

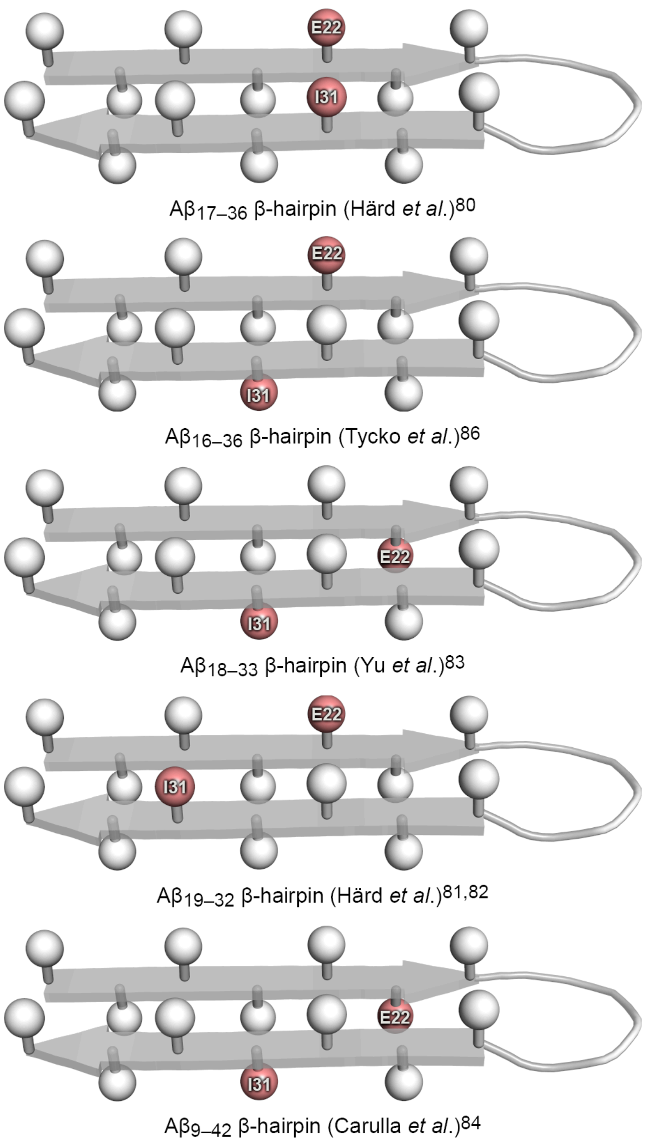

Figure S1. Cartoons of β-hairpins illustrating different alignments of the β-strands from the central and *C*-terminal regions of Aβ. Two residues (E_{22} and I_{31}) are highlighted in red to help illustrate the differences in residue pairings and variations in the surfaces among these Aβ β-hairpins. The structures of these β-hairpins were elucidated using either $A\beta_{40}$ or $A\beta_{42}$, and the residue range listed designates the Aβ residues that participate in β-hairpin formation.

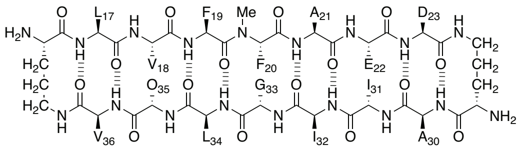

2AM
exact mass: 1744.0287
molecular weight as the TFA salt: 2218.9226

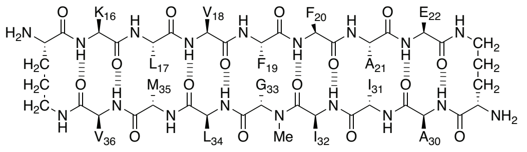

KLM
exact mass: 1774.0579
molecular weight as the TFA salt: 2249.0783

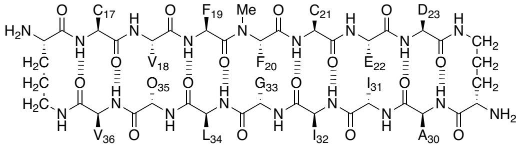

2AM$_{CC}$
exact mass: 1765.9259
molecular weight as the TFA salt: 2240.9739

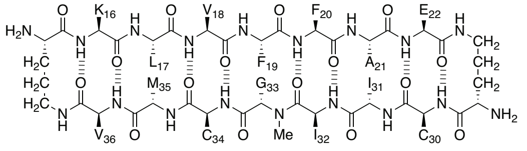

KLM$_{CC}$
exact mass: 1795.9551
molecular weight as the TFA salt: 2271.1296

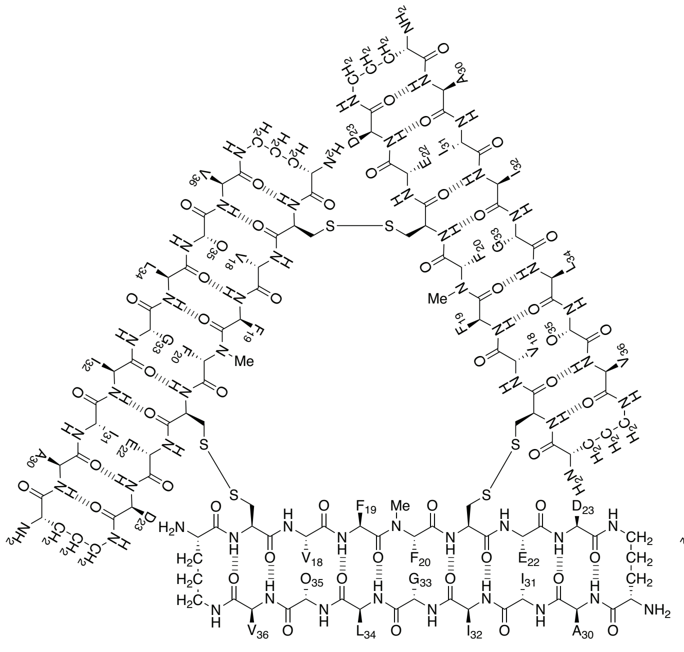

2AT
exact mass: 5291.7309
molecular weight as the TFA salt: 6716.8750

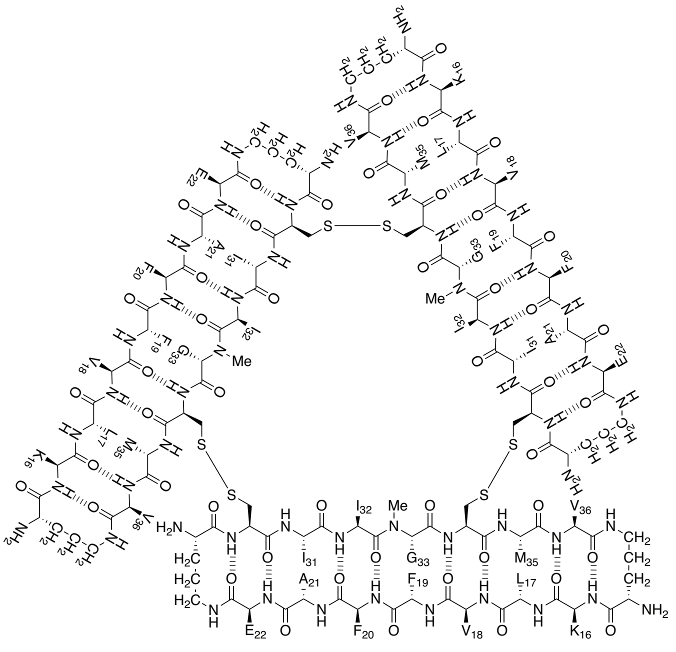

KLT
exact mass: 5381.8185
molecular weight as the TFA salt: 6807.3420

Figure S2. Chemical structures of 2AM, 2AM$_{CC}$, 2AT, KLM, KLM$_{CC}$, and KLT.

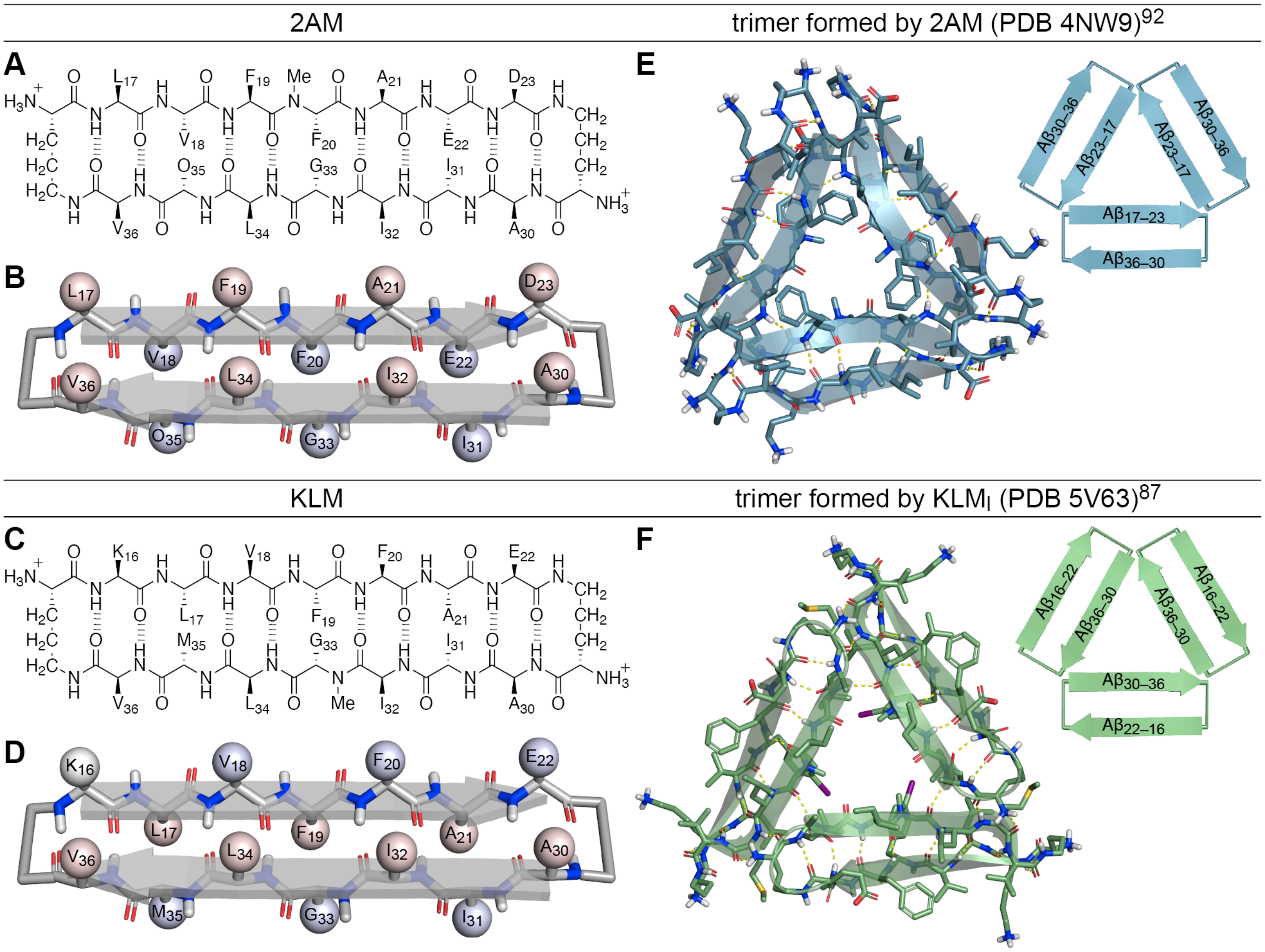

Figure S3. β-Hairpin peptides 2AM and KLM. ***(A)*** Chemical structure of 2AM, which mimics an $A\beta_{17-36}$ β-hairpin in which L_{17} pairs with V_{36}. ***(B)*** Three-dimensional cartoon representation of 2AM. ***(C)*** Chemical structure of KLM, which mimics an $A\beta_{16-36}$ β-hairpin in which K_{16} pairs with V_{36}. ***(D)*** Three-dimensional cartoon representation KLM. ***(E and F)*** Crystal structures of the trimers formed by 2AM (E) and a variant of KLM with a *para*-iodo group on F_{19} (KLM_I, F). The cartoon insets in E and F illustrate how 2AM and KLM_I assemble to form triangular trimers and the relationship of the structures to Aβ.

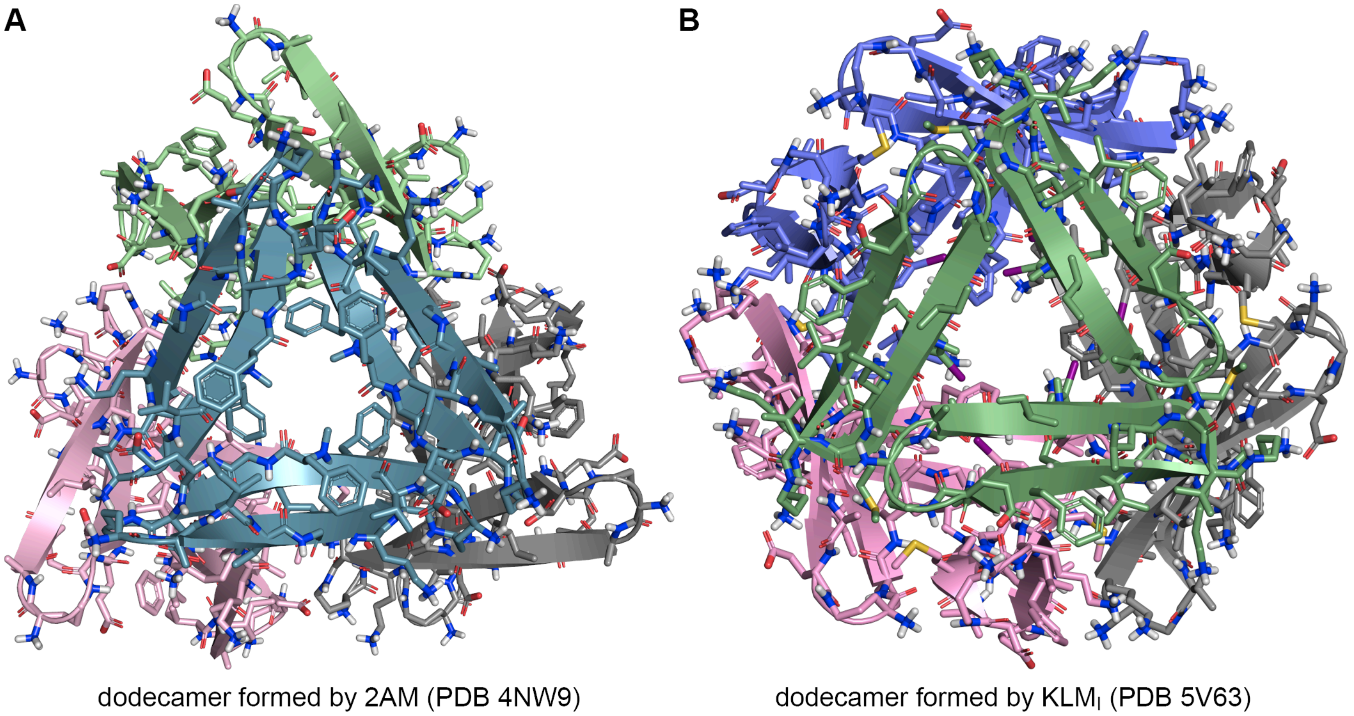

Figure S4. X-ray crystallographic structures of the dodecamers formed by 2AM (A) and KLM_I (B)

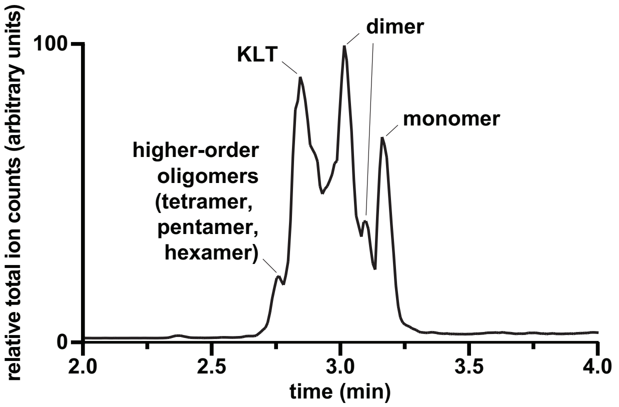

Figure S5. LC/MS analysis of the crude reaction mixture formed by the oxidation of KLM_{CC}. All species (KLT, monomer, dimers, and higher-order oligomers) contain disulfide linkages.

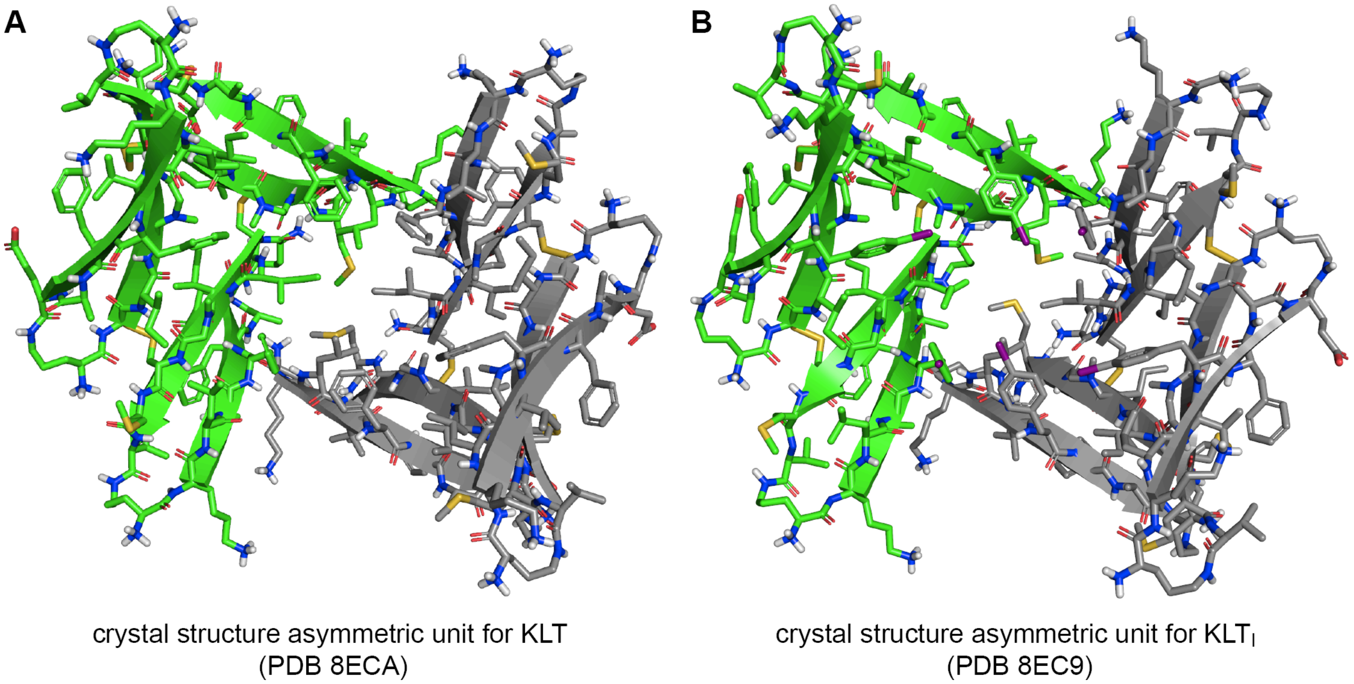

Figure S6. Asymmetric units for the X-ray crystallographic structures of KLT (A) and KLT$_I$ (B).

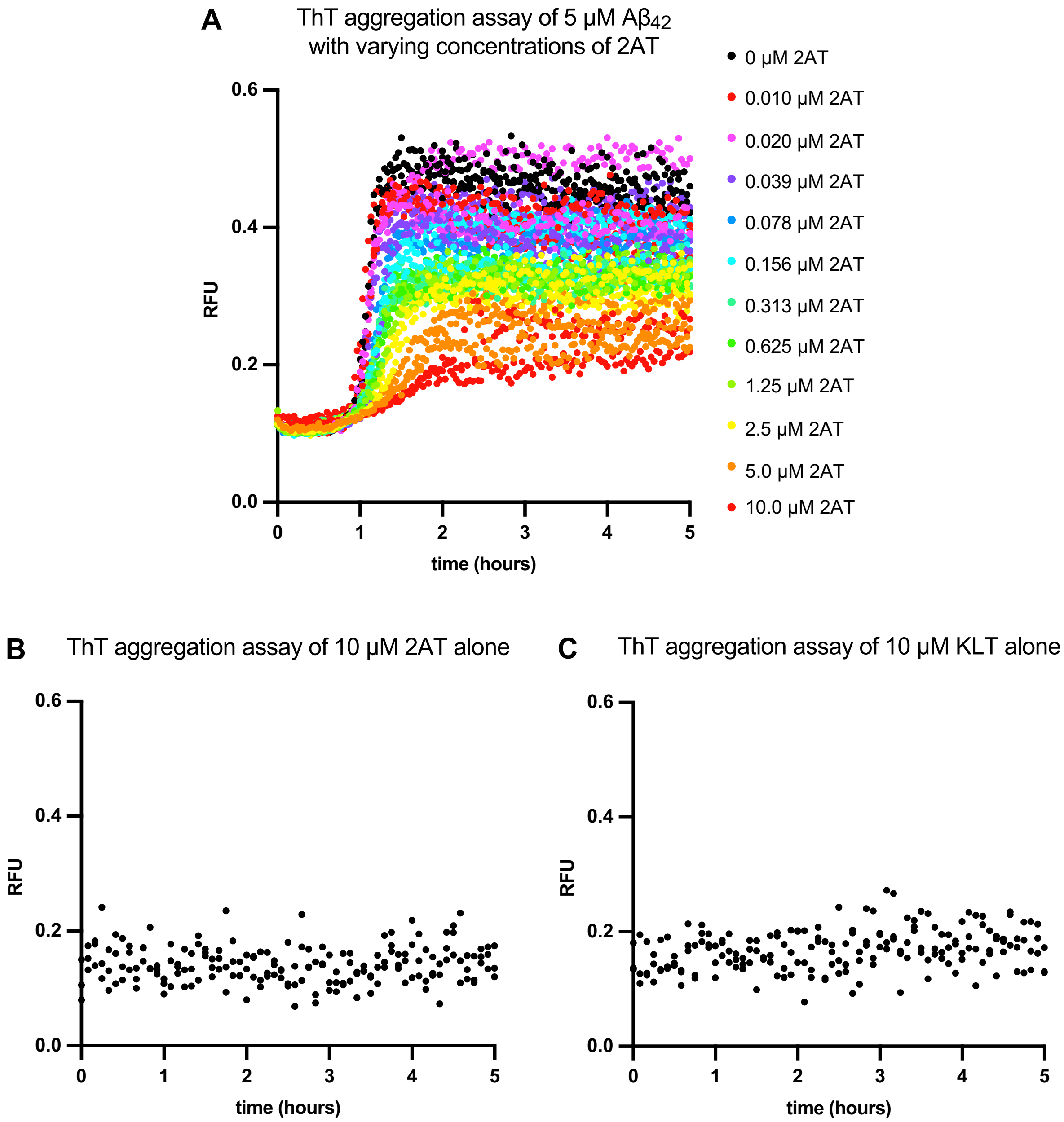

Figure S7. Extended ThT aggregation assay data. ***(A)*** 5 µM Aβ_{42} in the presence of varying concentrations of 2AT (10–0.01 µM). ***(B)*** 10 µM 2AT alone, demonstrating that 2AT does not interact with ThT. ***(C)*** 10 µM KLT alone, demonstrating that KLT does not interact with ThT. ThT assays were performed at 25 ºC under quiescent conditions in PBS (10 mM Na_2HPO_4, 1.8 mM KH_2PO_4, 137 mM NaCl, 2.7 mM KCl) at pH 7.4 containing 10 µM ThT. Fluorescence of ThT was monitored at 440 nm excitation and 485 nm emission.

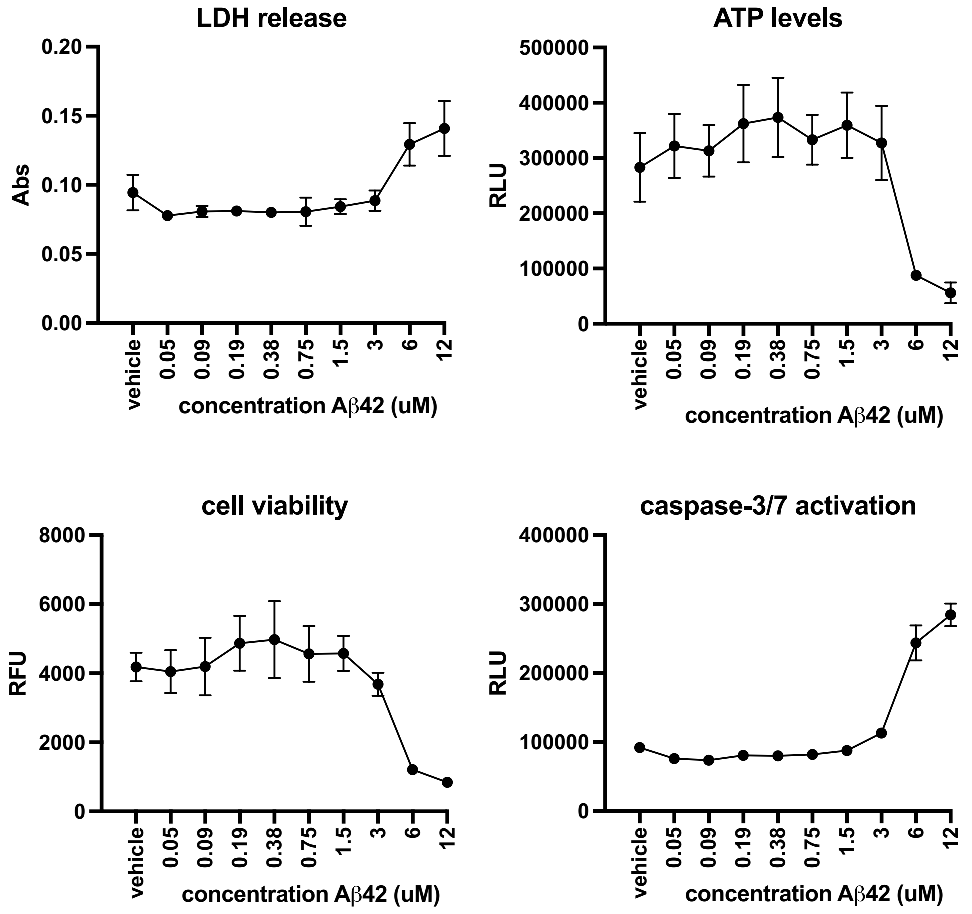

Figure S8. $A\beta_{42}$ is toxic toward SH-SY5Y cells. SH-SY5Y cells (30,000 cells/well on a black-walled half-area 96-well plate) were exposed to a twofold dilution series of $A\beta_{42}$ (12–0.05 μM) for 72 h. LDH release assays, CellTiter-Glo ATP assays, ApoLive-Glo caspase-3/7 activation assays, and ApoLive-Glo cell viability assays were then performed according to manufacturer's instructions. All data from the assays are shown as the mean of three technical replicates, with error bars representing the standard deviation.

Table S1. Crystallographic properties, crystallization conditions, and data collection and model refinement statistics for KLT and KLT_I.

compound	KLT	KLT_I
PDB ID	8ECA	8EC9
space group	$P3_121$	$P3_121$
a, *b*, *c* (Å)	43.53 43.53 93.32	43.06, 43.06, 92.76
α, β, λ (°)	90, 90, 120	90, 90, 120
molecules per asymmetric unit	6	6
wavelength (Å)	1.54	1.54
resolution (Å)	19.73–2.274 (2.355–2.274)	19.69–2.169 (2.247–2.169)
total reflections	211950 (8930)	11288 (1078)
unique reflections	5057 (489)	5653 (548)
multiplicity	41.9 (18.3)	34.6 (15.6)
completeness (%)	98.38 (87.14)	99.05 (92.21)
mean I/σ(I)	26.99 (1.16)	20.67 (1.27)
Wilson B factor	53.05	40.85
R_{merge}	0.7147 (2.059)	0.05607 (0.7596)
$R_{measure}$	0.7233 (2.111)	0.07929 (1.074)
$CC_{1/2}$	0.966 (0.447)	0.996 (0.512)
CC^*	0.991 (0.786)	0.999 (0.823)
R_{work}	0.2649 (0.4204)	0.2792 (0.4220)
R_{free}	0.3191 (0.4625)	0.3205 (0.4285)
number of non-hydrogen atoms	794	758
RMS_{bonds}	0.022	0.025
RMS_{angles}	1.15	1.47
Ramachandran favored (%)	94.44	83.33
Ramachandran allowed (%)	5.56	16.67
Ramachandran outliers (%)	0	0
rotamer outliers (%)	1.39	6.06
clash score	11.17	14.02
average B-factor	82.66	65.34
number of TLS groups	6	6
crystallization conditions	0.1 M Tris at pH 8.3, 0.2 M ammonium acetate, and 39% 2-methyl-2,4-pentanediol (MPD)	0.1 M Tris at pH 8.0, 0.2 M ammonium acetate, and 35% 2-methyl-2,4-pentanediol (MPD)

Materials and Methods[1]

General information

All chemicals were used as received unless otherwise noted. Methylene chloride (CH_2Cl_2) was passed through alumina under nitrogen prior to use. Anhydrous, amine-free *N,N*-dimethylformamide (DMF) was purchased from Alfa Aesar. Deionized water (18 MΩ) was obtained from a Barnstead NANOpure Diamond water purification system. HPLC grade acetonitrile and deionized water, each containing 0.1% trifluoroacetic acid (TFA), were used for analytical and preparative reverse-phase HPLC. $2AM_{CC}$, and 2AT were synthesized as previously described.[2] $2AM_{CC}$, 2AT, KLM_{CC}, and KLT, were prepared and used as the trifluoroacetate salts and were assumed to have one trifluoroacetic acid molecule per amine group on the peptide.

Synthesis, purification, and characterization of KLM_{CC}.

Loading of the resin. 2-Chlorotrityl chloride resin (300 mg, 1.2 mmol/g) was added to a Bio-Rad Poly-Prep chromatography column (10 mL). The resin was suspended in dry CH_2Cl_2 (10 mL) and allowed to swell for 30 min. The solution was drained from the resin and a solution of Boc-Orn(Fmoc)-OH (0.50 equiv, 82 mg, 0.18 mmol) in 6% (v/v) 2,4,6-collidine in dry CH_2Cl_2 (8 mL) was added immediately and the suspension was gently agitated for 12 h. The solution was then drained and a mixture of CH_2Cl_2/MeOH/*N,N*-diisopropylethylamine (DIPEA) (17:2:1, 10 mL) was added immediately. The mixture was gently agitated for 1 h to cap the unreacted 2-chlorotrityl chloride resin sites. The resin was then washed with dry CH_2Cl_2 (2x) and dried by passing nitrogen through the vessel. This procedure typically yields 0.12–0.15 mmol of loaded resin (0.4–0.5 mmol/g loading).

Peptide coupling. The Boc-Orn(Fmoc)-2-chlorotrityl resin generated from the previous step was transferred to a peptide synthesis vessel and submitted to cycles of peptide coupling with Fmoc-protected amino acid building blocks. The linear peptide was synthesized from the *C*-terminus of the Orn connecting K_{16} and V_{36} to the *N*-terminus of K_{16}. Each coupling cycle consisted of i. Fmoc-deprotection with 20%

(v/v) piperidine in DMF for 5–10 min, ii. washing with DMF (3x), iii. coupling of the amino acid (0.75 mmol, 5 equiv) in the presence of HCTU (0.675 mmol, 4.5 equiv) and 20% (v/v) *N*-methylmorpholine (NMM) in DMF for 30 min, iv. washing with DMF (3x). The Fmoc-Ile-OH that follows the *N*-methyl glycine was double coupled (0.75 mmol, 5 equiv.) in the presence of HATU and HOAT (0.675 mmol, 4.5 equiv) and allowed to react for 1 h per coupling. After coupling of the last amino acid, the terminal Fmoc group was removed with 20% (v/v) piperidine in DMF. The resin was transferred from the peptide synthesis vessel to a Bio-Rad Poly-Prep chromatography column.

Cleavage of the peptide from the resin. The linear peptide was cleaved from the resin by agitating the resin for 1 h with a solution of 1,1,1,3,3,3-hexafluoroisopropanol (HFIP) in CH_2Cl_2 (1:4, 7 mL) in the Bio-Rad Poly-Prep column.[3] The suspension was filtered through the frit of the Poly-Prep column and the filtrate was collected in a 250-mL round-bottomed flask. The resin was washed with additional HFIP in CH_2Cl_2 (1:4, 7 mL) and then with CH_2Cl_2 (2×10 mL). The combined filtrates were concentrated by rotary evaporation to give a white solid. The white solid was further dried by vacuum pump to afford the crude protected linear peptide, which was cyclized without further purification.

Cyclization of the linear peptide. The crude protected linear peptide was dissolved in dry DMF (150 mL). HOBt (114 mg, 0.75 mmol, 5 equiv) and HBTU (317 mg, 0.75 mmol, 5 equiv) were added to the solution. DIPEA (0.33 mL, 1.8 mmol, 12 equiv) was added to the solution and the mixture was stirred under nitrogen for 24 h. The mixture was concentrated under reduced pressure to afford the crude protected cyclic peptide.

Global deprotection of the cyclic peptide. The protected cyclic peptide was dissolved in TFA/triisopropylsilane (TIPS)/H_2O (18:1:1, 20 mL) in a 250-mL round-bottomed flask equipped with a nitrogen-inlet adaptor. The solution was stirred for 1.5 h. The reaction mixture was then concentrated by rotary evaporation under reduced pressure to afford the crude cyclic peptide as a thin yellow film on the side of the round-bottomed flask. The crude cyclic peptide was immediately subjected to purification by reverse-phase HPLC (RP-HPLC), as described below.

Reverse-phase HPLC purification. The crude cyclic peptide was dissolved in H_2O and acetonitrile (7:3, 10 mL), and the solution was filtered through a 0.2 µm syringe filter. The filtrate was injected onto an Agilent Zorbax 300SB-C18 semi-preparative column (21.2 mm x 250 mm, 7 µm particle size) with an Agilent Prep 100Å C18 guard column (21.2 mm x 10 mm) on a Rainin Dynamax HPLC with a flow rate of 20.0 mL/min. The peptides were eluted with a gradient of acetonitrile (20–45% over 90 minutes). Elution was monitored at 214 nm with the accompanying DA Rainin HPLC software. Pure fractions were identified by analytical reverse-phase HPLC using an Agilent 1200 instrument equipped with a Phenomonex Aeris PEPTIDE 2.6u XB-C18 column and then combined and lyophilized to yield pure KLM_{CC} as a fluffy white solid. Synthesis of KLM_{CC} typically yielded 30–40 mg of the peptide as the TFA salt. The lyophilized KLM_{CC} was analyzed by LC/MS to confirm the purity of the peptide.

Synthesis, purification, and characterization of KLT.

Synthesis of KLT. KLT was synthesized by oxidizing KLM_{CC} in 20% aqueous DMSO.[4,5] A 6 mM solution of lyophilized KLM_{CC} was prepared gravimetrically by dissolving the peptide in an appropriate amount of 20% (v/v) aqueous DMSO prepared with deionized water. In a representative procedure, 30 mg of the KLM_{CC} TFA salt (0.013 mmol) was dissolved in 2.17 mL of 20% aqueous DMSO. The reaction was carried out in a capped 25 mL glass scintillation vial with gentle swirling (80–90 RPM) on a rotating platform at room temperature for 72 h.

LC/MS analysis of the KLM_{CC} oxidation reaction mixture. LC/MS analysis of the KLM_{CC} oxidation reaction mixture was performed after 72 h on an ACQUITY UPLC H-class system, Xevo G2-XS QTof (Waters Corp.) equipped with a Protein BEH C4 column (300 Å, 1.7 µm, 2.1 mm X 50 mm, Waters Corp). For LC/MS, the oxidation reaction mixture was diluted 100x by combining 2 µL of the oxidation reaction mixture with 198 µL of water. A 5 µL aliquot of the diluted oxidation reaction mixture was injected onto the column and eluted with gradient of Buffer A consisting of 0.1% formic acid in water

(Water LC-MS #9831-02, J.T. Baker; Formic Acid LC-MS #85178, Thermo Scientific) and Buffer B, acetonitrile (Acetonitrile UHPLC/MS #A956, Thermo Scientific). Gradient table listed below:

LC/MS elution gradient table (5 minute method)

time (min)	flow rate (mL/min)	%A	%B
initial	0.3	97	3
0.5	0.3	97	3
2	0.3	40	60
2.5	0.3	40	60
3	0.3	10	90
3.5	0.3	10	90
4	0.3	97	3
5	0.3	97	3

Purification of KLT. The KLM_{CC} oxidation reaction mixture was subjected to an initial reverse-phase LC purification by directly injecting the reaction mixture onto a Biotage® Isolera One flash chromatography instrument equipped with a Biotage® Isolera Sfär Bio C18 D - Duo 300 Å 20 µm 25 g column. The reaction mixture was injected at 20% aqueous CH_3CN and eluted with a gradient of 20–50% CH_3CN. Fractions containing KLT were identified by LC/MS as described above and then frozen and lyophilized to afford a white powder containing a mixture of products composed of predominantly KLT. The product mixture was then subjected to reverse-phase HPLC purification by dissolving the white powder in 1–3 mL of 20% aqueous CH_3CN and then injecting the solution onto an Agilent Zorbax 300SB-C18 semi-preparative column (21.2 mm x 250 mm, 7 µm particle size) with an Agilent Prep 100 Å C18 guard column (21.2 mm x 10 mm) on a Rainin Dynamax HPLC with a flow rate of 20.0 mL/min and eluted with a gradient of 20–45% CH_3CN over 90 min. In this HPLC purification, the column was submerged in a 60 ºC water bath heated by a thermal immersion circulator. Fractions containing pure KLT were identified by LC/MS using the 30-minute method described above and then combined, frozen, and

lyophilized to afford KLT as a white powder. The powder was analyzed by LC/MS in a similar fashion to that described above to confirm the purity of KLT.

In some instances, a significant number of fractions containing KLT from the HPLC purification were contaminated with intramolecular disulfide monomer. In these instances, the intramolecular disulfide monomer (~1.7 kDa) was removed from KLT (~5.4 kDa) by spin filtration as follows: a solution of the product mixture was prepared in 20 mL of 20% aqueous CH_3CN and then centrifuged through a 3 kDa molecular weight cutoff protein concentrator with a PES filter (Pierce™ catalog # 88526) at 3500 x g until the volume in the upper chamber of the spin filter reached 0.5–1 mL. Multiple rounds of centrifugation were performed (typically 3–6 rounds), replenishing the 20 mL of 20% aqueous CH_3CN at the beginning of each round. After the third round, the solution in the upper chamber was analyzed by LC/MS as described above to determine if all intramolecular disulfide monomer had been removed. If intramolecular disulfide monomer remained, additional rounds of centrifugation were performed. Once all the intramolecular disulfide monomer was removed, the solution in the upper chamber of the spin filter was transferred to a 15 mL conical tube. The reservoir of the upper chamber of the spin filter was rinsed with deionized water and the water rinse was transferred to the 15 mL conical tube. The solution was then frozen in dry ice and lyophilized to yield a fluffy white powder.

Typical syntheses yielded ~5 mg of >95% pure KLT as the TFA salt from a 0.1 mmol scale synthesis of KLM_{CC}. KLT_I was synthesized and purified in a similar fashion to KLT.

LC/MS characterization of KLT. The lyophilized KLT was analyzed by LC/MS to confirm the purity of the trimer. LC/MS analysis was performed on an ACQUITY UPLC H-class system, Xevo G2-XS QTof (Waters Corp.) equipped with a Protein BEH C4 column (300 Å, 1.7 µm, 2.1 mm X 50 mm, Waters Corp). For LC/MS, a 10 mg/mL solution of KLT was prepared gravimetrically by dissolving 1.0 mg of each peptide in 100 µL of deionized water. A 0.05 mg/mL solution of each peptide was then created by combining 1 µL of each 10 mg/mL solution with 199 µL of deionized water. The 0.05 mg/mL solution of KLT was then further diluted with deionized water to create a 0.005 mg/mL solution. A 5 µL portion

of each 0.005 mg/mL trimer solution was injected onto the column and eluted with gradient of Buffer A consisting of 0.1% formic acid in water (Water LC-MS #9831-02, J.T. Baker; Formic Acid LC-MS #85178, Thermo Scientific) and Buffer B, acetonitrile (Acetonitrile UHPLC/MS #A956, Thermo Scientific). Gradient table listed below:

LC/MS elution gradient table (30-minute method)

time (min)	flow rate (mL/min)	%A	%B
initial	0.3	97	3
1.0	0.3	97	3
25.0	0.3	50	50
27.0	0.3	10	90
27.5	0.3	10	90
29.0	0.3	97	3
30.0	0.05	97	3

X-ray crystallography of KLT and KLT_I.

Crystallization procedure for KLT and KLT_I. Initial crystallization conditions were determined using the hanging-drop vapor-diffusion method. Crystallization conditions were screened for KLT using three crystallization kits in a 96-well plate format (Hampton Index, PEG/Ion, and Crystal Screen). Three 150 nL hanging drops that differed in the ratio of peptide to well solution were made per condition in each 96-well plate for a total of 864 experiments. Hanging drops were made by combining an appropriate volume of KLT (10 mg/mL in 18 MΩ water) with an appropriate volume of well solution to create three 150 nL hanging drops with 1:1, 1:2, and 2:1 KLT:well solution. The hanging drops were made using a TTP LabTech Mosquito nanodisperse instrument. Crystals of KLT suitable for X-ray diffraction grew in a solution of 0.1 M Tris at pH 8.3, 0.2 M ammonium acetate, and 39% 2-methyl-2,4-pentanediol (MPD).

Crystallization conditions for KLT were optimized using a 4x6 matrix Hampton VDX 24-well plate. The pH of Tris was varied in each column in increments of 0.15 pH units (8.15, 8.30, 8.45, 8.60, 8.75, and 8.90) and the MPD concentration in each row in increments of 1% (39%, 40%, 41%, and 42%). For the first well in the 4x6 matrix we combined 100 µL of 1 M Tris buffer at pH 8.15, 390 µL of MPD, 100 µL of 2 M ammonium acetate, and 410 µL of 18 MΩ water. The other wells were prepared in

analogous fashion, by combining 100 μL of Tris buffer of varying pH, MPD in varying amounts, ammonium acetate, and 18 MΩ water for a total volume of 1 mL in each well.

Three hanging-drops were prepared per borosilicate glass slide by combining a 10 mg/mL solution of KLT (1 μL) and the well solution (1 μL) in a ratio of 1:1, 2:1, and 1:2. Slides were inverted and pressed firmly against the silicone grease surrounding each well. Crystals of KLT grew in ~96 h. Crystals were harvested with a nylon loop attached to a copper or steel pin and flash frozen in liquid nitrogen prior to data collection.

The X-ray crystallographic phases for KLT were determined by first synthesizing a variant of KLT with a *para*-iodo group on F_{19} (KLT_I) and using SAD phasing to determine the structure of KLT_I, and then using the structure of KLT_I as a search model in molecular replacement. This approach succeeded in determining the phases for KLT, because the crystal structure of KLT_I has the same space group and unit cell dimensions as the crystal structure of KLT. Figure S6 shows the asymmetric units of the crystal structures of KLT and KLT_I, which both contain two copies of their respective trimers.

Crystals of KLT_I suitable for X-ray diffraction grew in the same conditions as KLT, and crystallization conditions for KLT_I were optimized similarly.

X-ray crystallographic data collection, data processing, and structure determination for KLT and KLT_I. Diffraction data for KLT and KLT_I were collected on a Rigaku Micromax-007HF X-ray diffractometer with a rotating copper anode at 1.54 Å wavelength with 0.5° oscillation. Diffraction data were collected using CrystalClear. Diffraction data were scaled and merged using XDS and pointless and aimless.[6] Coordinates for the anomalous signals from the iodo groups on KLT_I were determined using HySS and then used in Autosol in the Phenix software suite to generate an electron density map.[7] The electron density map for KLT was generated using Phaser in the Phenix software suite using the asymmetric unit of the X-ray crystallographic structure of KLT_I as a search model in molecular replacement. Molecular manipulations of the models were performed with Coot.[8] Coordinates were

refined with phenix.refine. The optimal number and composition of the TLS groups were chosen automatically by phenix.refine.[9,10]

SDS-PAGE and silver staining.

SDS-PAGE. Running buffers for Tricine SDS-PAGE were prepared according to recipes detailed in Schägger, H. *Nat. Protoc.* 2006, *1*, 16–22.[11] 2AT and KLT were run on a 16.5% Mini-PROTEAN® Tris-Tricine Gel (Bio-Rad 4563063), and an 8–16% Mini-PROTEAN® TGX Stain-Free Protein Gel (Bio-Rad 4568105). Two molecular weight protein ladders were used: Spectra™ Multicolor Low Range Protein Ladder, ThermoFisher Scientific, catalog # 26628, and Precision Plus Protein™ Dual Xtra Prestained Protein Standards, Bio-Rad, catalog # 161-0377.

Sample preparation and gel running. Lyophilized 2AT and KLT were dissolved in deionized water to a concentration of 10 mg/mL. Aliquots of the 10 mg/mL solutions were then used to create 0.5 mg/mL solutions in 1X SDS-PAGE loading buffer (112.5 mM Tris buffer (pH 8.0) with 2% (w/v) SDS and 6% (v/v) glycerol). The 0.5 mg/mL solutions were then serially diluted in 1X SDS-PAGE loading buffer to create 0.25, 0.125, and 0.0625 mg/mL solutions. A 5.0-μL aliquot of each dilution was run on each gel. Once the gels were loaded, the gel-running apparatus was moved to a 4 °C cold room and allowed to equilibrate to 4 °C for 30 min. After equilibration, the gels were run at a constant 30 volts until adequate band separation was observed for the protein ladders.

Silver staining. Staining with silver nitrate was used to visualize 2AT and KLT in the gels. Reagents for silver staining were prepared according to procedures detailed in Simpson, R. J. *CSH Protoc.* 2007.[12] [The sodium thiosulfate solution, silver nitrate solution, and developing solution were prepared fresh each time silver staining was performed. Furthermore, the purity of the sodium carbonate in the developing solution was >99.5%].

For silver staining, the gel was removed from the cast and submerged and rocked in fixing solution (50% (v/v) methanol and 5% (v/v) acetic acid in deionized water) for 20 min. The fixing solution

was then discarded and the gel was rocked in 50% (v/v) aqueous methanol for 10 min. The 50% methanol was then discarded and the gel was rocked in deionized water for 10 min. The water was then discarded and the gel was soaked in 0.02% (w/v) sodium thiosulfate in deionized water for 1 min. The sodium thiosulfate was then discarded and the gel was rinsed with deionized water for 1 min (2X). After the last rinse, the gel was submerged in chilled 0.1% (w/v) silver nitrate in deionized water and rocked at 4 °C for 20 min. The silver nitrate solution was then discarded and the gel was rinsed with deionized water for 1 min (2X). To develop the gel, the gel was incubated in developing solution (2% (w/v) sodium carbonate, 0.04% (w/v) formaldehyde until the desired intensity of staining was reached (~1–3 min). When the desired intensity of staining was reached, the development was stopped by discarding the developing solution and submerging the gel in 5% aqueous acetic acid. The silver-stained gels were immediately visualized with a ChemiDoc™ MP Imaging System using the "Optimal Auto-exposure" exposure under the "Silver Stain Gel" application of the Image Lab Touch Software.

Circular dichroism spectroscopy.

A 50-μM solution of either 2AT or KLT was prepared by diluting an aliquot from a 10 mg/mL stock solution of each compound prepared in deionized water with 10 mM potassium phosphate buffer at pH 7.4. Each solution was transferred to a 1 mm quartz cuvette for data acquisition. CD spectra were acquired on a Jasco J-810 circular dichroism spectropolarimeter at room temperature. Data were collected using 0.2 nm intervals from 260 nm to 190 nm and averaged over five accumulations with smoothing.

Ultra-high mass range (UHMR) Orbitrap MS.

A 10 mg/mL stock solution of 2AT or KLT was prepared by dissolving an appropriate amount of lyophilized trimer in 18 MΩ deionized water. The 10 mg/mL solution of each trimer was diluted to 40 μM in 200 mM ammonium acetate immediately before each experiment. Experiments were conducted on a Thermo Q Exactive Ultra-High Mass Range (UHMR) Orbitrap MS (Bremen, Germany). All samples were

ionized via electronanospray ionization (nESI) using in-house pulled borosilicate capillaries with the capillary voltage applied to a platinum wire. nESI glass capillary tips were pulled in-house using a Sutter Instruments P-97 micropipette tip puller (Novato, CA). A capillary temperature of 250 °C was applied and a trap gas flow rate of 3–4 was used. The resolution was set to 6k for all complexes studied. In-source trapping was set to 60 V or less. The injection flatapole RF was set to 400 V. The HCD cell RF amplitude was set to 500 V. The C-trap RF amplitude was set to 2950 V. The bent flatapole RF was set to 500 V.

Mass spectra were analyzed and deconvolved using Unidec software[13] with settings as follows: m/z range 1000–8000 Th, charge range 1–30, mass range 1500–70000 Da, and smooth charge state distributions on. Peak detection range was set to 500 Da and peak detection threshold was set to 0.05. Oligomers were identified using the *Oligomer and Mass Tools*.

Ion mobility MS (IM-MS).

A 10 mg/mL stock solution of 2AT or KLT was prepared by dissolving an appropriate amount of lyophilized trimer in 18 MΩ deionized water. The 10 mg/mL solution of each trimer was diluted to 40 µM in 200 mM ammonium acetate immediately before each experiment. Experiments were conducted on a Waters SELECT SERIES Cyclic Ion Mobility Spectrometry Q-cIMS-TOF system. Samples were ionized using nESI. nESI glass capillary tips were pulled in-house using a Sutter Instruments P-97 micropipette tip puller (Novato, CA). When performing experiments on the cIMS, the cIMS was tuned as follows: capillary voltage, 0.4 to 0.6 kV; cone voltage, 20 V; source temperature, 25 °C; trap CE, 4 V; transfer CE, 4 V; trap and transfer gas, N_2 at 7.0 mL/min; IMS pressure, 1.8 mbar; TW static height, 22 V; TW velocity, 375 m/s; pushes per bin, 2. Spectra were analyzed in Waters Driftscope software.

Mass photometry.

A 10 mg/mL stock solution of 2AT or KLT was prepared by dissolving an appropriate amount of lyophilized trimer in 18 MΩ deionized water. The 10 mg/mL solution of each trimer was then diluted to

650 nM in 200 mM ammonium acetate. Microscope cover slips were cleaned with alternating washes of Milli-Q H_2O and isopropanol, then dried under nitrogen. Clean, sample well-containing silicone gaskets were added on top of the cover slips. Samples of 200 mM ammonium acetate containing no peptide were used as controls. NativeMark Unstained Protein Standard (Thermo Fisher) in 200 mM ammonium acetate was used as a protein ladder to create a calibration curve. NativeMark contains proteins of 1236, 1048, 720, 480, 242, 146, 66, and 20 kDa. For each acquisition, 20 μL of sample solution was introduced into the well and, following autofocus stabilization, movies of 60 s duration were recorded. Data was acquired using a TwoMP mass photometer (Refeyn Ltd, Oxford, UK) and analyzed using the associated software (DiscoverMP).

Cell-based toxicity assays of 2AT and KLT in SH-SY5Y cells.

The cellular toxicity of 2AT and KLT was assessed by measuring ATP levels, cell viability, and caspase-3/7 activation in SH-SY5Y cells exposed to a twofold dilution series (50–0.2 μM, 0 μM) of 2AT or KLT for 72 h. ATP levels, cell viability, and caspase-3/7 activation were measured using the following assays:

- ATP levels: CellTiter-Glo® 2.0 Cell Viability Assay (Promega Corporation; cat. # G9242).
- cell viability and caspase-3/7 activation: ApoLive-Glo™ Multiplex Assay (Promega Corporation; cat. # G6410).

Serial dilutions of 2AT and KLT were first prepared in a replica 96-well plate and then transferred to the plate containing cells. Three technical replicates were performed for each experiment. The plate layout for the experiments was as follows:

	1	2	3	4	5	6	7	8	9	10	11	12
A	media	media	media	media	media	media	media	media	media	media	media	media
B	media	50.0 µM 2AT	25.0 µM 2AT	12.5 µM 2AT	6.3 µM 2AT	3.1 µM 2AT	1.6 µM 2AT	0.8 µM 2AT	0.4 µM 2AT	0.2 µM 2AT	vehicle	media
C	media	50.0 µM 2AT	25.0 µM 2AT	12.5 µM 2AT	6.3 µM 2AT	3.1 µM 2AT	1.6 µM 2AT	0.8 µM 2AT	0.4 µM 2AT	0.2 µM 2AT	vehicle	media
D	media	50.0 µM 2AT	25.0 µM 2AT	12.5 µM 2AT	6.3 µM 2AT	3.1 µM 2AT	1.6 µM 2AT	0.8 µM 2AT	0.4 µM 2AT	0.2 µM 2AT	vehicle	media
E	media	50.0 µM KLT	25.0 µM KLT	12.5 µM KLT	6.3 µM KLT	3.1 µM KLT	1.6 µM KLT	0.8 µM KLT	0.4 µM KLT	0.2 µM KLT	vehicle	media
F	media	50.0 µM KLT	25.0 µM KLT	12.5 µM KLT	6.3 µM KLT	3.1 µM KLT	1.6 µM KLT	0.8 µM KLT	0.4 µM KLT	0.2 µM KLT	vehicle	media
G	media	50.0 µM KLT	25.0 µM KLT	12.5 µM KLT	6.3 µM KLT	3.1 µM KLT	1.6 µM KLT	0.8 µM KLT	0.4 µM KLT	0.2 µM KLT	vehicle	media
H	media	media	media	media	media	media	media	media	media	media	media	media

Preparation of SH-SY5Y cells for the toxicity assays. SH-SY5Y cells were plated in the inner 60 wells (rows B–G, columns 2–10) of cell culture-treated, black-walled, half-area, flat-bottom, clear-bottom 96-well plates (Corning™ cat. # 3882) at 30,000 cells/well. DMEM:F12 media (100 µL) was added to the outer wells (rows A and H and columns 1 and 12), to create an evaporative barrier and ensure the greatest reproducibility of data generated from the inner wells. The cells were plated in 50 µL of a 1:1 mixture of DMEM:F12 media supplemented with 10% fetal bovine serum, 100 U/mL penicillin, and 100 µg/mL streptomycin and incubated at 37 °C in a 5% CO_2 atmosphere for 24 hours to adhere the cells to the bottoms of the wells.

Preparation of 2AT and KLT for toxicity assays. 10 mg/mL stock solutions of the 2AT and KLT TFA salts were prepared gravimetrically by dissolving 1.0 mg of each compound in 100 µL of deionized water that had been passed through a 0.2 µm filter. The 10 mg/mL solution of the 2AT TFA salt is equivalent to 1.49 mM 2AT. The 10 mg/mL solution of the KLT TFA salt is equivalent to 1.47 mM KLT.

The 10 mg/mL stock solutions were prepared in a 1.7 mL microcentrifuge tube and stored at -20 ºC when not in use.

After the cells had adhered to the bottoms of the wells, the 10 mg/mL stock solutions of each trimer were used to create a replica 96-well plate (Corning™ cat. # 353075) containing a twofold dilution series (50–0.2 μM, 0 μM) of 2AT and KLT as follows: (1) Add 75 μL of serum-free, Phenol Red-free DMEM:F12 media to the wells in columns 3–11 of rows B–G of the 96-well plate. (2) In the wells of column 2 of rows B–D, prepare 150 μL of a 50 μM solution of 2AT by adding 5.0 μL of the 1.49 mM stock solution of 2AT to 145.0 μL of serum-free, Phenol Red-free DMEM:F12 media; in the wells of column 2 of rows E–G prepare 150 μL of a 50 μM solution of KLT by adding 5.1 μL of the 1.47 mM stock solution of KLT to 144.9 μL of serum-free, Phenol Red-free DMEM:F12 media. (3) using a multi-channel pipette, perform a twofold serial dilution of the trimers by transferring 75 μL of the 50-μM trimer solutions from column 2 to column 3 and mixing by pipetting up and down 8–10 times, and then transferring 75 μL from column 3 to column 4 and mixing, and so on, ending the serial dilution on column 9, with column 11 not receiving any trimer and thus constituting the 0-μM vehicle control.

Treatment of the SH-SY5Y cells with 2AT and KLT. A multi-channel pipette was used to remove the media from the wells of the 96-well plate containing cells. A multi-channel pipette was then used to immediately add 50 μL from each well of the 96-well replica plate containing the trimers to each respective well of the 96-well plate containing cells. The plate was incubated at 37 °C in a 5% CO_2 atmosphere for 72 hours and then the assays were performed according to manufacturer's instructions.

CellTiter-Glo® 2.0 Cell Viability Assay. After 72 h hours, ATP levels were measured using a CellTiter-Glo® 2.0 Cell Viability Assay according to the manufacturer's instructions, except the volumes of assay reagent added to the wells were halved, to accommodate the half-area wells. The 96-well plate was removed from the incubator and allowed to come to room temperature for 30 minutes. Once the plate reached room temperature, a 4 mL aliquot of CellTiter-Glo® 2.0 reagent was thawed and then added to a reagent reservoir (Thermo Scientific cat. # 8093-11). A multi-channel pipette was then used to transfer 50

μL of the CellTiter-Glo® 2.0 reagent to each well containing cells on the 96-well plate. The plate was then shaken on a rotating shaker for 2 minutes at 100 RPM. The luminescence from each well was then measured on a Promega GloMax® Discover Microplate Reader. The luminescence readings for the three replicates of each treatment group were averaged and the standard deviations were calculated using GraphPad Prism. The data were then plotted using GraphPad Prism.

ApoLive-Glo™ Multiplex Assay. After 72 h hours, cell viability and caspase-3/7 activation were measured using an ApoLive-Glo™ Multiplex Assay according to the manufacturer's instructions, except the volumes of assay reagent added to the wells were halved, to accommodate the half-area wells. The Viability Reagent and Caspase-Glo® 3/7 Reagent were prepared according to the instructions. The 96-well plate was removed from the incubator and 10 μL of the Viability Reagent was added to each well containing cells. The plate was then well-secured to a rotating shaker using laboratory tape and shaken vigorously (350 RPM) for 30 seconds, and then incubated at 37 °C for 30 minutes. The fluorescence from each well was then measured on Promega GloMax® Discover Microplate Reader ($450_{Ex}/505_{Em}$). The plate was then removed from the plate reader and 50 μL of the Caspase-Glo® 3/7 Reagent was added to each well containing cells. The plate was then well-secured to a rotating shaker using laboratory tape and shaken vigorously (350 RPM) for 30 seconds, and then incubated at room temperature for 30 minutes. The luminescence from each well was then measured on a Promega GloMax® Discover Microplate Reader. The fluorescence and luminescence readings for the three replicates of each treatment group were averaged and the standard deviations were calculated using GraphPad Prism. The data were then plotted using GraphPad Prism.

Preparation of 2AT-sCy3 and KLT-sCy3.

We developed a procedure to append one single fluorophore onto 2AT and KLT. In this procedure, excess molar equivalents of 2AT or KLT are treated with the sulfo-cyanine3 NHS ester (Lumiprobe, cat#

21320) in a molar ratio of 20:1 trimer to fluorophore. The singly labeled trimer is isolated by RP-HPLC, and the unlabeled trimer is collected and recycled.

A 5 mg portion of sulfo-cyanine3 NHS ester was dissolved in water and transferred into microcentrifuge tubes in 17 µg aliquots. Microcentrifuge tubes containing aliquoted sulfo-cyanine3 NHS ester were immediately moved to a -80 °C freezer and remained overnight. The microcentrifuge tubes containing frozen sulfo-cyanine3 NHS ester were then lyophilized.

A 75 mM sodium carbonate buffer solution was prepared gravimetrically and the pH was adjusted to 9.6. A 10 mg/mL solution of 2AT or KLT was prepared gravimetrically by dissolving the lyophilized trimer in the appropriate amount of 18 MΩ deionized water. A 300-µL aliquot of the 10 mg/mL trimer solution was added to 700 µL of 75 mM sodium carbonate buffer in a clean 1.7 mL microcentrifuge tube. A 40-µL aliquot of the reaction mixture was transferred into the tube containing the frozen 17 µg aliquot of sulfo-cyanine3 NHS ester to thaw the fluorophore, and then immediately transferred back to the larger trimer solution. The reaction was protected from light with black felt and rocked gently for 1 h. After 1 h, the reaction was directly injected onto an HPLC.

The fluorescently labeled trimers were purified by semi-preparative reverse-phase HPLC using an Agilent Zorbax 300SB-C18 semi-preparative column (9.4 mm x 250 mm, 5 µm particle size) with a ZORBAX 300SB-C3 preparative guard column (9.4 x 15 mm) on a Rainin Dynamax HPLC with a flow rate of 5.0 mL/min. The C18 column and the guard column were heated to 60 °C in a water bath. The trimers were eluted with a gradient of acetonitrile (20–45% over 90 minutes). Elution was monitored at 214 nm with the accompanying DA Rainin HPLC software. Pure fractions were combined and lyophilized. Purified 2AT-sCy3 and KLT-sCy3 were characterized by LC-MS.

Stock solutions of 2AT-sCy3 and KLT-sCy3 were prepared spectrophotometrically. Lyophilized peptide was dissolved in 18 MΩ deionized water and the absorbance was measured using the λ_{max} of sCy3 (λ_{max}= 548 nm). The published molar extinction coefficient for sCy3 (162,000 L·mol^{-1}·cm^{-1}) was used to calculate sample concentration.

Live-cell fluorescence microscopy of SH-SY5Y cells treated with 2AT-sCy3 and KLT-sCy3.

SH-SY5Y cells were plated in an Ibidi μ-Slide 8 Well Chamber Slide (Ibidi cat. # 80826) at 80,000 cells per well. Cells were incubated in 500 μL of a 1:1 mixture of DMEM:F12 media supplemented with 10% fetal bovine serum, 100 U/mL penicillin, and 100 μg/mL streptomycin at 37 °C in a 5% CO_2 atmosphere and allowed to adhere to the bottom of the slide for 48 hours. Solutions of 2AT-sCy3 or KLT-sCy3 were prepared to a final volume of 200 μL and a final concentration of 1 μM in either serum-free, Phenol Red-free 1:1 DMEM/F12 media or serum-free, Phenol Red-free 1:1 DMEM/F12 media containing 100 nM LysoTracker Green DND-26 (ThermoFisher).

After the cells had adhered, the media was removed and replaced with the 1 μM solutions of 2AT-sCy3 or KLT-sCy3 with and without Lysotracker Green DND-26. Control wells were treated with media containing no trimer. The slide was incubated at 37 °C in a 5% CO_2 atmosphere chamber for 8 hours. The media was removed from the wells and replaced with 200 μL of 1 μg/mL Hoechst 33342 in serum-free, Phenol Red-free 1:1 DMEM:F12 media. After 30 minutes, the Hoechst-containing media was removed and replaced with 200 μL of serum-free, Phenol Red-free 1:1 DMEM:F12 media.

The cells were imaged using a Keyence BZ-X810 fluorescence microscope. Images were collected with a 60x oil immersion objective lens. Micrographs of treated cells were recorded using the DAPI filter cube [excitation wavelength = 350/50 nm (325–375 nm) and emission wavelength = 460/50 nm (435–485nm)] for Hoechst 33342 nuclear marker, the Cy3 filter cube [excitation wavelength = 545/25 nm (532.5–557.5 nm) and emission wavelength = 605/70 nm (570–640 nm)] for 2AT-sCy3 and KLT-sCy3, and the GFP filter cube [excitation wavelength = 470/40 nm (450–490 nm) and emission wavelength = 525/50 nm (500–550 nm)] for Lysotracker Green DND-26 (ThermoFisher). The image brightness of the channels was adjusted using BZ-X810 Analyzer software.

Preparation of Aβ_{42} for the ThT aggregation assays and cell-based toxicity assays.

A 1 mg portion of recombinantly expressed Aβ_{42} as the ammonium salt was purchased from rPeptide (catalog# A-1167-2) and received as a fluffy lyophilized solid in a glass amber vial. The 1 mg Aβ_{42} portion was dissolved with 1 mL of 2 mM NaOH to create a 1 mg/mL Aβ_{42} solution. The 1 mg/mL Aβ_{42} solution was then sonicated in a water bath sonicator for 5 minutes. After sonication, 0.02 μmol aliquots of Aβ_{42} were prepared by transferring 92.6 μL portions of the 1 mg/mL Aβ_{42} solution to low-binding microcentrifuge tubes (Axygen, catalog# MCT-175-L-C) containing a hole in the lid of the tube created by puncturing the lid with a 22 gauge needle. The aliquots were then frozen on dry ice for 1 hour, transferred to a lyophilization vessel, and lyophilized overnight. The next day, the aliquots were removed from the lyophilizer and each microcentrifuge tube was immediately transferred to a 50 mL conical tube. The 50 mL conical tubes were sealed by tightening the lid and stored at -80 °C until use.

ThT aggregation assay of Aβ_{42} in the presence of 2AT or KLT.

Assay notes: ThT assays were performed on 5 μM Aβ_{42} in PBS at pH 7.4 (10 mM Na_2HPO_4, 1.8 mM KH_2PO_4, 137 mM NaCl, 2.7 mM KCl) containing 10 μM ThT in the presence of a dilution series of 2AT or KLT (10–0.01 μM, 0 μM). ThT was prepared fresh each time the assay was performed. The assays were performed in triplicate in Corning® 96-well Half Area Black/Clear Flat Bottom Polystyrene NBS Microplates (product# 3881) at 25 °C under quiescent conditions.

A 0.02 μmol aliquot of Aβ_{42} was removed from the -80 °C freezer and allowed to equilibrate to room temperature. During this equilibration time, an 11 μM solution of thioflavin T (ThT) was prepared in PBS at pH 7.4 (10 mM Na_2HPO_4, 1.8 mM KH_2PO_4, 137 mM NaCl, 2.7 mM KCl) and a serial dilution series of 10x concentrations of 2AT and KLT were prepared in deionized water.

Preparation of 11 μM ThT in PBS. To prepare the 11 μM ThT solution in PBS, a concentrated solution of ThT (1–3 mM) was prepared in a 15 mL polypropylene conical tube by adding ~5–7 mg of

ThT (TCI Chemicals, catalog# T0558) to 7–10 mL PBS. The concentrated ThT solution was sonicated in a water bath sonicator for ~5 minutes and then passed through a 0.2 μm nylon syringe filter into a new 15 mL conical tube. The concentration of the concentrated ThT solution was determined photospectrometrically by first preparing 2 mL of a 1:200 diluted ThT solution (0.010 mL of the concentrated ThT solution into 1.990 mL PBS). The absorbance of the diluted ThT solution was then measured at 412 nm in a 1 cm quartz cuvette and the concentration was calculated using an estimated extinction coefficient (ε) of 36,000 M^{-1} cm^{-1}. The concentration of the diluted ThT solution was multiplied by 200 to calculate the concentration of the concentrated ThT solution. A 10 mL portion of 11 μM ThT in PBS was prepared by diluting an appropriate volume of the concentrated ThT solution with PBS. The 11 μM ThT solution was then kept on ice until used.

Preparation of 2AT and KLT for the ThT assay. 10 mg/mL stock solutions of the 2AT and KLT TFA salts were prepared gravimetrically by dissolving 1.0 mg of each compound in 100 μL of deionized water that had been passed through a 0.2 μm filter. The 10 mg/mL solution of the 2AT TFA salt is equivalent to 1.49 mM 2AT. The 10 mg/mL solution of the KLT TFA salt is equivalent to 1.47 mM KLT. The 10 mg/mL stock solutions were prepared in a 1.7 mL microcentrifuge tube and stored at -20 ºC when not in use.

A twofold dilution series containing 10x concentrations of 2AT or KLT (100–0.1 μM, 0 μM) was prepared in rows A and B of the 96-well assay plate (2AT in row A; KLT in row B). To prepare these dilution series, 30 μL of deionized water was added to wells A1 and B1, and 15 μL of deionized water was added to wells A2–A12 and wells B2–B12. For 2AT, 2.013 μL of deionized water was removed from well A1 and replaced with 2.013 μL of 1.49 mM 2AT to create a 100 μM solution of 2AT; for KLT, 2.041 μL of deionized water was removed from well B1 and replaced with 2.041 μL of 1.47 mM KLT to create a 100 μM solution of KLT. The serial dilution was then performed by transferring 15 μL from wells A1 or B1 to wells A2 or B2 and then mixing by pipetting up and down 8–12 times, and so on, stopping on wells A11 or B11. Wells A12 and B12 did not receive 2AT or KLT and only contained 15 μL of deionized

water. The red wells in the plate map below show the plate layout of the 2AT and KLT dilution series. A 12-channel pipette was then used to transfer 4 µL portions of the 2AT solutions from all 12 wells of row A to all 12 wells in rows C, D, and E, and then transfer 4 µL portions of the KLT solutions from all 12 wells of row B to all 12 wells in rows F, G, and H. After the dilution series was prepared and aliquoted to the respective rows, the 96-well assay plate was kept on ice for the remainder of the experimental setup.

Adding $A\beta_{42}$ to the ThT assay plate. After the 11 µM ThT solution was prepared and the 2AT and KLT dilution series was completed, a 5.56 µM solution of $A\beta_{42}$ was prepared in 11 µM ThT. To prepare the 5.56 µM solution of $A\beta_{42}$, the equilibrated 0.02 µmol $A\beta_{42}$ aliquot was first dissolved in 92.6 µL deionized water and sonicated in a water bath sonicator for 5 minutes. During the sonication time, 2.507 mL of ice-cold 11 µM ThT was transferred to a 15 mL conical tube and kept on ice. After sonication, 1 mL of ice-cold 11 µM ThT was added to the $A\beta_{42}$ solution and mixed by pipetting up and down 4 times, and then quickly transferred to the 15 mL conical tube containing the 2.507 mL portion of ice-cold 11 µM ThT to create the 5.56 µM $A\beta_{42}$ solution. The $A\beta_{42}$ solution was then dumped into a sterile 25 mL reagent reservoir (ThermoFisher, catalog# 8093-11) and a 12-channel pipette was used to transfer 36 µL of the $A\beta_{42}$ solution to rows C–H, creating the green plate map shown below.

	1	2	3	4	5	6	7	8	9	10	11	12
A	100 µM<br>2AT	50 µM<br>2AT	25 µM<br>2AT	12.5 µM<br>2AT	6.25 µM<br>2AT	3.13 µM<br>2AT	1.56 µM<br>2AT	0.78 µM<br>2AT	0.39 µM<br>2AT	0.20 µM<br>2AT	0.10 µM<br>2AT	0.0 µM<br>2AT
B	100 µM<br>KLT	50 µM<br>KLT	25 µM<br>KLT	12.5 µM<br>KLT	6.25 µM<br>KLT	3.13 µM<br>KLT	1.56 µM<br>KLT	0.78 µM<br>KLT	0.39 µM<br>KLT	0.20 µM<br>KLT	0.10 µM<br>KLT	0.0 µM<br>KLT
C	10 µM<br>2AT<br>5 µM $A\beta_{42}$	5 µM<br>2AT<br>5 µM $A\beta_{42}$	2.5 µM<br>2AT<br>5 µM $A\beta_{42}$	1.25 µM<br>2AT<br>5 µM $A\beta_{42}$	0.625 µM<br>2AT<br>5 µM $A\beta_{42}$	0.313 µM<br>2AT<br>5 µM $A\beta_{42}$	0.156 µM<br>2AT<br>5 µM $A\beta_{42}$	0.078 µM<br>2AT<br>5 µM $A\beta_{42}$	0.039 µM<br>2AT<br>5 µM $A\beta_{42}$	0.020 µM<br>2AT<br>5 µM $A\beta_{42}$	0.010 µM<br>2AT<br>5 µM $A\beta_{42}$	0.0 µM<br>2AT<br>5 µM $A\beta_{42}$
D	10 µM<br>2AT<br>5 µM $A\beta_{42}$	5 µM<br>2AT<br>5 µM $A\beta_{42}$	2.5 µM<br>2AT<br>5 µM $A\beta_{42}$	1.25 µM<br>2AT<br>5 µM $A\beta_{42}$	0.625 µM<br>2AT<br>5 µM $A\beta_{42}$	0.313 µM<br>2AT<br>5 µM $A\beta_{42}$	0.156 µM<br>2AT<br>5 µM $A\beta_{42}$	0.078 µM<br>2AT<br>5 µM $A\beta_{42}$	0.039 µM<br>2AT<br>5 µM $A\beta_{42}$	0.020 µM<br>2AT<br>5 µM $A\beta_{42}$	0.010 µM<br>2AT<br>5 µM $A\beta_{42}$	0.0 µM<br>2AT<br>5 µM $A\beta_{42}$
E	10 µM<br>2AT<br>5 µM $A\beta_{42}$	5 µM<br>2AT<br>5 µM $A\beta_{42}$	2.5 µM<br>2AT<br>5 µM $A\beta_{42}$	1.25 µM<br>2AT<br>5 µM $A\beta_{42}$	0.625 µM<br>2AT<br>5 µM $A\beta_{42}$	0.313 µM<br>2AT<br>5 µM $A\beta_{42}$	0.156 µM<br>2AT<br>5 µM $A\beta_{42}$	0.078 µM<br>2AT<br>5 µM $A\beta_{42}$	0.039 µM<br>2AT<br>5 µM $A\beta_{42}$	0.020 µM<br>2AT<br>5 µM $A\beta_{42}$	0.010 µM<br>2AT<br>5 µM $A\beta_{42}$	0.0 µM<br>2AT<br>5 µM $A\beta_{42}$
F	10 µM<br>KLT<br>5 µM $A\beta_{42}$	5 µM<br>KLT<br>5 µM $A\beta_{42}$	2.5 µM<br>KLT<br>5 µM $A\beta_{42}$	1.25 µM<br>KLT<br>5 µM $A\beta_{42}$	0.625 µM<br>KLT<br>5 µM $A\beta_{42}$	0.313 µM<br>KLT<br>5 µM $A\beta_{42}$	0.156 µM<br>KLT<br>5 µM $A\beta_{42}$	0.078 µM<br>KLT<br>5 µM $A\beta_{42}$	0.039 µM<br>KLT<br>5 µM $A\beta_{42}$	0.020 µM<br>KLT<br>5 µM $A\beta_{42}$	0.010 µM<br>KLT<br>5 µM $A\beta_{42}$	0.0 µM<br>KLT<br>5 µM $A\beta_{42}$
G	10 µM<br>KLT<br>5 µM $A\beta_{42}$	5 µM<br>KLT<br>5 µM $A\beta_{42}$	2.5 µM<br>KLT<br>5 µM $A\beta_{42}$	1.25 µM<br>KLT<br>5 µM $A\beta_{42}$	0.625 µM<br>KLT<br>5 µM $A\beta_{42}$	0.313 µM<br>KLT<br>5 µM $A\beta_{42}$	0.156 µM<br>KLT<br>5 µM $A\beta_{42}$	0.078 µM<br>KLT<br>5 µM $A\beta_{42}$	0.039 µM<br>KLT<br>5 µM $A\beta_{42}$	0.020 µM<br>KLT<br>5 µM $A\beta_{42}$	0.010 µM<br>KLT<br>5 µM $A\beta_{42}$	0.0 µM<br>KLT<br>5 µM $A\beta_{42}$
H	10 µM<br>KLT<br>5 µM $A\beta_{42}$	5 µM<br>KLT<br>5 µM $A\beta_{42}$	2.5 µM<br>KLT<br>5 µM $A\beta_{42}$	1.25 µM<br>KLT<br>5 µM $A\beta_{42}$	0.625 µM<br>KLT<br>5 µM $A\beta_{42}$	0.313 µM<br>KLT<br>5 µM $A\beta_{42}$	0.156 µM<br>KLT<br>5 µM $A\beta_{42}$	0.078 µM<br>KLT<br>5 µM $A\beta_{42}$	0.039 µM<br>KLT<br>5 µM $A\beta_{42}$	0.020 µM<br>KLT<br>5 µM $A\beta_{42}$	0.010 µM<br>KLT<br>5 µM $A\beta_{42}$	0.0 µM<br>KLT<br>5 µM $A\beta_{42}$

(red) wells used in serial dilution of 2AT and KLT (green) wells used in assay

Reading the ThT assay plate. The ThT assay plate was sealed with a clear adhesive plate seal (Axygen, catalog# PCR-SP) and was immediately inserted into a ThermoFisher Scientific Varioskan Lux plate reader. Fluorescence measurements of each well in rows C–H were acquired every 2 minutes over a 5 hour period with the following parameters:

excitation: 440 nm emission: 485 nm

measurement time: 1000 ms optics: bottom read

excitation bandwidth: 12 nm

The data were plotted in GraphPad Prism.

Cell-based toxicity assays of Aβ$_{42}$ in the absence or presence of 2AT and KLT in SH-SY5Y cells.

The cellular toxicity of Aβ$_{42}$ in the absence or presence of 2AT and KLT was assessed by measuring LDH release, ATP levels, caspase-3/7 activation, and cell viability in SH-SY5Y cells exposed to 6 μM Aβ$_{42}$ in the presence of a twofold dilution series of 2AT or KLT (12–0.09 μM, 0 μM) for 72 h. LDH release, ATP levels, caspase-3/7 activation, and cell viability were measured using the following assays:

- LDH release: CyQUANT™ LDH Cytotoxicity Assay (ThermoFisher Scientific; cat# C20301)
- ATP levels: CellTiter-Glo® 2.0 Cell Viability Assay (Promega Corporation; cat. # G9242).
- caspase-3/7 activation and cell viability: ApoLive-Glo™ Multiplex Assay (Promega Corporation; cat. # G6410).

Two 96-well plates of cells were prepared as described below. The LDH release assay and CellTiter-Glo assay were performed on one plate of cells; the ApoLive-Glo assay was performed on the other plate of cells. Three technical replicates were performed for each experiment. The plate layout for the two plates in the experiment was as follows:

	1	2	3	4	5	6	7	8	9	10	11	12
A	media	media	media	media	media	media	media	media	media	media	media	media
B	media	12 μM<br>2AT<br>6 μM $A\beta_{42}$	6 μM<br>2AT<br>6 μM $A\beta_{42}$	3 μM<br>2AT<br>6 μM $A\beta_{42}$	1.5 μM<br>2AT<br>6 μM $A\beta_{42}$	0.725 μM<br>2AT<br>6 μM $A\beta_{42}$	0.37 μM<br>2AT<br>6 μM $A\beta_{42}$	0.19 μM<br>2AT<br>6 μM $A\beta_{42}$	0.09 μM<br>2AT<br>6 μM $A\beta_{42}$	0.0 μM<br>2AT<br>6 μM $A\beta_{42}$	vehicle	media
C	media	12 μM<br>2AT<br>6 μM $A\beta_{42}$	6 μM<br>2AT<br>6 μM $A\beta_{42}$	3 μM<br>2AT<br>6 μM $A\beta_{42}$	1.5 μM<br>2AT<br>6 μM $A\beta_{42}$	0.725 μM<br>2AT<br>6 μM $A\beta_{42}$	0.37 μM<br>2AT<br>6 μM $A\beta_{42}$	0.19 μM<br>2AT<br>6 μM $A\beta_{42}$	0.09 μM<br>2AT<br>6 μM $A\beta_{42}$	0.0 μM<br>2AT<br>6 μM $A\beta_{42}$	vehicle	media
D	media	12 μM<br>2AT<br>6 μM $A\beta_{42}$	6 μM<br>2AT<br>6 μM $A\beta_{42}$	3 μM<br>2AT<br>6 μM $A\beta_{42}$	1.5 μM<br>2AT<br>6 μM $A\beta_{42}$	0.725 μM<br>2AT<br>6 μM $A\beta_{42}$	0.37 μM<br>2AT<br>6 μM $A\beta_{42}$	0.19 μM<br>2AT<br>6 μM $A\beta_{42}$	0.09 μM<br>2AT<br>6 μM $A\beta_{42}$	0.0 μM<br>2AT<br>6 μM $A\beta_{42}$	vehicle	media
E	media	12 μM<br>KLT<br>6 μM $A\beta_{42}$	6 μM<br>KLT<br>6 μM $A\beta_{42}$	3 μM<br>KLT<br>6 μM $A\beta_{42}$	1.5 μM<br>KLT<br>6 μM $A\beta_{42}$	0.725 μM<br>KLT<br>6 μM $A\beta_{42}$	0.37 μM<br>KLT<br>6 μM $A\beta_{42}$	0.19 μM<br>KLT<br>6 μM $A\beta_{42}$	0.09 μM<br>KLT<br>6 μM $A\beta_{42}$	0.0 μM<br>KLT<br>6 μM $A\beta_{42}$	vehicle	media
F	media	12 μM<br>KLT<br>6 μM $A\beta_{42}$	6 μM<br>KLT<br>6 μM $A\beta_{42}$	3 μM<br>KLT<br>6 μM $A\beta_{42}$	1.5 μM<br>KLT<br>6 μM $A\beta_{42}$	0.725 μM<br>KLT<br>6 μM $A\beta_{42}$	0.37 μM<br>KLT<br>6 μM $A\beta_{42}$	0.19 μM<br>KLT<br>6 μM $A\beta_{42}$	0.09 μM<br>KLT<br>6 μM $A\beta_{42}$	0.0 μM<br>KLT<br>6 μM $A\beta_{42}$	vehicle	media
G	media	12 μM<br>KLT<br>6 μM $A\beta_{42}$	6 μM<br>KLT<br>6 μM $A\beta_{42}$	3 μM<br>KLT<br>6 μM $A\beta_{42}$	1.5 μM<br>KLT<br>6 μM $A\beta_{42}$	0.725 μM<br>KLT<br>6 μM $A\beta_{42}$	0.37 μM<br>KLT<br>6 μM $A\beta_{42}$	0.19 μM<br>KLT<br>6 μM $A\beta_{42}$	0.09 μM<br>KLT<br>6 μM $A\beta_{42}$	0.0 μM<br>KLT<br>6 μM $A\beta_{42}$	vehicle	media
H	media	media	media	media	media	media	media	media	media	media	media	media

Preparation of SH-SY5Y cells for the toxicity assays. Two 96-well plates of SH-SY5Y cells were created by plating the cells in the inner 60 wells (rows B–G, columns 2–10) of cell culture-treated, black-walled, half-area, flat-bottom, clear-bottom 96-well plates (Corning™ cat. # 3882) at 30,000 cells/well. DMEM:F12 media (100 μL) was added to the outer wells (rows A and H and columns 1 and 12), to create an evaporative barrier and ensure the greatest reproducibility of data generated from the inner wells. The cells were plated in 50 μL of a 1:1 mixture of DMEM:F12 media supplemented with 10% fetal bovine serum, 100 U/mL penicillin, and 100 μg/mL streptomycin and incubated at 37 °C in a 5% CO_2 atmosphere for 24 hours to adhere the cells to the bottoms of the wells.

Preparation of $A\beta_{42}$ in the absence or presence of 2AT and KLT for toxicity assays. Two 0.02 μmol portions of $A\beta_{42}$ were removed from the -80 °C freezer and allowed to equilibrate to room temperature. 10 mg/mL stock solutions of the 2AT and KLT TFA salts were prepared gravimetrically by dissolving 1.0 mg of each compound in 100 μL of deionized water that had been passed through a 0.2 μm filter. The 10 mg/mL solution of the 2AT TFA salt is equivalent to 1.49 mM 2AT. The 10 mg/mL solution of the KLT

TFA salt is equivalent to 1.47 mM KLT. The 10 mg/mL stock solutions were prepared in a 1.7 mL microcentrifuge tube and stored at -20 ºC when not in use.

After the cells had adhered to the bottoms of the wells, twofold dilution series containing 10x concentrations of 2AT or KLT (120–0.9 μM, 0 μM) were prepared in two sets of 10 (10 tubes for 2AT; 10 tubes for KLT) low-binding microcentrifuge tubes (Axygen, catalog# MCT-175-L-C). To create this dilution series, 64 μL of filter-sterilized deionized water was added to the first tube in each set, and 32 μL of filter-sterilized deionized water was added to the remaining 9 tubes in each set. To create the 120 μM solution of 2AT, 5.15 μL of water was removed from the first tube and replaced with 5.15 μL of the 1.49 mM 2AT solution prepared previously. To create the 120 μM solution of KLT, 5.22 μL of water was removed from the first tube and replaced with 5.22 μL of the 1.47 mM KLT solution prepared previously. The serial dilution was then performed on each set of tubes, transferring 32 μL portions each time, mixing by pipetting up and down 8–12 times, and ending the serial dilution on tube 8. Tube 9 in each set did not receive any trimer and would eventually only contain 6 μM $A\beta_{42}$. Tube 10 in each set did not receive any trimer nor would receive any $A\beta_{42}$ and would serve as the vehicle control. The entire 32 μL portion from the 10 tubes in each set was then transferred to 10 wells in row A for 2AT and 10 wells in row B for KLT in a standard sterile 96-well plate.

After the serial dilution and transfer of 2AT and KLT to a 96-well plate was completed, a 6.67 μM solution of $A\beta_{42}$ was prepared in phenol red-free, serum-free DMEM:F12 media. To prepare the 6.67 μM solution of $A\beta_{42}$, the two equilibrated 0.02 μmol $A\beta_{42}$ aliquots were each dissolved in 92.6 μL deionized water and sonicated in a water bath sonicator for 5 minutes. The sonicated $A\beta_{42}$ solutions were then combined and transferred to a 15 mL conical tube and diluted with 5.814 mL of phenol red-free, serum-free DMEM:F12 media to create the 6.67 μM solution of $A\beta_{42}$. The 6.67 μM solution of $A\beta_{42}$ was then dumped into a sterile 25 mL reagent reservoir (ThermoFisher, catalog# 8093-11) and a 12-channel pipette was used to transfer 288 μL of the $A\beta_{42}$ solution to each the 10 wells of rows A and B of the 96-well plate containing the 2AT and KLT dilution series and deionized water.

Treatment of the SH-SY5Y cells with Aβ_{42} in the absence or presence of 2AT and KLT. Using a multi-channel pipette, the media was removed from the wells containing cells in one of the 96-well plates. A multi-channel pipette was then used to immediately add 50 µL from the 10 wells in row A of the 96-well plate containing the 2AT/Aβ_{42} solutions to the wells containing cells in rows B, C, and D of the plate, and 50 µL from the 10 wells in row B of the 96-well plate containing the KLT/Aβ_{42} solutions to the wells containing cells in rows E, F, and G. This procedure was repeated for the second plate of cells. The plates were then incubated at 37 °C in a 5% CO_2 atmosphere for 72 hours and then the assays were performed according to manufacturer's instructions.

CyQUANT™ LDH Cytotoxicity Assay. After 72 h hours, LDH release was measured using a CyQUANT™ LDH Cytotoxicity Assay according to the manufacturer's instructions, except the volumes of assay reagent added to the wells were halved, to accommodate the half-area wells. A 50 µL aliquot of the supernatant media from each well was transferred to a new 96-well plate and 50 µL of LDH substrate solution, prepared according to manufacturer's protocol, was added to each well. The treated plates were stored in the dark for 30 min. The absorbance of each well was measured at 490 nm on a ThermoFisher Scientific Varioskan Lux plate reader. The absorbance measurements for the three replicates of each treatment group were averaged and the standard deviations were calculated using GraphPad Prism. The data were then plotted using GraphPad Prism.

CellTiter-Glo® 2.0 Cell Viability Assay. After 72 h hours, ATP levels were measured using a CellTiter-Glo® 2.0 Cell Viability Assay according to the manufacturer's instructions, except the volumes of assay reagent added to the wells were halved, to accommodate the half-area wells. The 96-well plate was removed from the incubator and allowed to come to room temperature for 30 minutes. Once the plate reached room temperature, a 4 mL aliquot of CellTiter-Glo® 2.0 reagent was thawed and then added to a reagent reservoir (Thermo Scientific cat. # 8093-11). A multi-channel pipette was then used to transfer 50 µL of the CellTiter-Glo® 2.0 reagent to each well containing cells on the 96-well plate. The plate was then shaken on a rotating shaker for 2 minutes at 100 RPM. The luminescence from each well was then

measured on a Promega GloMax® Discover Microplate Reader. The luminescence readings for the three replicates of each treatment group were averaged and the standard deviations were calculated using GraphPad Prism. The data were then plotted using GraphPad Prism.

ApoLive-Glo™ Multiplex Assay. After 72 h hours, cell viability and caspase-3/7 activation were measured using an ApoLive-Glo™ Multiplex Assay according to the manufacturer's instructions, except the volumes of assay reagent added to the wells were halved, to accommodate the half-area wells. The Viability Reagent and Caspase-Glo® 3/7 Reagent were prepared according to the instructions. The 96-well plate was removed from the incubator and 10 μL of the Viability Reagent was added to each well containing cells. The plate was then well-secured to a rotating shaker using laboratory tape and shaken vigorously (350 RPM) for 30 seconds, and then incubated at 37 °C for 30 minutes. The fluorescence from each well was then measured on Promega GloMax® Discover Microplate Reader ($450_{Ex}/505_{Em}$). The plate was then removed from the plate reader and 50 μL of the Caspase-Glo® 3/7 Reagent was added to each well containing cells. The plate was then well-secured to a rotating shaker using laboratory tape and shaken vigorously (350 RPM) for 30 seconds, and then incubated at room temperature for 30 minutes. The luminescence from each well was then measured on a Promega GloMax® Discover Microplate Reader. The fluorescence and luminescence readings for the three replicates of each treatment group were averaged and the standard deviations were calculated using GraphPad Prism. The data were then plotted using GraphPad Prism.

Establishment of a concentration of $A\beta_{42}$ that elicits significant toxicity. To establish an $A\beta_{42}$ concentration that elicits significant toxicity, we performed the cellular toxicity assays as described above on SH-SY5Y cells exposed to a dilution series of $A\beta_{42}$ (12.0 μM–0.05 μM) for 72 h. Figure S6 shows the data from this experiment.

Live-cell fluorescence microscopy of SH-SY5Y cells treated with HiLyte™ Fluor 647-Aβ$_{42}$ in the absence or presence of 2AT or KLT.

Preparation of SH-SY5Y cells for live-cell fluorescence microscopy. SH-SY5Y cells were plated in an Ibidi µ-Slide 8 Well Chamber Slide (Ibidi cat. # 80826) at 80,000 cells per well. Cells were incubated in 500 µL of a 1:1 mixture of DMEM:F12 media supplemented with 10% fetal bovine serum, 100 U/mL penicillin, and 100 µg/mL streptomycin at 37 °C in a 5% CO_2 atmosphere and allowed to adhere to the bottom of the slide for 48 hours.

Preparation of HiLyte™ Fluor 647-Aβ$_{42}$ in the absence or presence of 2AT and KLT for live-cell fluorescence microscopy. A 0.1 mg portion of *N*-terminally labeled HiLyte™ Fluor 647-Aβ$_{42}$ (HL647-Aβ$_{42}$) was purchased from AnaSpec (catalog# AS-64161) and received as a lyophilized powder in a glass amber vial. The 0.1 mg HL647-Aβ$_{42}$ portion was dissolved with 1 mL of 2 mM NaOH to create a 1 mg/mL HL647-Aβ$_{42}$ solution. The 1 mg/mL HL647-Aβ$_{42}$ solution was then sonicated in a water bath sonicator for 5 minutes. After sonication, 0.0025 µmol aliquots of HL647-Aβ$_{42}$ were prepared by transferring 14 µL portions of the 1 mg/mL HL647-Aβ$_{42}$ solution to low-binding microcentrifuge tubes (Axygen, catalog# MCT-175-L-C) containing a hole in the lid of the tube created by puncturing the lid with a 22 gauge needle. The aliquots were then frozen on dry ice for 1 hour, transferred to a lyophilization vessel, and lyophilized overnight. The next day, the aliquots were removed from the lyophilizer, wrapped in tin foil, and each microcentrifuge tube was immediately transferred to a 50 mL conical tube. The 50 mL conical tubes were sealed by tightening the lid and stored at -80 °C until use.

A 0.0025 µmol aliquot of HL647-Aβ$_{42}$ was removed from the -80 °C freezer and equilibrated to room temperature in a drawer. 10 mg/mL stock solutions of the 2AT and KLT TFA salts were prepared gravimetrically by dissolving 1.0 mg of each compound in 100 µL of deionized water that had been passed through a 0.2 µm filter. The 10 mg/mL solution of the 2AT TFA salt is equivalent to 1.49 mM 2AT. The 10 mg/mL solution of the KLT TFA salt is equivalent to 1.47 mM KLT. The 10 mg/mL stock solutions

were prepared in a 1.7 mL microcentrifuge tube and stored at -20 ºC when not in use.

In low-binding microcentrifuge tubes, a 40 μM solution of 2AT was created by adding 1.61 μL of the 1.49 mM 2AT solution to 58.39 μL filter sterilized deionized water, and a 40 μM solution of KLT was created by adding 1.63 μL of the 1.47 mM KLT solution to 58.37 μL filter sterilized deionized water. The 40 μM trimer solutions were then each diluted twofold by transferring 30 μL of the 40 μM solutions to 30 μL of deionized water in a new low-binding microcentrifuge tube to create 20 μM solutions of each trimer. 30 μL of each 20 μM trimer solution was then discarded, leaving 30 μL remaining in each tube. A fifth tube containing only 30 μL deionized water was also prepared.

A 1.112 μM solution of HL647-Aβ_{42} was prepared by first dissolving the 0.0025 μmol aliquot of HL647-Aβ_{42} in 14 μL filter-sterilized deionized water and sonicating the solution in a water bath sonicator for 5 min, and then transferring the 14 μL volume of HL647-Aβ_{42} to a 15 mL conical tube and diluting the solution with 2.226 mL of phenol red-free, serum-free DMEM:F12 media. A 270 μL portion of the 1.112 μM HL647-Aβ_{42} solution was transferred to each of the five previously prepared microcentrifuge tubes containing 40 μM and 20 μM 2AT and KLT or deionized water. The final volume in each tube was 300 μL and the final concentration of HL647-Aβ_{42} in each tube was 1 μM with either 0 μM, 2 μM, or 4 μM 2AT or KLT.

Treatment of the SH-SY5Y cells with HL647-Aβ_{42} in the absence or presence of 2AT and KLT. After the cells had adhered, the media was removed and replaced with 300 μL of the HL647-Aβ_{42} or HL647-Aβ_{42}/trimer solutions prepared in the previous step. The slide was then incubated at 37 °C in a 5% CO_2 atmosphere chamber for 16 hours. The media was removed from the wells and replaced with 200 μL of 1 μg/mL Hoechst 33342 in serum-free, phenol red-free DMEM:F12 media and allowed to incubate in the incubator for an additional 30 minutes.

The cells were imaged using a Keyence BZ-X810 fluorescence microscope. Z-stack images (10 μm total, with 1 μm pitch) were collected with a PlanApo 40x objective lens. Micrographs of treated cells

were recorded using the DAPI filter cube [excitation wavelength = 350/50 nm (325–375 nm) and emission wavelength = 460/50 nm (435–485nm)] for Hoechst 33342 nuclear marker and the Cy5 filter cube [excitation wavelength = 620/60 nm (590–650 nm) and emission wavelength = 700/75 nm (662.5–737.5 nm)] for HL647-Aβ_{42}. The images for all five micrographs in Figure 9 were acquired using identical acquisition parameters and processed identically. The Z-stack images were merged in the BZ-X810 Analyzer software. Integrated fluorescence intensity of HL647-Aβ_{42} and the number of Hoechst 33342 nuclei were quantified using the Hybrid Cell Count application in the BZ-X810 Analyzer software. For the integrated fluorescence intensity quantification of HL647-Aβ_{42}, dead cells were omitted from the analysis.

References and Notes

1 These procedures follow closely those that our laboratory has previously published. The procedures in this section are adapted from and in some cases taken verbatim from:

A. G. Kreutzer, S. Yoo, R. K. Spencer, J. S. Nowick, Stabilization, Assembly, and Toxicity of Trimers Derived from Aβ. *J. Am. Chem. Soc.* **139**, 966–975 (2017).

A. G. Kreutzer, I. L. Hamza, R. K. Spencer, J. S. Nowick, X-ray Crystallographic Structures of a Trimer, Dodecamer, and Annular Pore Formed by an Aβ17–36 β-Hairpin. *J. Am. Chem. Soc.* **138**, 4634–4642 (2016).

R. K. Spencer, A. G. Kreutzer, P. J. Salveson, H. Li, J. S. Nowick, X-ray Crystallographic Structures of Oligomers of Peptides Derived from β2-Microglobulin. *J. Am. Chem. Soc.* **137**, 6304–6311 (2015).

R. K. Spencer, H. Li, J. S. Nowick, X-ray crystallographic structures of trimers and higher-order oligomeric assemblies of a peptide derived from Aβ(17–36). *J. Am. Chem. Soc.* **136**, 5595–5598 (2014).

G. Guaglianone, *et al.*, Elucidating the Oligomerization and Cellular Interactions of a Trimer Derived from Aβ through Fluorescence and Mass Spectrometric Studies. *ACS Chem. Neurosci.* **13**, 2473–2482 (2022).

2 A. G. Kreutzer, S. Yoo, R. K. Spencer, J. S. Nowick, Stabilization, Assembly, and Toxicity of Trimers Derived from Aβ. *J. Am. Chem. Soc.* **139**, 966–975 (2017).

3 R. Bollhagen, M. Schmiedberger, K. Barlos, E. Grell, A new reagent for the cleavage of fully protected peptides synthesised on 2-chlorotrityl chloride resin. *J. Chem. Soc. Chem. Commun.* **0**, 2559–2560 (1994).

4 J. P. Tam, C. R. Wu, W. Liu, J. W. Zhang, Disulfide bond formation in peptides by dimethyl sulfoxide. Scope and applications. *J. Am. Chem. Soc.* **113**, 6657–6662 (1991).

5 O. Khakshoor, J. S. Nowick, Use of Disulfide "Staples" To Stabilize β-Sheet Quaternary Structure. *Org. Lett.* **11**, 3000–3003 (2009).

6 W. Kabsch, *XDS*. *Acta Crystallographica Section D Biological Crystallography* **66**, 125–132 (2010).

7 P. D. Adams, *et al.*, PHENIX: a comprehensive Python-based system for macromolecular structure solution. *Acta Crystallogr. D Biol. Crystallogr.* **66**, 213–221 (2010).

8 P. Emsley, B. Lohkamp, W. G. Scott, K. Cowtan, Features and development of *Coot*. *Acta Crystallographica Section D Biological Crystallography* **66**, 486–501 (2010).

9 J. Painter, E. A. Merritt, Optimal description of a protein structure in terms of multiple groups undergoing TLS motion. *Acta Crystallogr. D Biol. Crystallogr.* **62**, 439–450 (2006).

10 J. Painter, E. A. Merritt, TLSMD web server for the generation of multi-group TLS models. *J. Appl. Crystallogr.* **39**, 109–111 (2006).

11 H. Schägger, Tricine–SDS-PAGE. *Nat. Protoc.* **1**, 16–22 (2006).

12 R. J. Simpson, Staining proteins in gels with silver nitrate. *CSH Protoc.* **2007**, db.prot4727 (2007).

13 M. T. Marty, *et al.*, Bayesian deconvolution of mass and ion mobility spectra: from binary interactions to polydisperse ensembles. *Anal. Chem.* **87**, 4370–4376 (2015).

Characterization Data

Characterization of 2AT

LC/MS of 2AT:

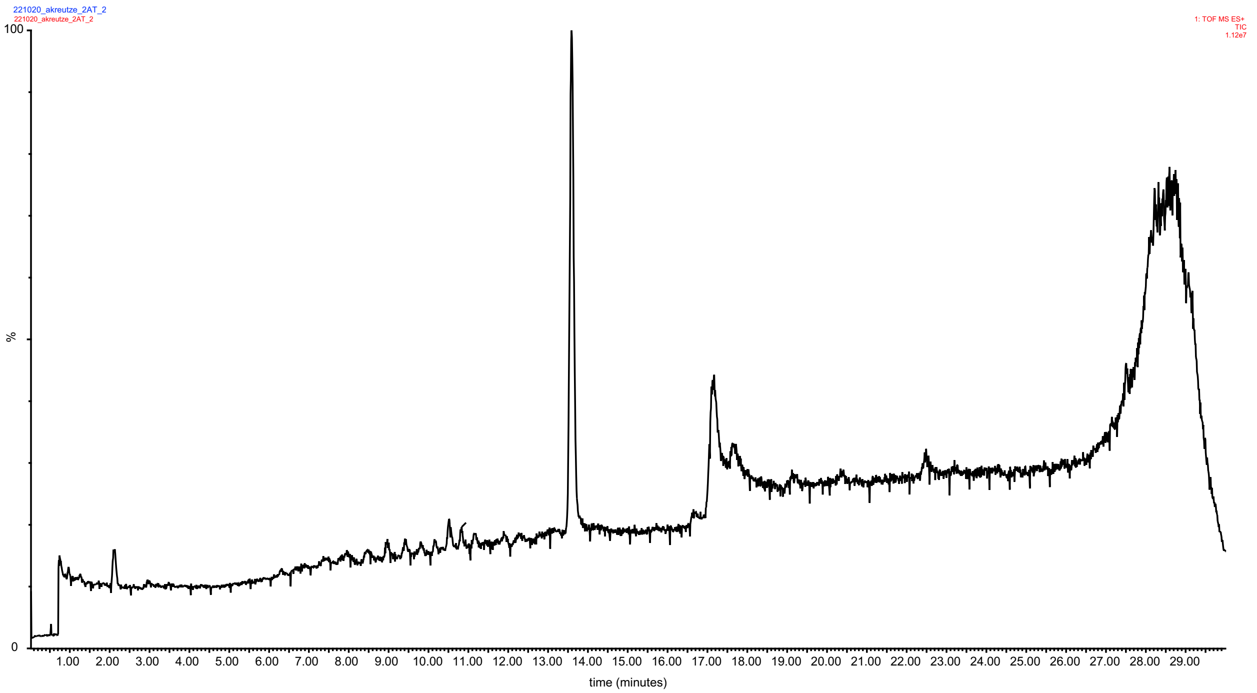

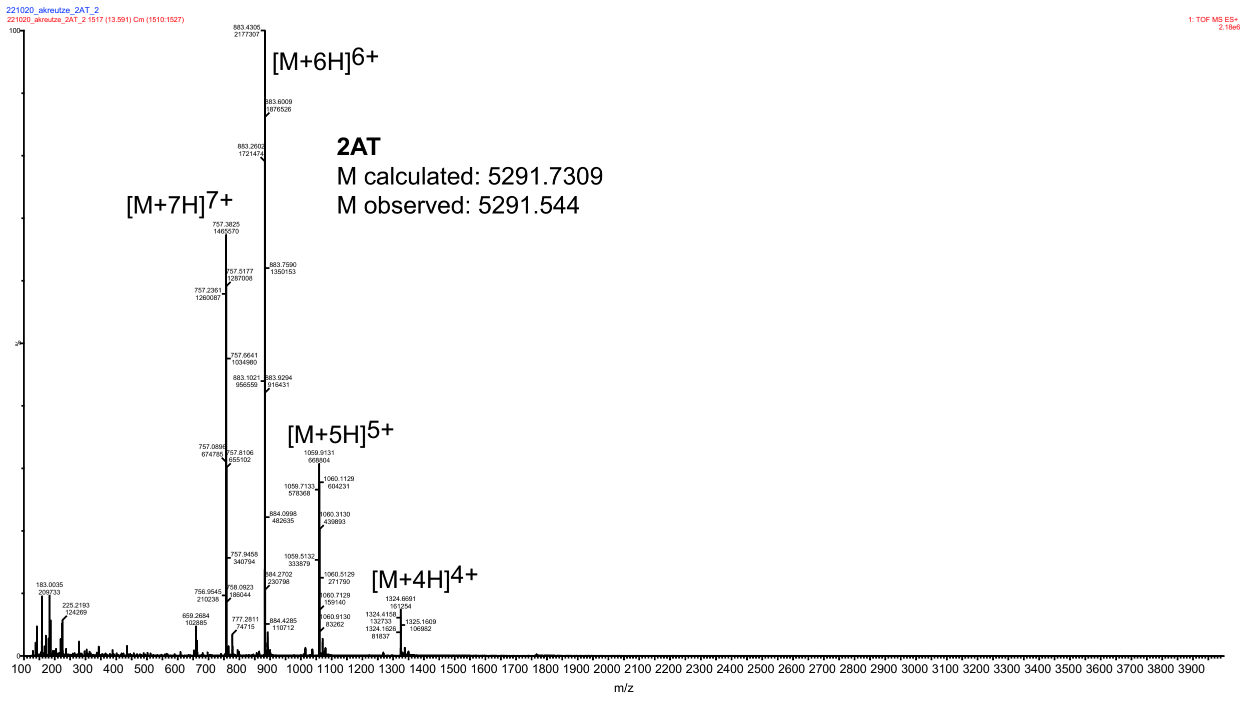

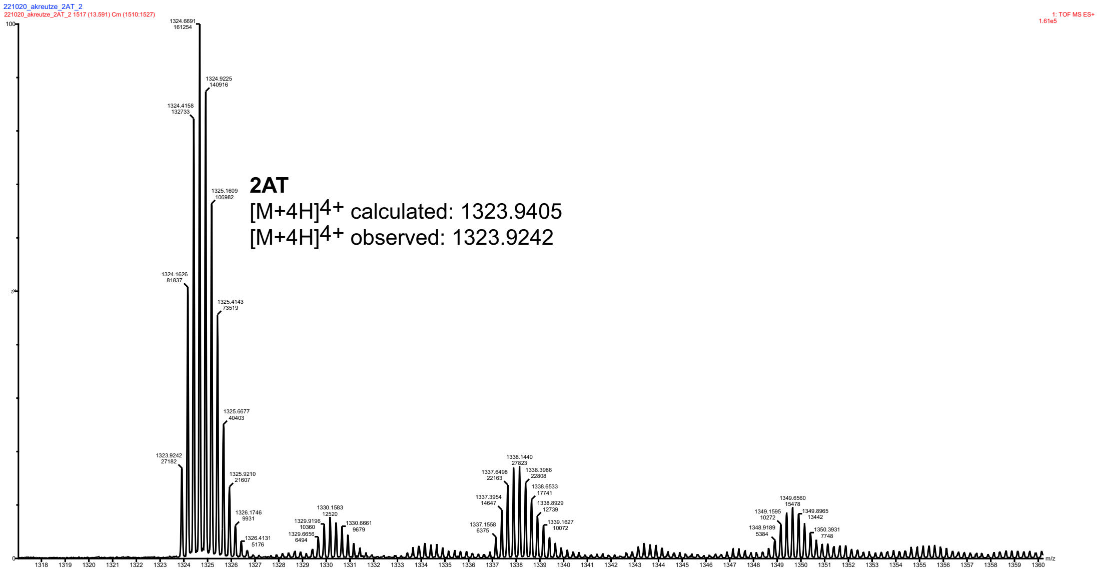
2AT
[M+4H]4+ calculated: 1323.9405
[M+4H]4+ observed: 1323.9242

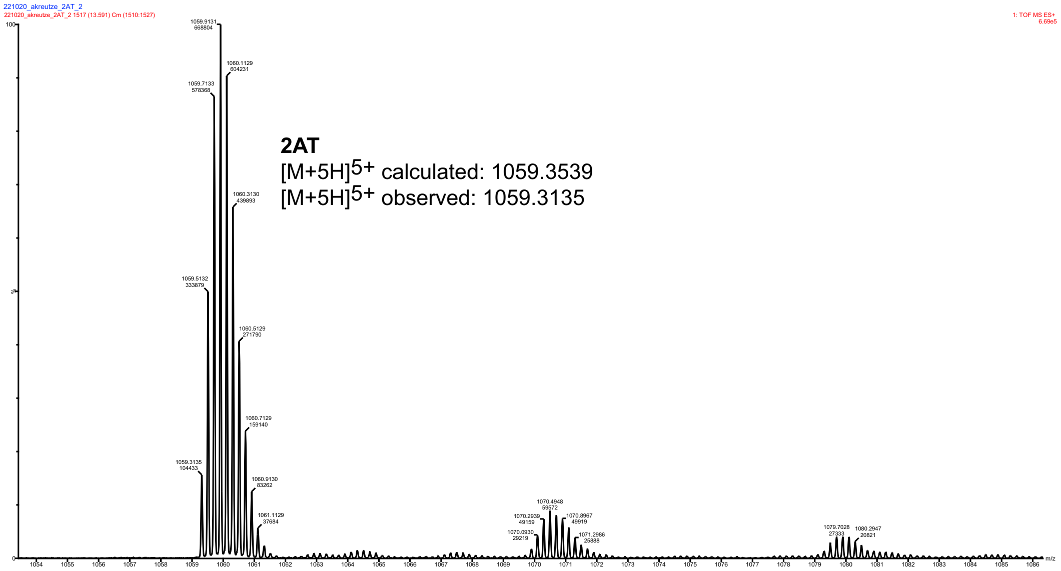
2AT
[M+5H]5+ calculated: 1059.3539
[M+5H]5+ observed: 1059.3135

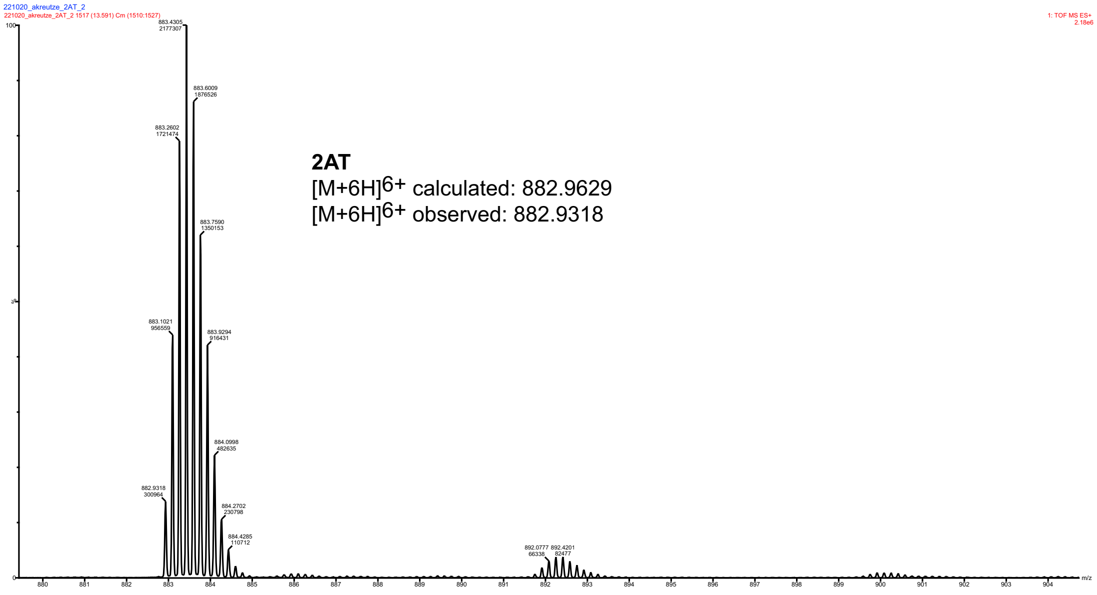
2AT
[M+6H]6+ calculated: 882.9629
[M+6H]6+ observed: 882.9318

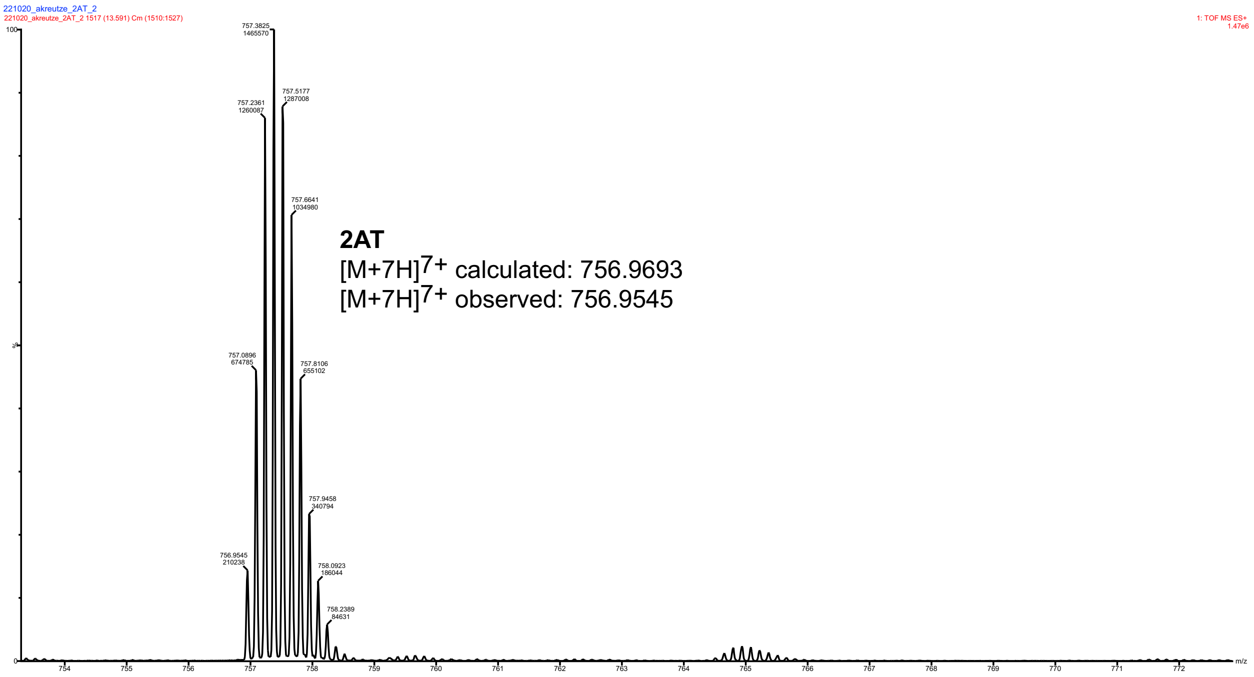
221020_akreutze_2AT_2
221020_akreutze_2AT_2 1517 (13.591) Cm (1510:1527)
1: TOF MS ES+
1.47e6
2AT
[M+7H]7+ calculated: 756.9693
[M+7H]7+ observed: 756.9545
m/z

Characterization of KLT

LC/MS of KLT:

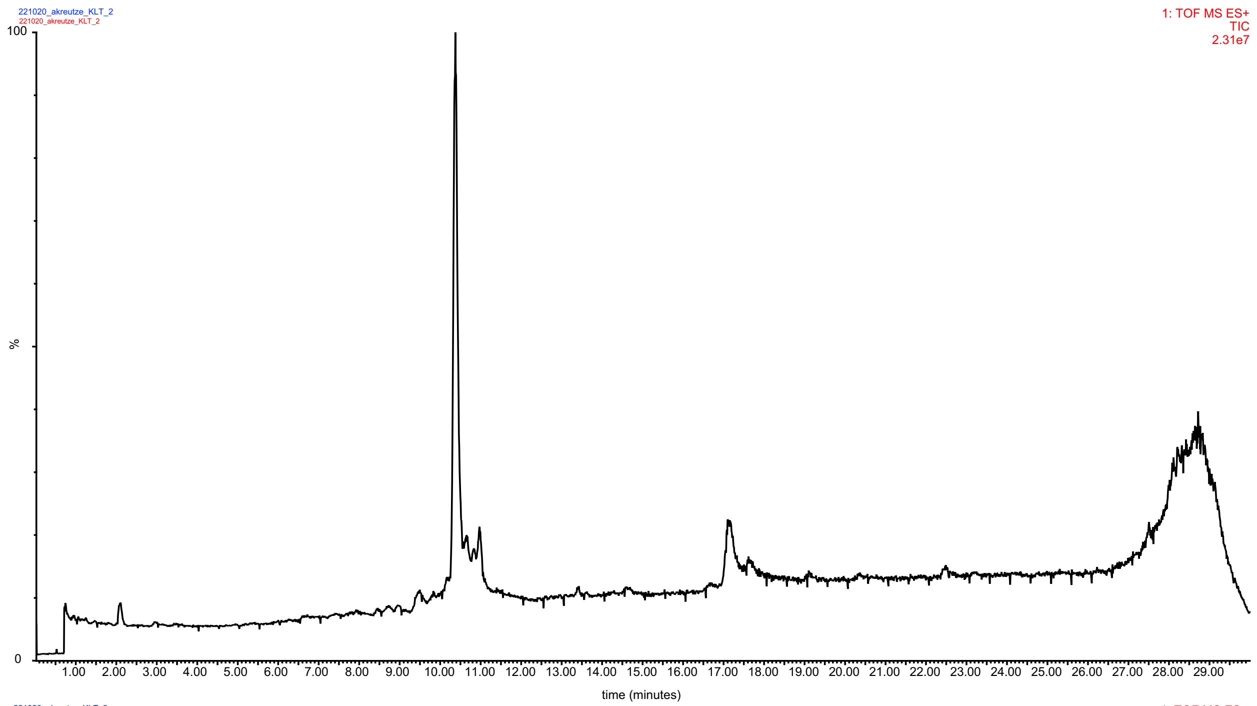

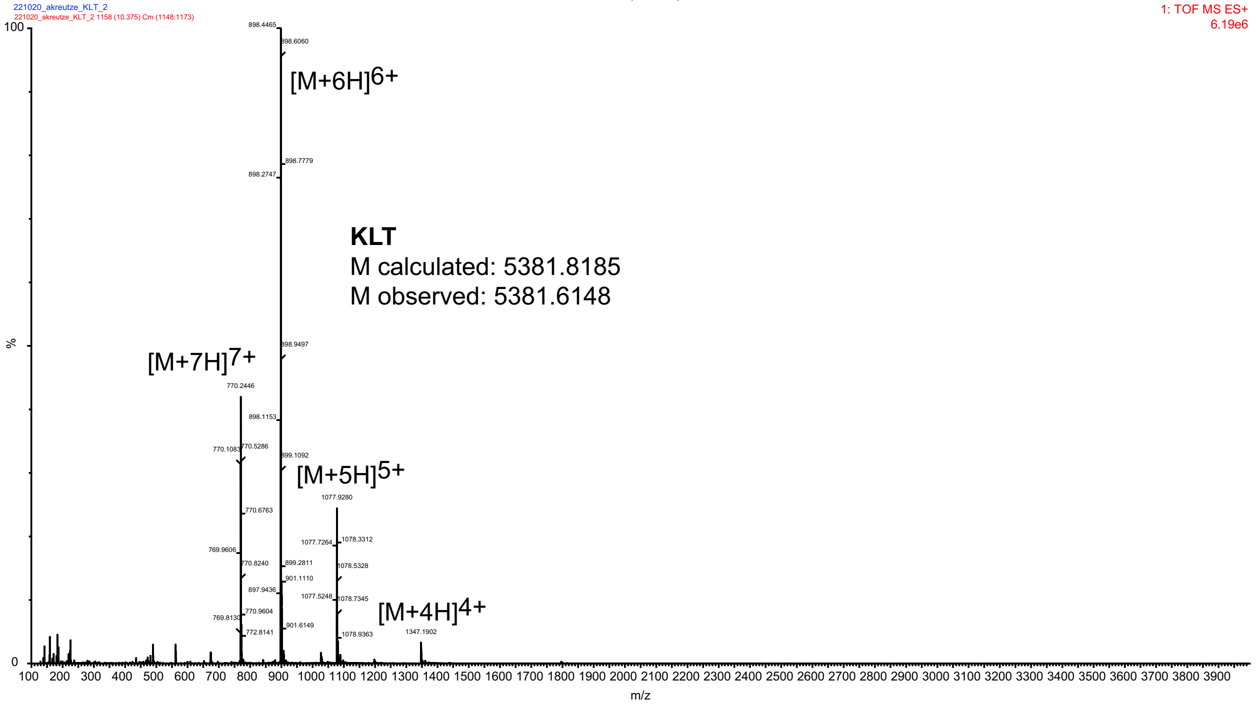

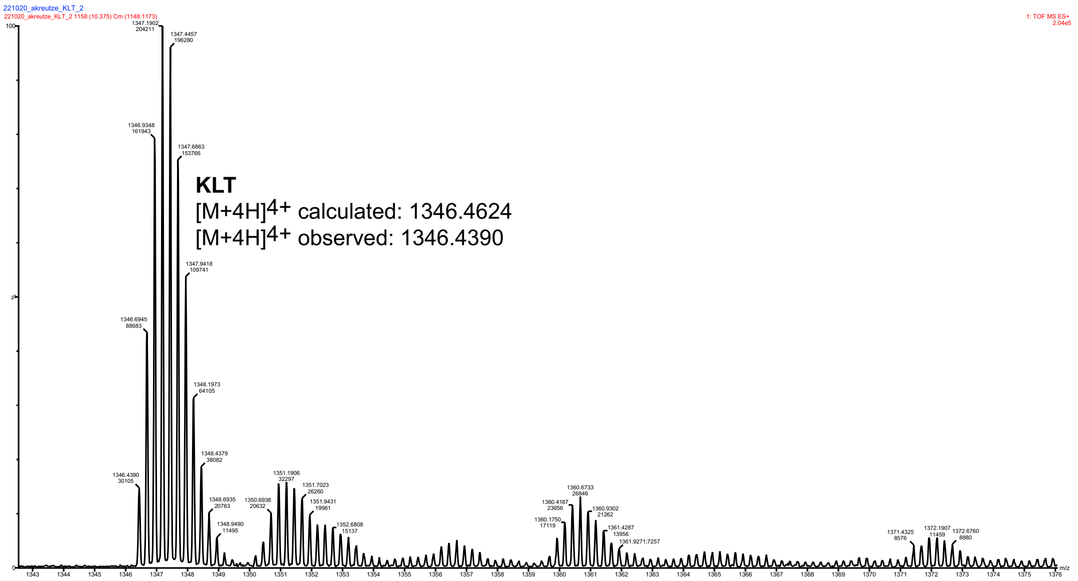

221020_akreutze_KLT_2
KLT
[M+4H]4+ calculated: 1346.4624
[M+4H]4+ observed: 1346.4390

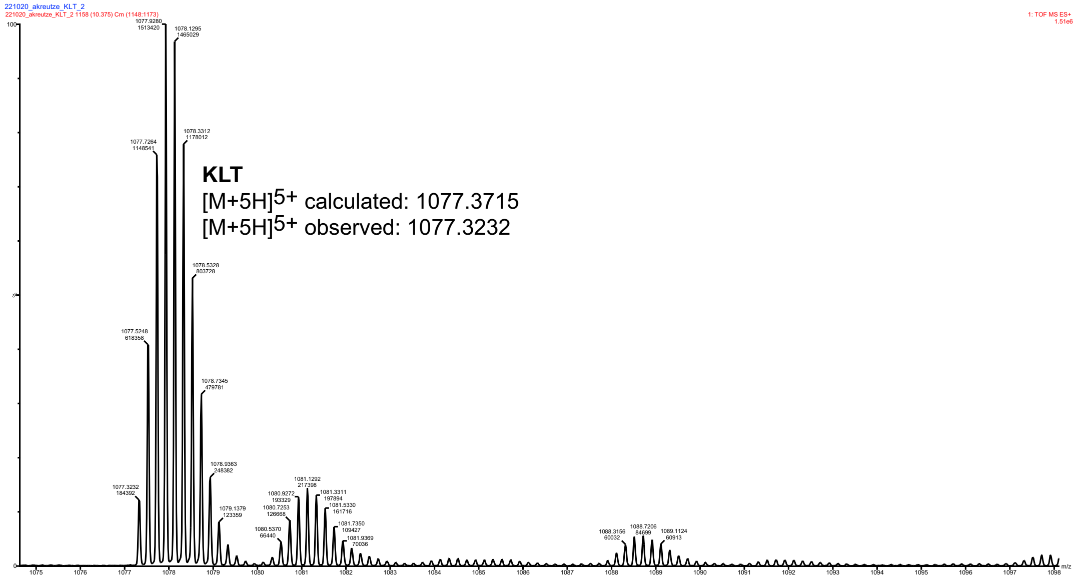

221020_akreutze_KLT_2
KLT
[M+5H]5+ calculated: 1077.3715
[M+5H]5+ observed: 1077.3232

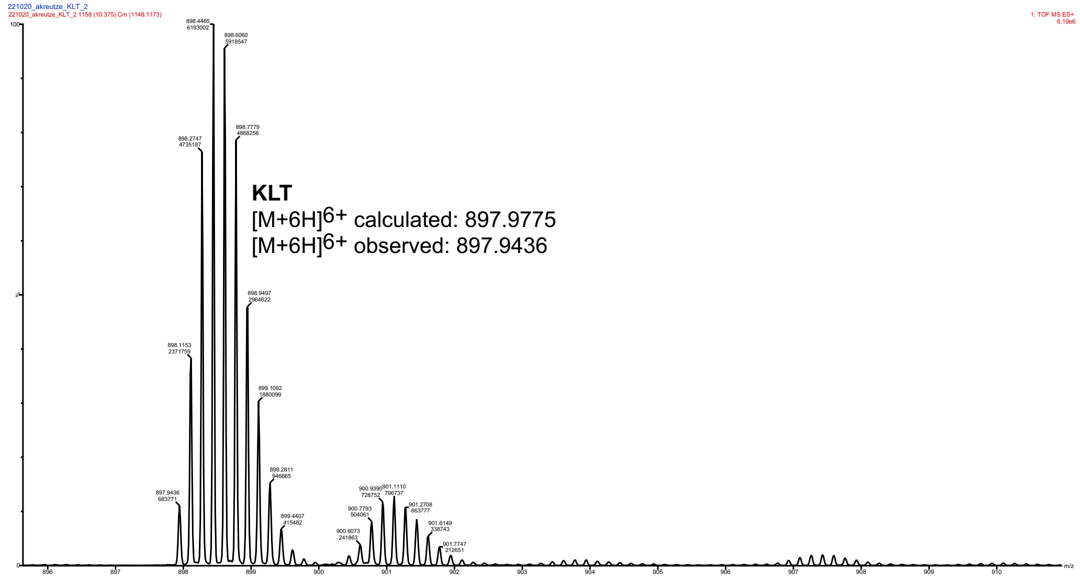

221020_akreutze_KLT_2
KLT
[M+6H]6+ calculated: 897.9775
[M+6H]6+ observed: 897.9436

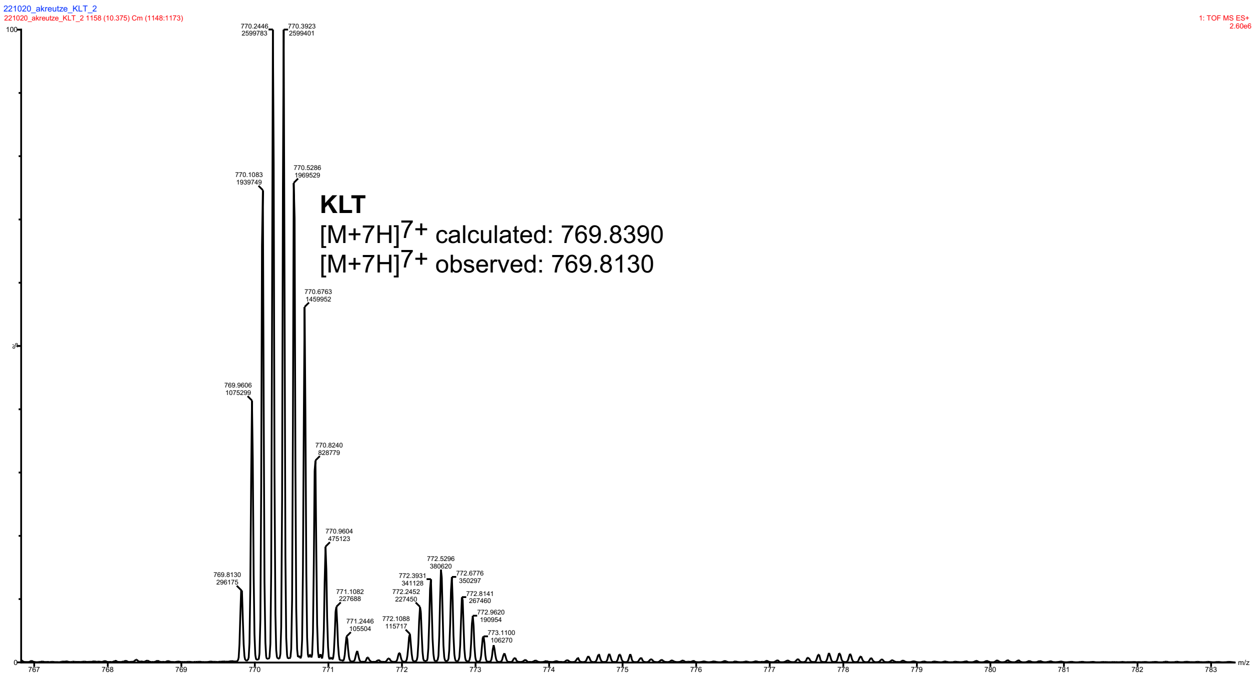

221020_akreutze_KLT_2
221020_akreutze_KLT_2 1158 (10.375) Cm (1148:1173)
1: TOF MS ES+
2.60e6
KLT
[M+7H]7+ calculated: 769.8390
[M+7H]7+ observed: 769.8130
770.2446
2599783
770.3923
2599401
770.1083
1939749
770.5286
1969529
770.6763
1459952
769.9606
1075299
770.8240
828779
770.9604
475123
769.8130
296175
771.1082
227688
771.2446
105504
772.5296
380620
772.3931
341126
772.6776
350297
772.2452
227450
772.8141
267460
772.1088
115717
772.9620
190954
773.1100
106270
m/z

Characterization of 2AT-sCy3

Analytical HPLC traces:

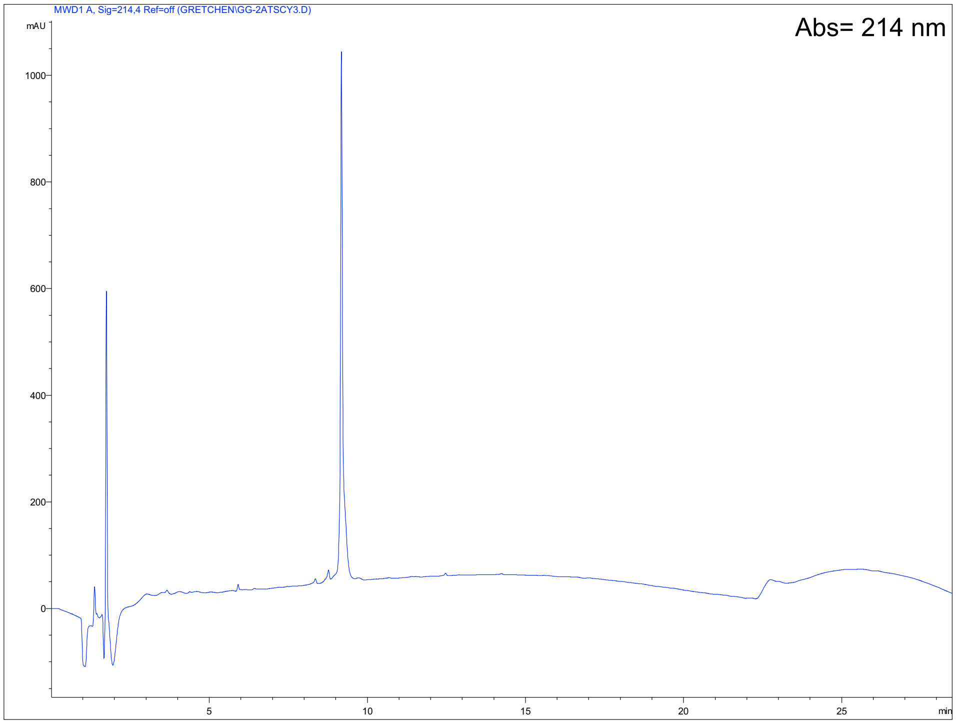

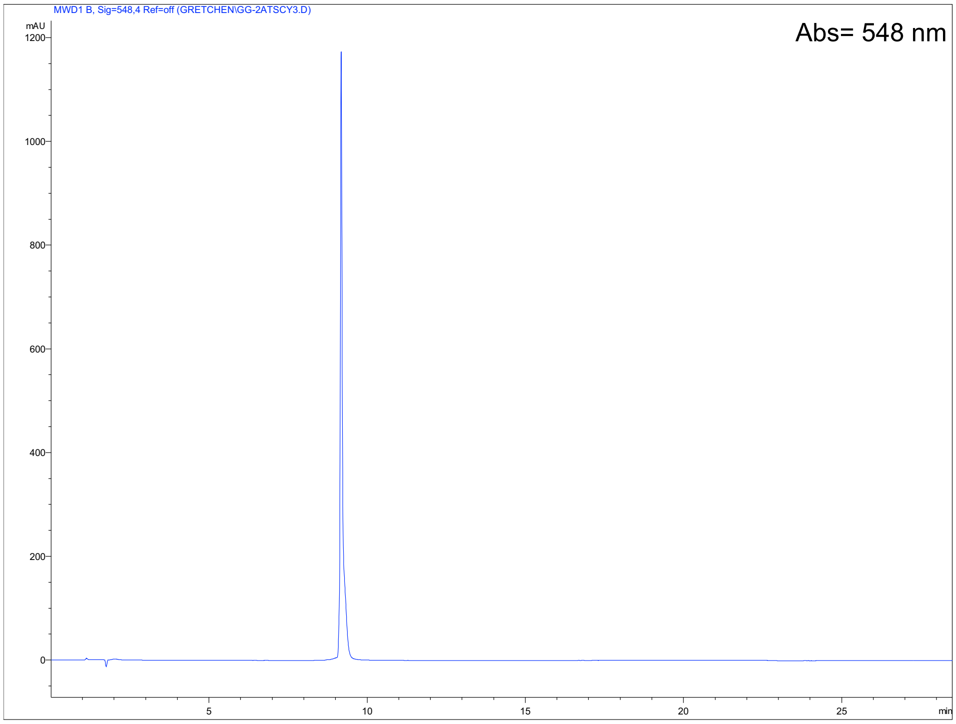

Mass spectrum of 2AT-sCy3

Calculated $[M+H]^+$ of 2AT-sCy3: 5890.92

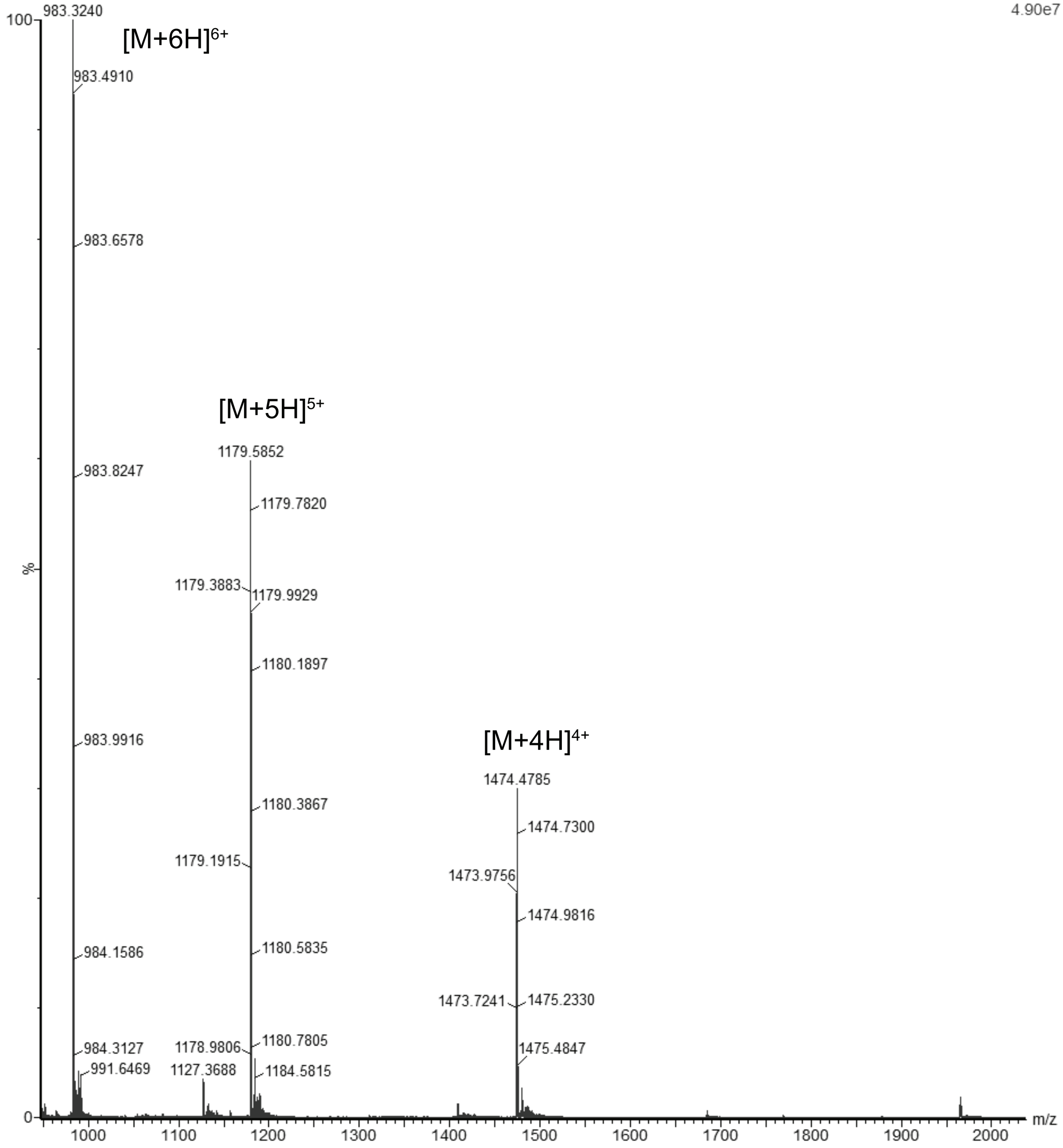

Deconvolution mass spectrum of 2AT-sCy3

Exact mass of 2AT-sCy3: 5889.91

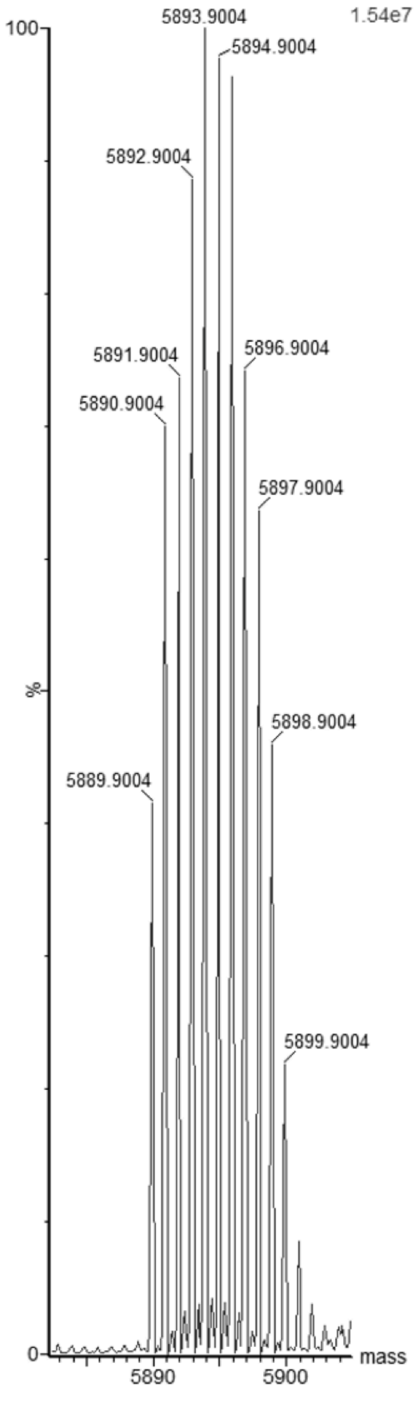

Characterization of KLT-sCy3

Analytical HPLC trace

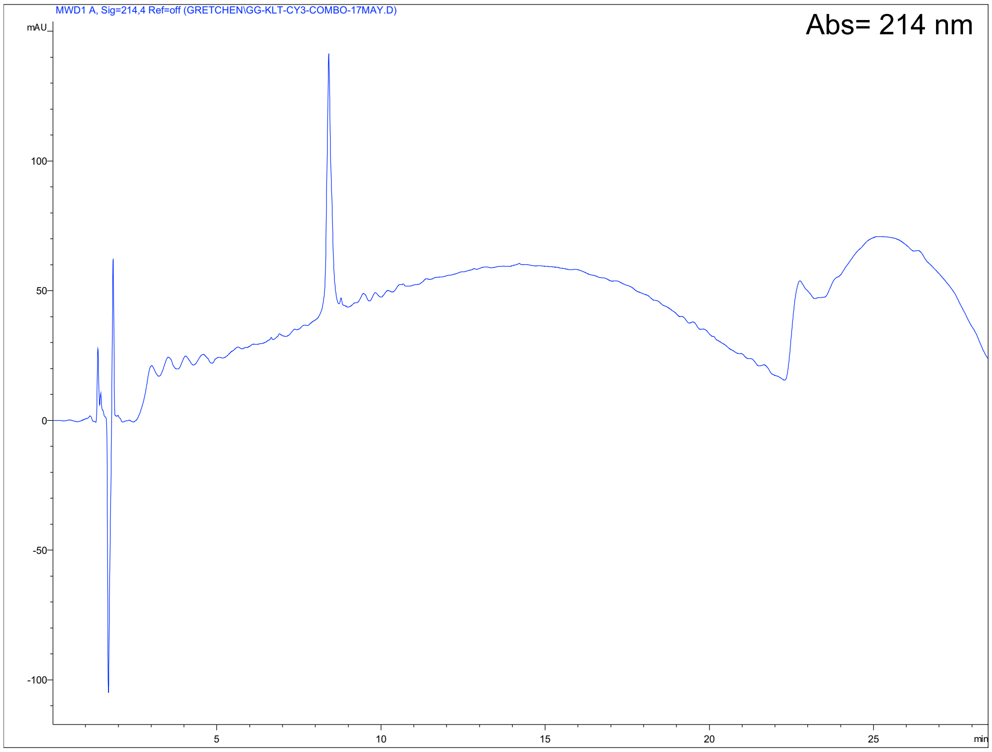

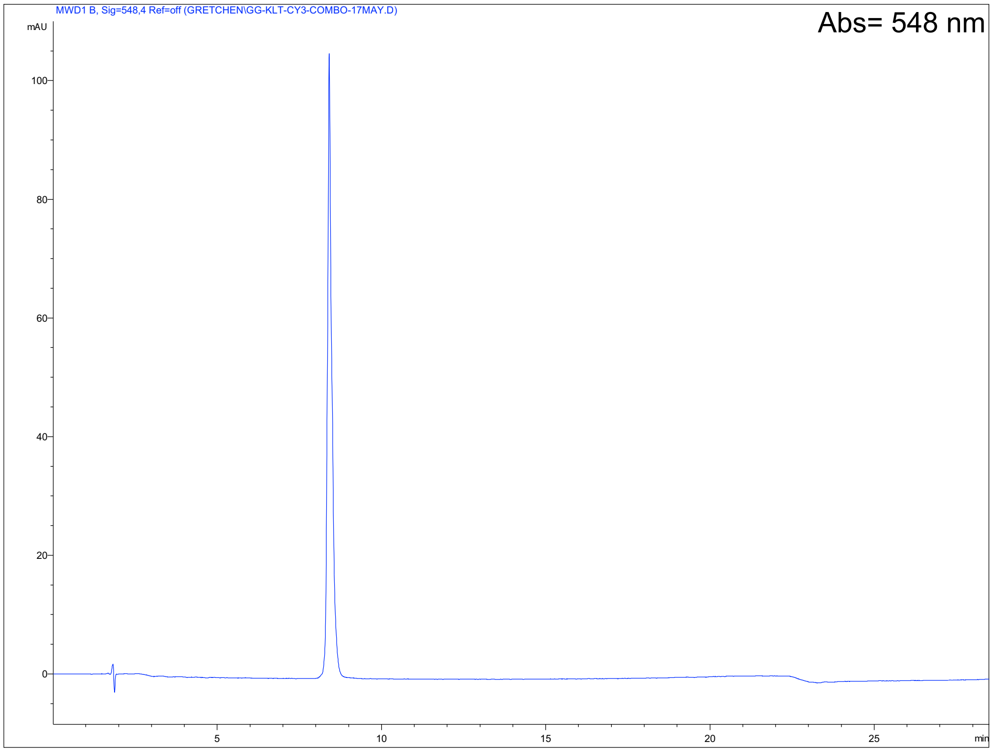

Mass spectrum of KLT-sCy3

Calculated $[M+H]^+$ of KLT-sCy3: 5980.01

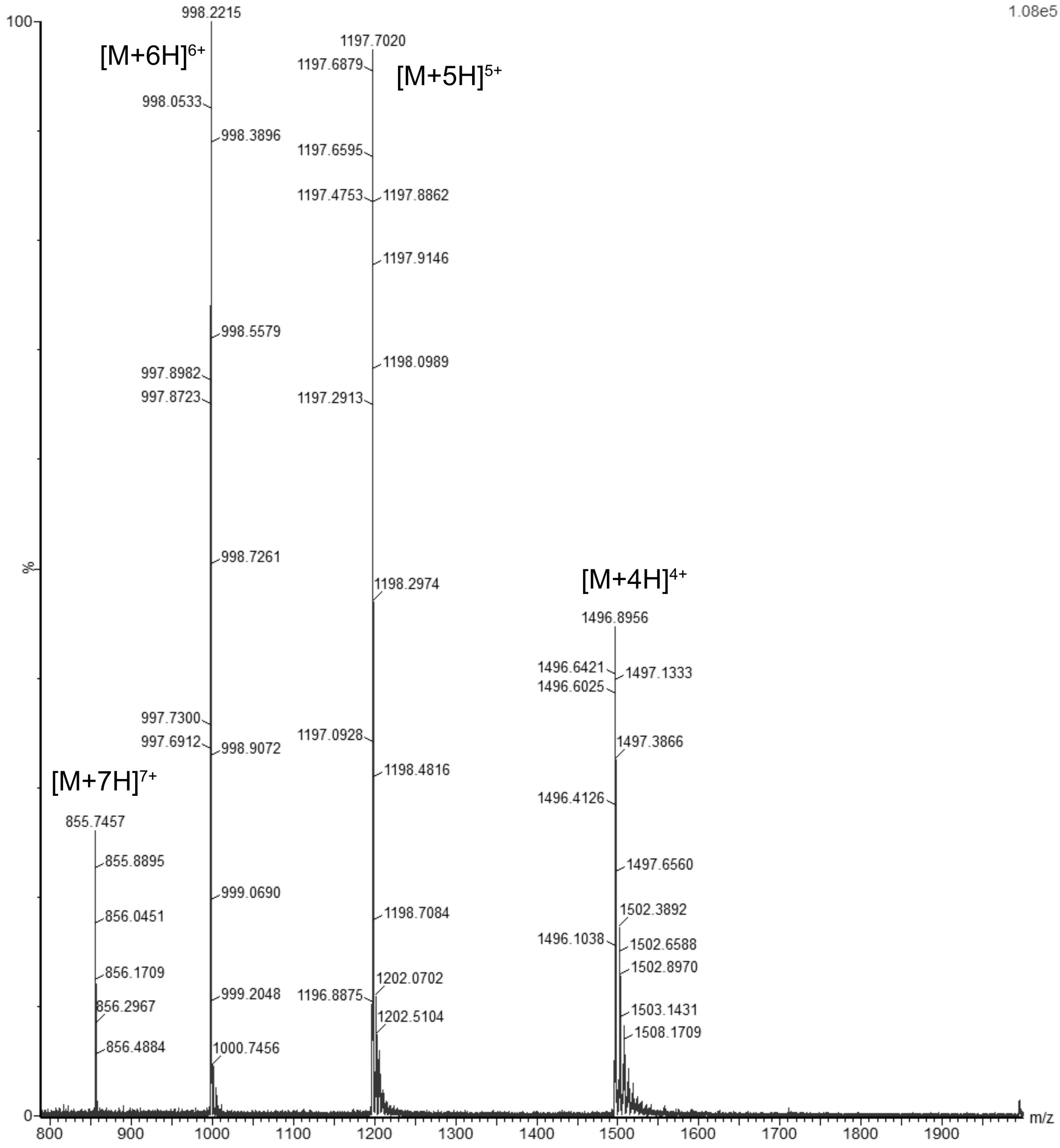

Deconvolution mass spectrum of KLT-sCy3

Exact mass of KLT-sCy3: 5979.99

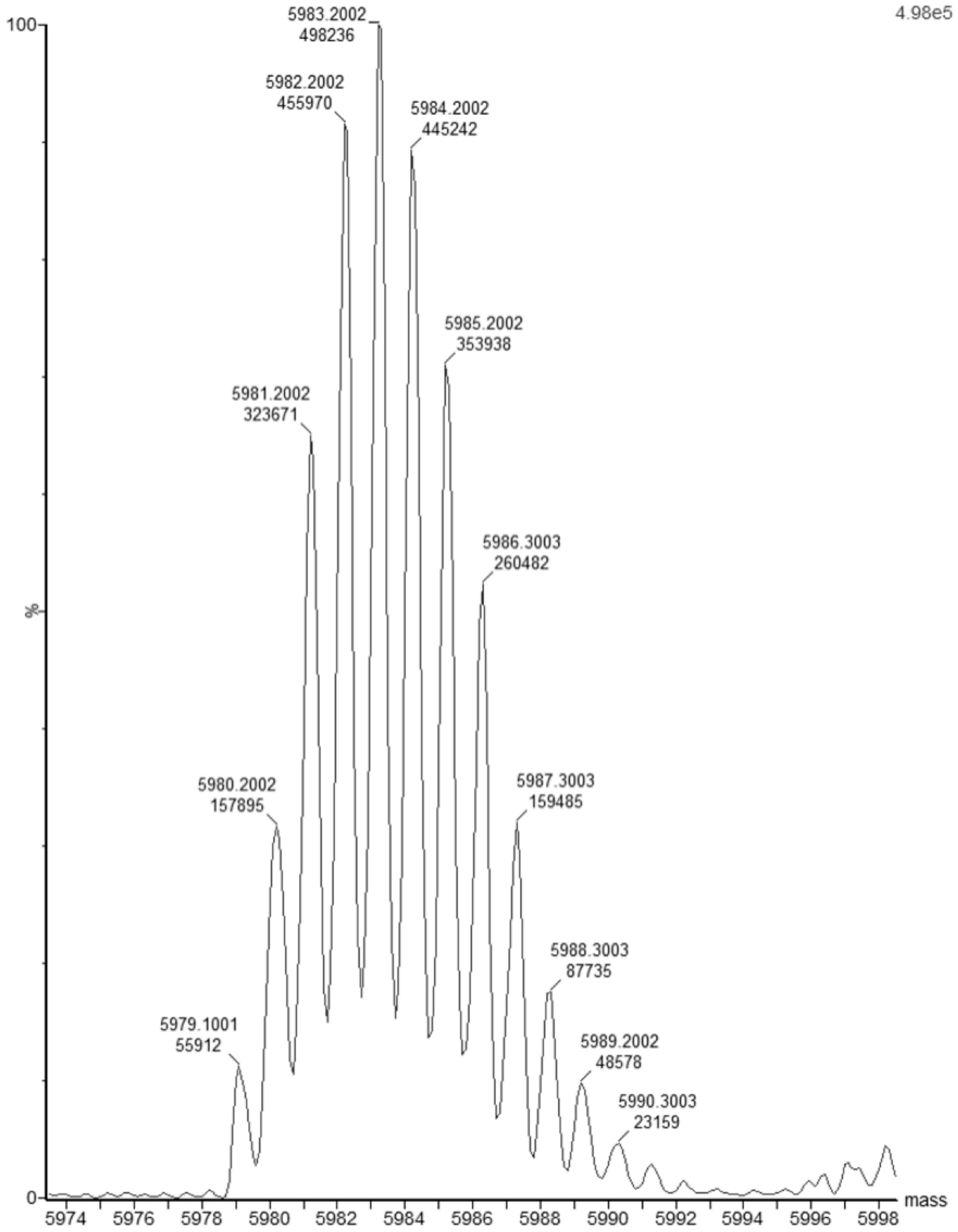

Supplement: Supplementary file 1 — Appendix 01 (PDF) [file pnas.2219216120.sapp.pdf]
